# Skin-derived myeloid precursors and joint-resident fibroblasts spread psoriatic disease from skin to joints

**DOI:** 10.1038/s41590-025-02351-z

**Published:** 2026-01-02

**Authors:** Maria G. Raimondo, Hashem Mohammadian, Mario R. Angeli, Stefano Alivernini, Vladyslav Fedorchenko, Kaiyue Huang, Richard Demmler, Peter Rhein, Cong Xu, Yi-Nan Li, Raphael Micheroli, Zoltán Winter, Aleix Rius Rigau, Charles G. Anchang, Alina Soare, Markus Luber, Hannah Labinsky, Jiyang Chang, Claudia Günther, Ursula Fearon, Douglas J. Veale, Francesco Ciccia, Jürgen Rech, Michael Sticherling, Tobias Bäuerle, Jörg H. W. Distler, Mariola S. Kurowska-Stolarska, Matthias Mack, Arif B. Ekici, Adam P. Croft, Oliver Distler, Hans M. Maric, Caroline Ospelt, Juan D. Cañete, Maria A. D’Agostino, Georg Schett, Simon Rauber, Andreas Ramming

**Affiliations:** 1 Department of Medicine 3—Rheumatology and Immunology, Friedrich-Alexander-Universität (FAU) Erlangen-Nürnberg and Uniklinikum Erlangen, Erlangen, Germany; 2 Deutsches Zentrum Immuntherapie (DZI), Friedrich-Alexander-Universität (FAU) Erlangen-Nürnberg and Uniklinikum Erlangen, Erlangen, Germany; 3 Division of Rheumatology, Università Cattolica del Sacro Cuore, Fondazione Policlinico Universitario Agostino Gemelli IRCCS, Rome, Italy; 4 Immunology Core Facility, Gemelli Science and Technology Park, Fondazione Policlinico Universitario Agostino Gemelli IRCCS, Rome, Italy; 5 Applications, Cytek Biosciences, Amsterdam, the Netherlands; 6 Clinic for Rheumatology, University Hospital Düsseldorf, Heinrich-Heine University Düsseldorf, Düsseldorf, Germany; 7 Hiller Research Center, University Hospital Düsseldorf, Heinrich-Heine University Düsseldorf, Düsseldorf, Germany; 8 Center of Experimental Rheumatology, Department of Rheumatology, University Hospital of Zurich and University of Zurich, Zurich, Switzerland; 9 Institute of Radiology, Friedrich-Alexander-Universität (FAU) Erlangen-Nürnberg and Uniklinikum Erlangen, Erlangen, Germany; 10 Department of Dermatology, University Hospital Carl Gustav Carus Dresden and TU Dresden, Dresden, Germany; 11 Department of Dermatology, University Hospital, Eberhard Karls University Tübingen, Tübingen, Germany; 12 Molecular Rheumatology, Trinity Biomedical Sciences Institute, Trinity College Dublin, Dublin, Ireland; 13 EULAR Centre of Excellence for Rheumatology, Centre for Arthritis and Rheumatic Diseases, St. Vincent’s University Hospital, Dublin, Ireland; 14 Department of Precision Medicine, Division of Rheumatology, Università della Campania L. Vanvitelli, Naples, Italy; 15 Department of Dermatology, Friedrich-Alexander-Universität (FAU) Erlangen-Nürnberg and Uniklinikum Erlangen, Erlangen, Germany; 16 Department of Radiology, Johannes-Gutenberg-Universität Mainz and Universitätsmedizin Mainz, Mainz, Germany; 17 Research into Inflammatory Arthritis Centre Versus Arthritis (RACE), University of Glasgow, Glasgow, UK; 18 School of Infection and Immunity, College of Medical, Veterinary and Life Sciences, University of Glasgow, Glasgow, UK; 19 Department of Nephrology, University Hospital Regensburg and University of Regensburg, Regensburg, Germany; 20 Institute of Human Genetics, Friedrich-Alexander-Universität (FAU) Erlangen-Nürnberg and Uniklinikum Erlangen, Erlangen, Germany; 21 Rheumatology Research Group, Institute for Inflammation and Ageing, College of Medical and Dental Sciences, University of Birmingham, Birmingham, UK; 22 NIHR Birmingham Biomedical Research Centre and Clinical Research Facility, Queen Elizabeth Hospital, University of Birmingham, Birmingham, UK; 23 Rudolf-Virchow-Center for Integrative and Translational Imaging, University of Würzburg, Würzburg, Germany; 24 Department of Rheumatology, Hospital Clínic of Barcelona and Fundació Clinic per la Recerca Biomèdica-IDIBAPS, Barcelona, Spain

**Keywords:** Psoriasis, Chronic inflammation, Cell migration

## Abstract

Psoriatic disease initially affects the skin and later extends to the joints. Here, we show a two-step process that orchestrates the spread of inflammation from the skin to the joints. Induction of psoriatic skin disease in photoconvertible mice, followed by sequencing and computational characterization of skin-derived cells in the joints, was used to identify a population of CD2^+^MHC-II^+^CCR2^+^ myeloid precursors that builds a skin-derived myeloid cell compartment in the joints. Single-cell cross-species reference mapping and mitochondrial variant tracing showed an orthologous human cell population. Interactome analysis of the joints showed that in a second step, resident regulatory CD200^+^ fibroblasts regulate the priming of CD2^+^MHC-II^+^CCR2^+^ myeloid precursors, which subsequently control IL-17 expression in T cells. Hence, the spread of inflammation requires a distinct migratory myeloid precursor population and a permissive local tissue environment, similar to tumor metastasis.

## Abstract (web summary)

How inflammation spreads from the skin to the joints in psoriatic disease is unclear. Here, the authors show that joint-resident fibroblasts can control differentiation of infiltrating skin-derived myeloid precursors that can become inflammatory macrophages.

## Main

Psoriatic disease is an immune-mediated inflammatory disease characterized by inflammation of the skin and joints^1^. It can manifest in different ways, but skin inflammation (psoriasis) typically precedes joint inflammation (psoriatic arthritis (PsA)) in about 80% of individuals^2^, with approximately 30% of individuals with psoriasis developing arthritis^3^. This progression suggests a directed spatio-temporal link between these two organs, although the underlying mechanism of this spread remains largely unknown.

At the molecular level, psoriatic disease is associated with increased activity of interleukin-23/interleukin-17 (IL-23/IL-17) signaling as well as tumor necrosis factor (TNF). For instance, the systemic overexpression of IL-23 in autoimmune-prone B10.RIII mice results in psoriasis-like skin and joint disease^4^. Similarly, the inducible overexpression of human TNF leads to psoriasis-like skin and musculoskeletal manifestations^5^. Although neutralizing these cytokines has proven to be an effective clinical treatment for alleviating psoriatic symptoms^6^, these therapies do not clarify how inflammation spreads from the skin to the joints.

Genome-wide association studies have identified genetic variants associated with psoriasis and PsA, including *HLA-C* and *HLA-B*, respectively^7^. However, there is currently no reliable molecular mechanism or biomarker that can predict which individuals with psoriasis will develop PsA^8^. The sequential progression of the disease suggests a mechanistic axis between the skin and joints. Recent studies have demonstrated that skin-derived tissue-resident memory T cells recirculate into the blood in individuals with PsA^9,10^, and clonal expansion of tissue-resident T cells poised to produce IL-17 from lesional to nonlesional psoriatic skin has been observed^11–13^. Although monocyte recirculation may be dependent on the junctional adhesion molecule JAM3 (ref. ^14^), it remains unclear whether other immune cell subsets migrate by this mechanism and whether they can migrate between distinct peripheral tissue compartments such as the skin and joints. A systematic and unbiased analysis of cell migration in psoriatic disease has not been undertaken, leaving the mechanisms by which inflammation spreads between organs unresolved.

Here, we show the recirculation of skin-derived proinflammatory CD2^+^major histocompatibility complex class II (MHC-II)^+^CCR2^+^ myeloid precursors into the joints in psoriatic disease. This recirculation contributes to the spreading of psoriatic disease to the joints and requires a local mesenchymal permissive microenvironment in the joints to override the CD200 immune checkpoint to establish full inflammation in the joints.

## Results

### IL-23-induced psoriasis drives leukocyte migration from the skin to joints

**Fig. 1:**
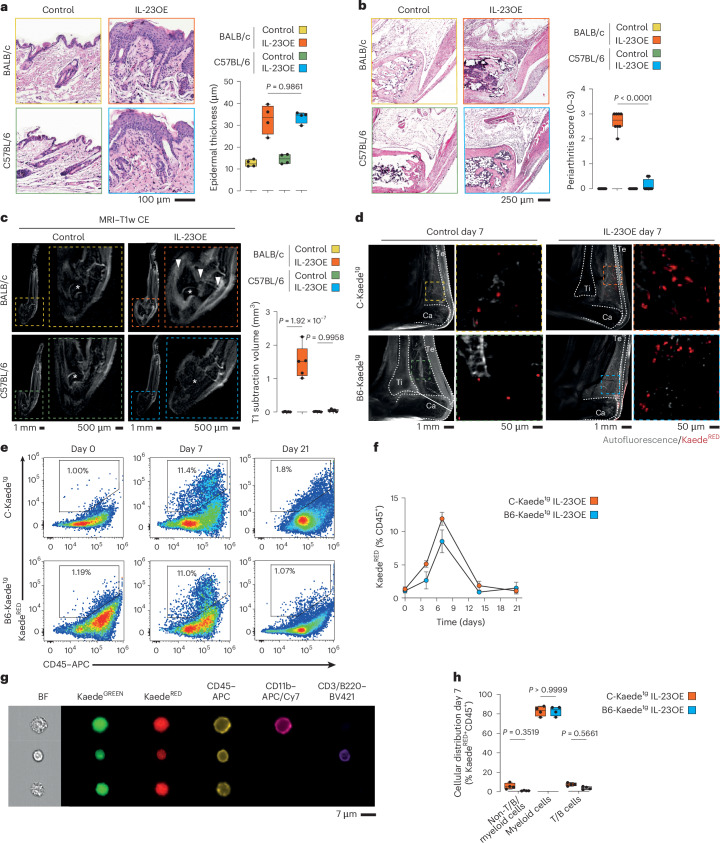
CD11b^+^ myeloid cells migrate from the skin to joints in a model of psoriatic disease. **a**, Left, representative micrographs of H&E-stained skin sections of the hind paws of BALB/c and C57BL/6 mice at day 21 with and without IL-23OE. Right, quantification of epidermal thickness at day 21. The graph shows the median, quartiles and minimum–maximum; *N* = 4 per condition. *P* values were calculated by one-way analysis of variance (ANOVA) with a Tukey’s post hoc test. **b**, Left, representative micrographs of H&E-stained ankle sections of BALB/c and C57BL/6 mice at day 21 with and without IL-23OE. Right, quantification of arthritis at day 21. The graph shows the median, quartiles and minimum–maximum; *N* = 8 per condition. *P* values were calculated by one-way ANOVA with a Tukey’s post hoc test. **c**, Representative micrographs of MRI-scanned ankles of BALB/c and C57BL/6 mice at day 21 with and without IL-23OE used for the quantification of arthritis at day 21. Arrowheads indicate inflammation, and stars indicate the talar bone. The graph shows the median, quartiles and minimum–maximum; *N* = 5 per condition. *P* values were calculated by one-way ANOVA with a Tukey’s post hoc test; T1w CE, T1-weighted contrast-enhanced. **d**, Representative micrographs of light sheet fluorescence microscopy of Kaede^tg^ ankles from BALB/c (C-Kaede^tg^) and C57BL/6 (B6-Kaede^tg^) background strains at day 7 with and without IL-23OE and after photoconversion of cells localized in the skin. Arrowheads indicate accumulations of photoconverted Kaede^RED^ cells. Graphical drawings of the tibia (Ti), calcaneus (Ca) and Achilles tendon (Te) are included. **e**, Representative flow cytometry plots for the quantification of Kaede^RED^ skin-derived cells in the joint. **f**, Quantification of Kaede^RED^ skin-derived cells in the joints. The graph shows the mean and standard error of the mean; *N* = 4 per time point and condition. **g**, Representative micrographs of imaging flow cytometry for the typing of Kaede^RED^ skin-derived cells in the joint at day 7; BF, brightfield. **h**, Quantification of CD45^+^ Kaede^RED^ skin-derived cell types in the joints at day 7. The graph shows medians, quartiles and minimum–maximum; *N* = 4 per condition. *P* values were calculated by one-way ANOVA with a Tukey’s post hoc test. Source data

**Extended Data Fig. 1:**
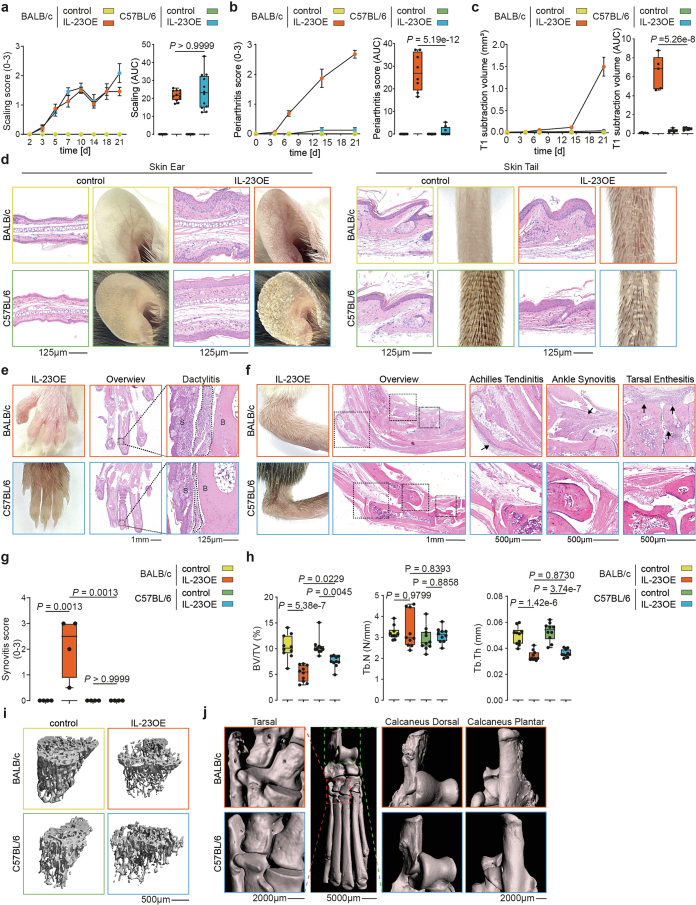
Assessment of psoriasis and arthritis over time. **(a)** Macroscopic evaluation of skin-scaling in mice with/without IL-23OE for 21 days (left). Total scaling quantified as area under the curve (AUC, right). Graphs: mean ± SEM (left) and median, quartiles, min-max (right); *N* = 12 per condition. *P* values calculated by one-way ANOVA with Tukey´s post hoc test. **(b)** Histological inflammation scores for ankle regions based on H&E staining in mice with/without IL-23OE for 21 days (left). Quantification of arthritis as AUC (right). Graphs: mean ± SEM (left) and median, quartiles, min-max (right); *N* = 8 per time point and condition. *P* values calculated by one-way ANOVA with Tukey´s post hoc test. **(c)** MRI ankle measurements over time in mice with/without IL-23OE for 21 days (left). Quantification of arthritis as AUC (right). Graphs: mean ± SEM (left) and median, quartiles, min-max (right); *N* = 5 per condition. *P* values calculated by one-way ANOVA with Tukey´s test. **(d-f)** Representative micrographs of H&E-stained sections and representative photographs from skin of ear and tail **(d)**, distal paws **(e)** and ankles **(f)** of mice at day 21 with/without IL-23OE. In **(e)**: dashed lines highlight dactylitis; S = skin, B = bone. In **(f)**: dashed squares in the overview identify the areas of corresponding magnifications; black arrows indicate sites of inflammation. Images representative for three independent experiments with similar results. **(g)** Histological inflammation score of synovia in ankle and tarsal joints based on H&E-stainings in mice with/without IL-23OE for 21 days. Graphs: median, quartiles, min-max.; *N* = 4 per condition; *P* values calculated by one-way ANOVA with Tukey´s post hoc test. **(h)** Micro-CT quantification of tibia from mice at day 21 with IL-23 OE. BV/TV = bone volume/total volume; Tb. N = trabecular number; Tb. Th = trabecular thickness. Graphs: median, quartiles, min-max; *N* = 10 per condition; *P* values calculated by one-way ANOVA with Tukey´s post hoc test. **(i)** Representative micro-CT images of the tibial trabecular network of mice at day 21 with/without IL-23 OE. **(j)** Representative micro-CT images from hind paws of mice at day 21 with/without IL-23OE. Red and green dashed squares indicating corresponding magnification of metatarsal bone and calcaneus. Source data

To disentangle the skin–joint axis in psoriatic disease, we adapted the established PsA model of hydrodynamic *Il23* gene transfer in B10.RIII mice^4,15^ by introducing IL-23 overexpression (IL-23OE) into the more resistant inbred strains BALB/c and C57BL/6, aiming for a model system that is resistant to arthritis. Following IL-23OE, both strains developed characteristic psoriasis symptoms, including scaling and epidermal hyperplasia, within 3 days. These symptoms remained stable to a similar extent in both strains for up to 21 days (Fig. 1a and Extended Data Fig. 1a,d). Histological hematoxylin and eosin (H&E) staining and magnetic resonance imaging (MRI) revealed early signs of inflammation in the ankle joints of BALB/c mice on day 7, which progressed to inflammatory arthritis, including dactylitis, tendinitis, enthesitis and synovitis, by day 21. By contrast, C57BL/6 mice remained arthritis free (Figs. 1b,c and Extended Data Fig. 1b,c,e–g). Both strains exhibited systemic bone loss due to elevated osteoclast activation (Extended Data Fig. 1h,i)^15^, but osteoproliferative lesions were only found in animals with inflammatory arthritis (Extended Data Fig. 1j). Thus, we have established a model system that mimics the dichotomy of human psoriasis, allowing the identification of molecular differences associated with the spreading of inflammation from the skin to the joints.

**Extended Data Fig. 2:**
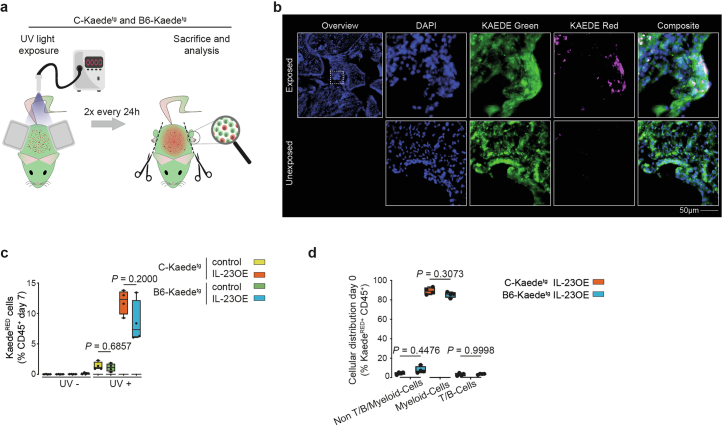
Validation of photoconversion. (**a**) Graphical cartoon explaining skin-photoconversion of the Kaede^tg^ mice upon ultraviolet (UV) exposure. (**b**) Representative micrographs of immunofluorescence microscopy of synovial tissue from hind paw joints of photoconverted (exposed) or not photoconverted (unexposed) Kaede^tg^ at day 7 with IL-23OE. (**c**) Quantification of Kaede^RED^ skin-derived cells in the joints at day 7. Graph shows median, quartiles and min-max; *N* = 4 per condition; *P* values were calculated by two-sided Mann-Whitney *U*-test. (**d**) Quantification of CD45^+^ Kaede^RED^ skin-derived cell types in the joints at steady state (day 0 of the IL-23OE model). Graph shows median, quartiles and min-max; *N* = 4 per condition; *P* values were calculated by one-way ANOVA with Tukey´s *post hoc* test. Source data

To follow the recirculation of skin-derived cells, we used photoconvertible Kaede^tg^ mice^16^ and light sheet microscopy to examine the ankle joints (Extended Data Fig. 2a). IL-23OE induced robust cell migration from the skin to the ankle joints, with the cells localizing to Kager’s fat pad and the synovium (Fig. 1d and Extended Data Fig. 2b). Unexpectedly, this migration was comparable in both the arthritis-developing BALB/c strain and the arthritis-resistant C57BL/6 strain.

We used flow cytometry of dissociated ankle tissues to quantify the migration of photoconverted skin-derived Kaede^RED^ cells over time. Migration was restricted to CD45^+^ leukocytes, increasing as early as day 4, peaking at day 7 and declining thereafter (Figs. 1e,f and Extended Data Fig. 2c). To investigate the nature of the skin-derived migrating Kaede^RED^ CD45^+^ leukocytes in more detail, we also used imaging cytometry. As expected, we found CD3^+^ T cells and B220^+^ B cells in the ankle joint, but also CD11b^+^ (CD3^−^B220^−^) myeloid cells disseminated from the skin (Fig. 1g). Unexpectedly, myeloid cells accounted for approximately 85% of the migrating cells in both strains (Fig. 1h and Extended Data Fig. 2d). The uniform cytosolic distribution of Kaede^GREEN^ and Kaede^RED^ protein also ruled out phagosomal scavenge transport of Kaede^RED^ protein among myeloid cells as being responsible for the Kaede^RED^ signal in the joint.

### Leukocyte trafficking along the skin–joint axis is dominated by myeloid cells

**Fig. 2:**
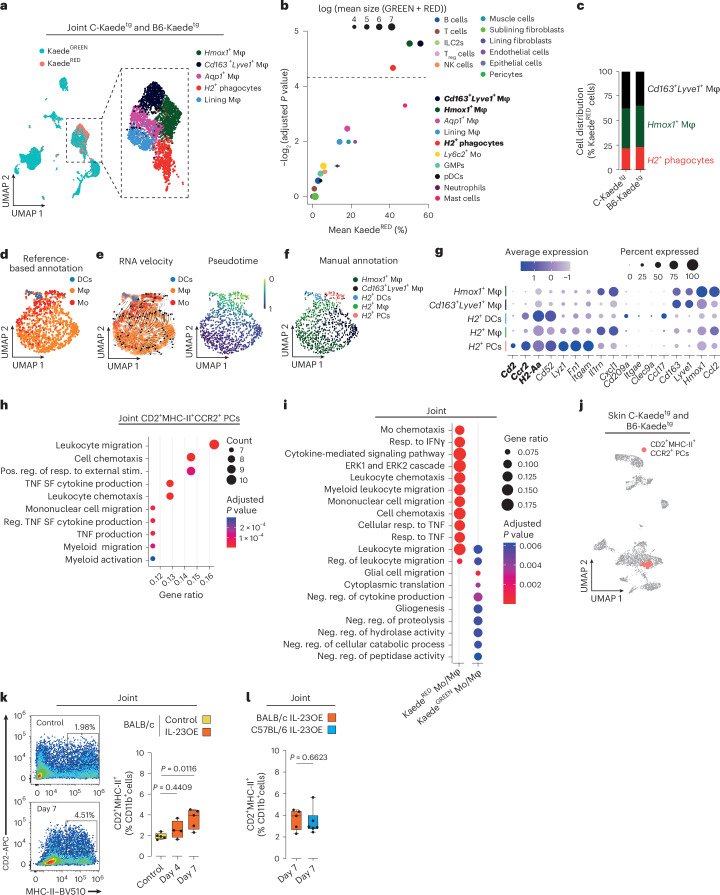
Skin-derived myeloid cells are precursors of mononuclear phagocytes in the joints. **a**, UMAP of hashtag-identified ankle joint cells in the scRNA-seq dataset from Kaede^tg^ mice on BALB/c (4,371 cells, *N* = 1 library from six pooled animals) and C57BL/6 (4,867 cells, *N* = 1 library from nine pooled animals) backgrounds on day 7 of the IL-23OE psoriasis model. Left, Kaede^GREEN^ and Kaede^RED^ cells are highlighted. Right, subcluster visualization of myeloid phagocytes (BALB/c 1,615 cells; C57BL/6 1,238 cells) with apparent highest abundance of Kaede^RED^ cells; Mφ, macrophages. **b**, Scatter plot corresponding to **a** showing the log transferred cell abundance and the Kaede^RED^ percentage in each of the identified clusters versus the Benjamini and Hochberg (BH)-adjusted *P* values calculated using quasibinomial models. The dashed line indicates the significance threshold (BH-adjusted *P* value of <0.05); T_reg_ cells, regulatory T cells; NK, natural killer; Mo, monocytes; GMP, granulocyte–monocyte progenitors; pDCs, plasmacytoid DCs. **c**, Bar plot comparing the ratio of clusters of myeloid cells to the highest Kaede^RED^ percentage between strains (BALB/c, 1,023 cells; C57BL/6, 819 cells). **d**, Reference-based annotation of each single cell of the Kaede^RED^ myeloid cells (BALB/c, 498 cells; C57BL/6, 407 cells) based on the ImmGen mononuclear phagocyte dataset (GSE122108) identified by singleR. **e**, Velocity streams (left) and pseudotime (right) identified by CellRank of the Kaede^RED^ myeloid cells visualized over the UMAP. **f**, UMAP visualization of Kaede^RED^ myeloid cells with subclusters of *H2*^+^ phagocytes after manual annotation; PCs, precursors. **g**, Expression of the most relevant marker genes among each myeloid cluster from **f**. **h**, Significantly enriched GO-BP terms in the CD2^+^MHC-II^+^CCR2^+^ myeloid precursor cluster compared to all other cell types in the joint. The selection criteria included a BH-adjusted *P* value of <0.05; Pos., positive; reg., regulation; stim., stimulus; SF, superfamily. **i**, Comparison of significantly enriched GO-BP terms between Kaede^RED^ and Kaede^GREEN^ myeloid cells. The selection criteria included a BH-adjusted *P* value of <0.05; Resp. response; IFNγ, interferon-γ; Neg., negative. **j**, Identified CD2^+^MHC-II^+^CCR2^+^ myeloid precursors using UCell highlighted on the UMAP plot of the hashtag-identified skin leukocytes in the scRNA-seq dataset from Kaede^tg^ animals with BALB/c (2,494 cells, *N* = 1) and C57BL/6 (3,024 cells, *N* = 1) background strains on day 7 of IL-23OE. **k**, Representative flow cytometry plots for the quantification of CD2^+^MHC-II^+^CCR2^+^ skin-derived myeloid precursors in the joint (left) and their quantification in BALB/c mice over time (right). The graph shows the median, quartiles and minimum–maximum; *N* = 5, *N* = 4 and *N* = 5 per time point and condition. *P* values were calculated by one-way ANOVA with a Tukey’s post hoc test. **l**, Quantification of CD2^+^MHC-II^+^CCR2^+^ skin-derived myeloid precursors in the joints of BALB/c and C57BL/6 mice on day 7. The graph shows the median, quartiles and minimum–maximum; *N* = 5 and *N* = 6 per time point and condition. *P* values were calculated by two-sided Mann–Whitney *U*-test. Source data

**Extended Data Fig. 3:**
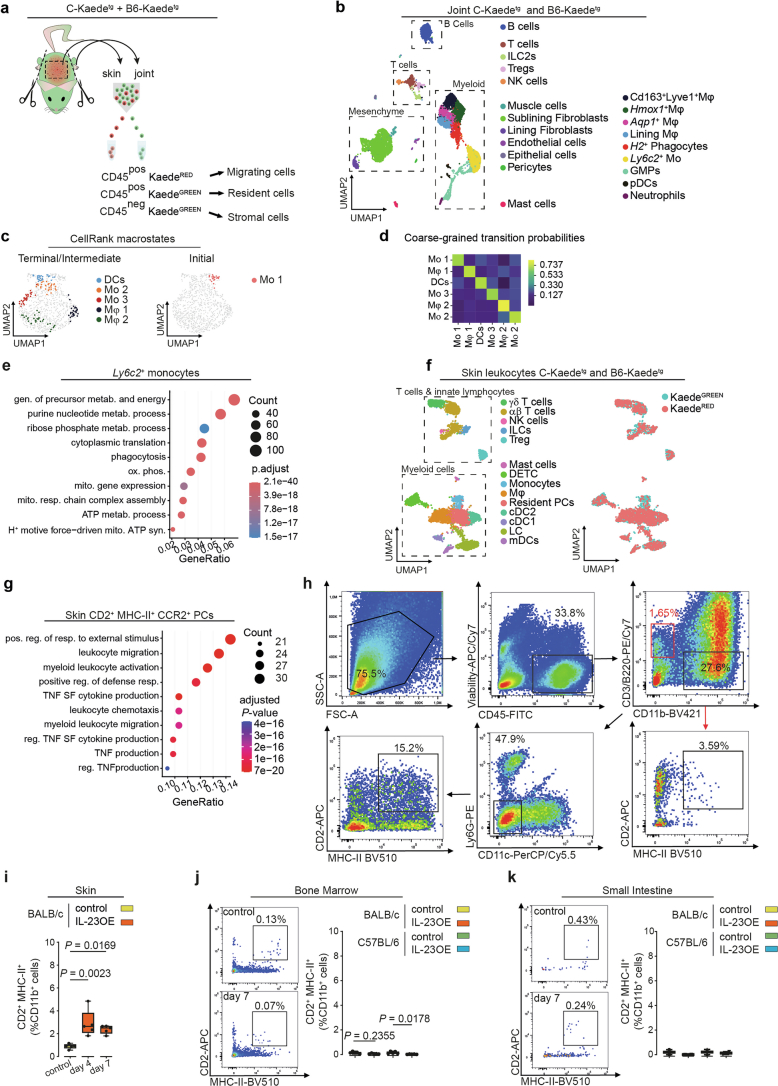
Mouse single-cell landscape in joints and skin. **(a)** Graphical cartoon showing the strategy to cell sort target populations used for scRNA-seq. Target populations were individually hash-tagged before generating the scRNA-seq library. **(b)** UMAP plot of the hash-tag identified ankle joint cells in the scRNA-seq dataset from Kaede^tg^ mice (BALB/c and C57BL/6) on day 7 of the IL-23OE model. Refers to Fig. 2**a**. Detailed annotation with Seurat-identified clusters shown. **(c)** CellRank identification of terminal (left) and initial states (right) in Kaede^RED^ myeloid cells. **(d)** Coarse-grained matrix of transition probabilities between states calculated by CellRank. **(e)** Significantly enriched GO-BP terms in Ly6c2^+^ monocyte cluster, when compared to all other cell types in the joint. Selection criteria: BH-adjusted *P* value < 0.05. **(f)** UMAP plot of the hash-tag identified skin leukocytes in the scRNA-seq dataset from Kaede^tg^ mice (BALB/c 2,494 cells, *N* = 1 and C57BL/6 3,024 cells, *N* = 1) on day 7 of the IL-23OE model. Annotated Seurat identified clusters shown (left). Kaede^GREEN^ and Kaede^RED^ cells are highlighted (right). **(g)** Significantly enriched GO-BP terms in skin CD2^+^MHC-II^+^CCR2^+^ myeloid precursors (PCs) cluster, when compared to other cell types. Selection criteria: BH-adjusted *P* value < 0.05. **(h)** Representative flow cytometry plots showing the gating strategy for the identification of CD2^+^MHC-II^+^CCR2^+^ skin-derived PCs in the joint. Red gate on T/B lymphocytes as internal control for CD2 and MHC-II. **(i)** Quantification of CD2^+^MHC-II^+^ myeloid cells in the skin of BALB/c animals after IL-23OE over time. Graph shows median, quartiles, min-max.; *N* = 5, per timepoint and condition; *P*-values calculated by one-way ANOVA with Tukey´s post hoc test. **(j, k)** Representative flow cytometry plots showing the gating of CD2^+^MHC-II^+^ skin-derived myeloid PCs in the bone marrow (**j**, left) or small intestine (**k**, left) and their respective quantification after IL-23OE on day 7 in BALB/c and C57BL/6 mice (**j, k** right). Graph **(j)** shows median, quartiles, min-max.; *N* = 5, per condition; *P* values calculated by one-way ANOVA with Tukey’s post hoc test. Graph **(k)** shows median, min-max.; *N* = 2, per condition; no *P* values calculated. Source data

Having observed that cellular migration from psoriatic skin did not predict the subsequent development of arthritis, we aimed to profile skin-derived and joint-invading leukocytes in detail. We generated a comprehensive single-cell RNA-sequencing (scRNA-seq) dataset of skin- and joint-resident Kaede^GREEN^ leukocytes (CD45^+^) and stromal cells (CD45^−^) and Kaede^RED^ migrated leukocytes (CD45^+^; Extended Data Fig. 3a). Using uniform manifold approximation and projection (UMAP) for visualization, we confirmed that most Kaede^RED^ migrated leukocytes in the joint belonged to the myeloid phagocyte cluster (Fig. 2a and Extended Data Fig. 3b). Subsequent clustering identified five different subclusters consistent with previous reports^17–20^, namely MHC-II^−^ lining layer macrophages, *Hmox1*^+^ macrophages, *Cd163*^+^*Lyve1*^+^ anti-inflammatory macrophages, *Aqp1*^+^ macrophages and MHC-II^+^ (*H2*^*+*^) phagocytes. Three of the five subclusters, namely *Hmox1*^+^ macrophages, *Cd163*^+^*Lyve1*^+^ anti-inflammatory macrophages and *H2*^+^ phagocytes, were significantly enriched in Kaede^RED^ migrated cells (Fig. 2b). However, their abundance did not differ between the two mouse strains, confirming that migration alone is not sufficient to drive the spread of inflammation from the skin to the joints (Fig. 2c).

### CD2^+^MHC-II^+^CCR2^+^ myeloid precursors are skin-derived joint-invading cells

To better characterize the Kaede^RED^ myeloid compartment, we performed a reference-based annotation using the Immunological Genome Project mononuclear phagocytes dataset (GSE122108)^21,22^. This clustering-independent annotation identified monocytes, macrophages and dendritic cells (DCs; Fig. 2d). We then examined the transcriptional dynamics and differentiation potential of Kaede^RED^ phagocytes using CellRank^23^, combining transcriptional turnover, as estimated by RNA velocity, with transcriptional similarity. CellRank identified several macro states among these cells but identified *H2*^+^ precursors as the only initial state with the potential to differentiate into *H2*^+^ phagocytes and *Hmox1*^+^ and *Cd163*^+^*Lyve1*^+^ macrophages, perfectly aligning with CellRank’s assisted pseudotime estimation (Fig. 2e and Extended Data Fig. 3c,d). We therefore subclustered *H2*^+^ phagocytes into *H2*^+^ DCs, *H2*^+^ macrophages and *H2*^+^ myeloid precursors (Fig. 2f), which also precisely overlapped with the reference-based annotation. Among the *H2*^+^ phagocytes, *H2*^+^ myeloid precursors could be distinguished by the expression of *Cd2* and *Ccr2* (Fig. 2g). We will therefore refer to them as CD2^+^MHC-II^+^CCR2^+^ myeloid precursors. Consistent with our findings, Gene Ontology Biological Process (GO-BP) enrichment analysis revealed a significant contribution of migration-associated pathways when comparing CD2^+^MHC-II^+^CCR2^+^ myeloid precursors to other subsets in the joints (Fig. 2h). Similarly, migrated Kaede^RED^ phagocytes exhibited a higher abundance of chemotaxis- and migration-related terms than the resident Kaede^GREEN^ population (Fig. 2i). We also identified a pure Kaede^GREEN^ cluster of potential precursors (Extended Data Fig. 3b), *Ly6c2*^+^ monocytes^17^, which were characterized by high metabolic activity based on GO-BP enrichment analysis (Extended Data Fig. 3e).

Based on the CellRank and GO-BP enrichment analyses, we hypothesized that a CD2^+^MHC-II^+^CCR2^+^ myeloid precursor population originated in the skin. Using the transcriptional profile of joint CD2^+^MHC-II^+^CCR2^+^ myeloid precursors, we scored the skin myeloid compartment by UCell^24^ and identified these myeloid precursors among monocytes in the skin (Fig. 2j and Extended Data Fig. 3f). CD2^+^MHC-II^+^CCR2^+^ myeloid precursors in the skin showed a significant enrichment of GO-BP terms associated with cellular migration (Extended Data Fig. 3g).

To confirm the cellular trafficking of CD2^+^MHC-II^+^CCR2^+^ myeloid precursors between the skin and joints independently of the Kaede^tg^ system, we developed a flow cytometry strategy to detect CD2^+^ cells among CD11b^+^MHC-II^+^ cells in the joints and skin (Extended Data Fig. 3h). Compared to untreated mice, IL-23OE induced a significant expansion of CD2^+^MHC-II^+^ myeloid precursors in the skin and joints of BALB/c mice on days 4 and 7 (Fig. 2k and Extended Data Fig. 3i). Notably, no difference was observed between BALB/c and C57BL/6 inbred strains (Fig. 2l). No significant increase in CD2^+^MHC-II^+^ myeloid precursors was observed in the bone marrow or the small intestine following IL-23OE (Extended Data Fig. 3j,k). As both organs are affected by IL-23OE^15,25^, this finding supports the concept that the skin is the source of these cells in the joint.

### Leukocyte trafficking is conserved between mice and humans

**Fig. 3:**
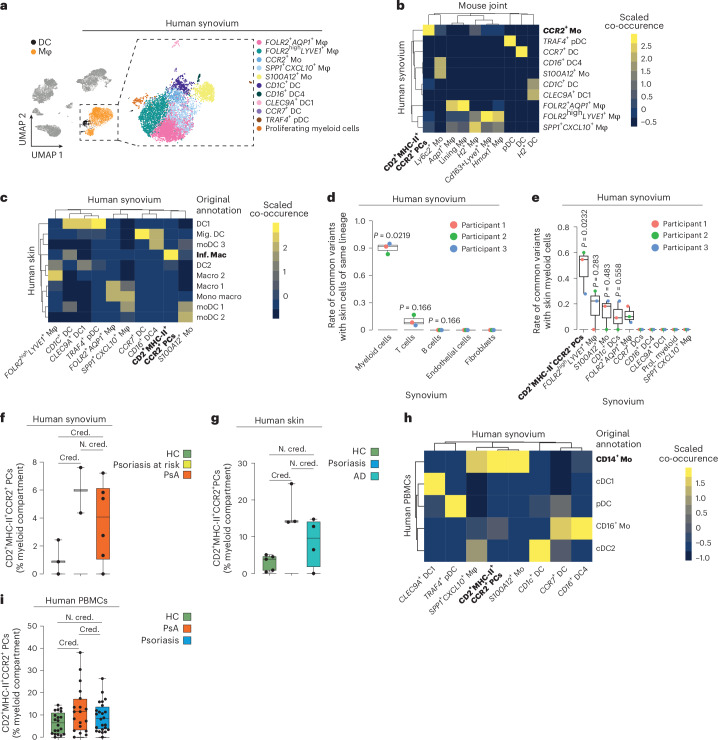
Evidence for a skin–joint axis in humans. **a**, UMAP plot of the scVI-integrated scRNA-seq datasets of human PsA synovium (*N* = 5; E-MTAB-11791) and healthy synovial tissue (*N* = 3). Identified clusters of myeloid cells are highlighted. **b**, Heat map of co-occurrence of psoriatic mouse myeloid cell clusters with human PsA myeloid cell clusters when mouse joint annotations were mapped to human cells using scANVI. **c**, Heat map of co-occurrence of human synovial myeloid cell clusters (PsA *N* = 5, healthy *N* = 3) with skin myeloid cell clusters from a human skin scRNA-seq dataset (healthy participants *N* = 5, participants with psoriasis *N* = 3, participants with AD *N* = 4; E-MTAB-8142) when synovial annotations were mapped to skin annotations (retrieved from metadata) using scANVI; Mig., migrating; moDC, monocyte-derived DC 3; Inf. Mac, inflammatory macrophages; Macro, macrophages; Mono macro, monocyte-derived macrophages. **d**, Proportions of shared mitochondrial variants between synovial and skin clusters of the same lineage for the major clusters in the synovia. The graph shows the median, quartiles and minimum–maximum; *N* = 3. *P* values were calculated by one-versus-rest two-sided Wilcoxon rank-sum test with a BH correction. **e**, Box plot corresponding to **d**. The synovial myeloid compartment is shown in detail; Prol., proliferating. **f**, Proportion of CD2^+^MHC-II^+^CCR2^+^ myeloid precursors in psoriasis at risk and PsA compared to healthy in the scRNA-seq dataset of human synovial tissue. The graph shows the median, quartiles (for *N* > 3) and minimum–maximum; psoriasis at risk *N* = 2; PsA *N* = 5; healthy participants (HC) *N* = 3. Statistically credible (Cred.) and noncredible (N. cred.) changes in abundance were identified using scCODA. **g**, Proportion of CD2^+^MHC-II^+^CCR2^+^ myeloid precursors in AD and psoriasis compared to healthy in the scRNA-seq dataset of human skin. The graph shows the median, quartiles and minimum–maximum; AD *N* = 4; psoriasis *N* = 3; healthy *N* = 5. Statistically credible and noncredible changes in abundance were identified using scCODA. **h**, Heat map of co-occurrence of human myeloid cell clusters from the joint (PsA *N* = 5, healthy participants *N* = 3) with the PBMC myeloid cell cluster from the proteogenomic dataset (healthy participants *N* = 20, PsA *N* = 19, psoriasis *N* = 24; GSE194315) when joint annotations were mapped to preannotated PBMCs using scANVI. **i**, Proportion of CD2^+^MHC-II^+^CCR2^+^ myeloid precursors in psoriasis and PsA compared to healthy in the scRNA-seq dataset of human PBMCs. The graph shows the median, quartiles and minimum–maximum; psoriasis *N* = 24; PsA *N* = 19; healthy *N* = 20. Statistically credible and noncredible changes in abundance were identified using scCODA. Source data

**Extended Data Fig. 4:**
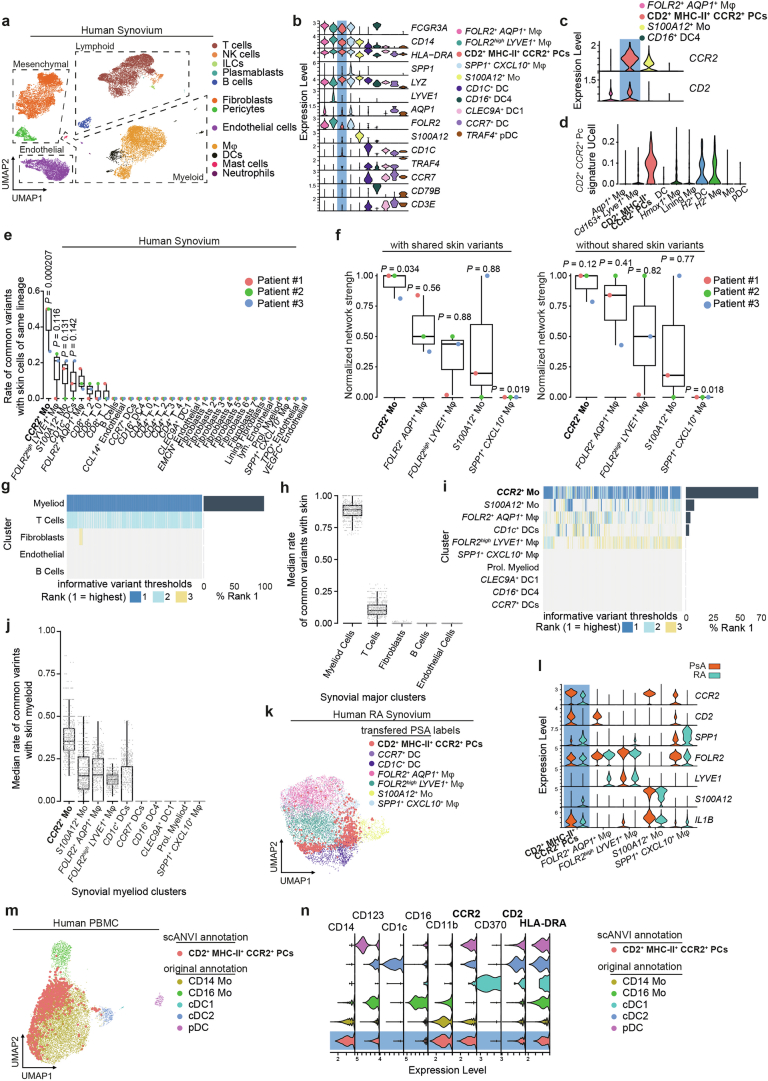
Human myeloid compartment in synovium, skin and PBMC of psoriatic disease. (**a**) UMAP plot of the identified clusters of major cell types in the scVI-integrated scRNA-seq datasets of human (PsA, *N* = 5, E-MTAB-11791) and healthy synovial tissue (*N* = 3). **(b)** Violin plots of relevant marker genes based on ALRA-imputed gene expression among the identified clusters of human synovial myeloid cells from Fig. 3a. Abbreviation: precursors (PCs). **(c)** Violin plots of ALRA-imputed gene expression of selectively CD2 and CCR2 among the identified clusters of human synovial myeloid cells from Fig. 3a. **(d)** Comparison of the UCell score of the gene signature for human joint CD2^+^MHC-II^+^CCR2^+^ myeloid precursors in the myeloid phagocytes of the scRNA-seq dataset of mouse ankle joint among dataset from Fig. 2a. **(e)** Proportions of shared mitochondrial variants between synovial and skin clusters of the same lineage for all myeloid, T and B cell, fibroblast and endothelial clusters in the synovia. Graph shows median, quartiles, min-max.; *N* = 3; *P* values calculated by one-vs-rest two-sided Wilcoxon rank-sum test with BH correction. **(f)** Normalized network strength for the shared mitochondrial variants among subclusters of myeloid cells in the synovia, when all shared variants are included (left) or shared variants with skin are excluded (right). Graph shows median, quartiles, min-max.; *N* = 3; *P*-values calculated by one-versus-rest two-sided Wilcoxon rank-sum test with BH correction. **(g-j)** Median rank (*N* = 3) in each iteration of synovia-skin shared variant identification among major cell clusters **(g-h)** and among myeloid cell clusters **(i-j)** shown as heatmap **(g, i)** or boxplot **(h, j)**. Graphs show median, quartiles, min-max. **(k)** UMAP plot of a scRNA-seq dataset of synovial myeloid cells of treatment naive individuals with rheumatoid arthritis (RA, *N* = 5, E-MTAB-8322). **(l)** Violin plots of the ALRA-imputed signature genes for each cluster identified in the RA and PsA synovial myeloid cell scRNA-seq datasets. **(m)** UMAP plot of integrated CITEseq dataset of human PBMC myeloid cells. Publication annotations along with scANVI identified clusters of CD2^+^MHC-II^+^CCR2^+^ myeloid precursors shown. **(n)** Expression levels of most relevant CITEseq antibodies among publication clusters of human PBMC dataset along with scANVI identified clusters of CD2^+^MHC-II^+^CCR2^+^ myeloid precursors. Source data

We next asked whether similar migration mechanisms exist in humans by integrating scRNA-seq datasets from synovial tissues (healthy *N* = 3, PsA *N* = 5; E-MTAB-11791 (ref. ^26^)). Myeloid cells were subclustered into 11 distinct cellular identities by unsupervised clustering and labeled according to their gene expression profile (Fig. 3a and Extended Data Fig. 4a,b). Using single-cell annotation using variational inference (scANVI)^27^ integration and reference mapping, which showed optimal results in a benchmarking comparison^28^, we aimed to find biologically relevant human–mouse orthologous populations within the myeloid compartment (Fig. 3a). In addition to mouse and human DC subsets, we observed the highest degree of co-occurrence between mouse CD2^+^MHC-II^+^CCR2^+^ myeloid precursors and human *CCR2*^+^ monocyte subclusters (Fig. 3b). Human *CCR2*^+^ monocytes expressed *CD2* and *HLA-DR*, in agreement with mouse CD2^+^MHC-II^+^CCR2^+^ myeloid precursors (Extended Data Fig. 4b,c). Reverse detection of human myeloid precursor signatures among mouse myeloid cells using UCell confirmed the high degree of similarity between human and mouse CD2^+^MHC-II^+^CCR2^+^ myeloid precursors (Extended Data Fig. 4d).

To trace CD2^+^MHC-II^+^CCR2^+^ myeloid precursors to the skin, we integrated the human synovial myeloid cell clusters into an existing scRNA-seq dataset of skin myeloid cells (E-MTAB-8142)^29^ containing samples from healthy donors (*N* = 5), individuals with psoriasis (*N* = 3) and individuals with atopic dermatitis (AD; *N* = 4). Mapping synovial myeloid signatures to the preannotated dermal myeloid cells using scANVI transfer revealed a high degree of co-occurrence between synovial CD2^+^MHC-II^+^CCR2^+^ myeloid precursors and dermal myeloid cells that were originally termed ‘inflammatory macrophages’ (Fig. 3c)^29^.

Having observed orthologous populations of human and mouse CD2^+^MHC-II^+^CCR2^+^ myeloid precursors in the skin and synovium, we sought to track their migration between these two tissues in humans. We hypothesized that cells migrating from the skin to the joint would exhibit a significantly higher prevalence of shared somatic mitochondrial mutations than either resident cells or cells originating from distinct sources within both organs. We generated three matched scRNA-seq datasets of skin and joint tissue from three individuals (Supplementary Fig. 1a–d; early PsA *N* = 1, psoriasis at risk of developing PsA *N* = 2). Additionally, we enriched and sequenced cDNA specifically for mitochondrial transcripts to detect mitochondrial somatic mutations using the mitochondrial alteration enrichment from single-cell transcriptomes to establish relatedness (MAESTER) method^30^, which enabled us to establish these lineage relationships. Using conservative quality and specificity thresholds for informative variant detection, we identified a median of 12 (interquartile range = 3.5) shared variants between the skin and synovium. Most of these variants were found in myeloid cells, with a smaller number found in T cells (Fig. 3d). Notably, CD2^+^MHC-II^+^CCR2^+^ myeloid precursors exhibited the highest enrichment of conserved somatic mitochondrial mutations (Fig. 3e). We considered the possibility that the methodology might have favored myeloid cells, given their presumably shorter lifespan and recent origin from bone marrow precursors, as this could result in greater preservation of somatic variants. To exclude this possibility, we analyzed *S100A12*⁺ monocytes infiltrating the synovia^18^ and found that they had fewer shared variants than CD2^+^MHC-II^+^CCR2^+^ myeloid precursors, comparable to T cells (Extended Data Fig. 4e). Ranking the synovial myeloid clusters based on the extent of shared variants among them, both including and excluding those shared with skin, using a graph-based lineage tracing (Supplementary Fig. 1e) identified CD2⁺MHC-II⁺CCR2⁺ myeloid cells as the earliest precursors provided that the conserved skin variants were included (Extended Data Fig. 4f). This analysis also confirmed that the shared variants in the other joint myeloid clusters were derived from CD2^+^MHC-II^+^CCR2^+^ myeloid precursors, ruling out other migratory sources. To exclude the possibility that our selection criteria for informative variants had affected the results, we performed an iterative analysis, defining variable ranges for each selection criterion. As expected, myeloid cells consistently exhibited the highest number of shared variants with skin cells in 100% of the iterations (Extended Data Fig. 4g,h). Within the myeloid clusters, CD2^+^MHC-II^+^CCR2^+^ myeloid precursors were consistently ranked first in approximately 70% of iterations (Extended Data Fig. 4i,j). Together, these results demonstrate the presence of a human ortholog of CD2^+^MHC-II^+^CCR2^+^ myeloid precursors and their conserved migration between the skin and synovium.

### Disease-specific expansion of myeloid precursors

We explored whether disease context influences the number of CD2^+^MHC-II^+^CCR2^+^ myeloid precursors in affected organs in humans. Consistent with our observation in mice, we observed that CD2^+^MHC-II^+^CCR2^+^ myeloid precursors were significantly elevated in the synovial tissues of individuals with PsA compared to in healthy control individuals. This increase was evident even in individuals with psoriasis at risk of developing PsA (Fig. 3f and Supplementary Fig. 2a). By contrast, synovial myeloid cells from treatment-naive individuals with rheumatoid arthritis (*N* = 5, E-MTAB-8322 (ref. ^18^); Extended Data Fig. 4k) did not exhibit any *CD2* expression in precursor cells (Extended Data Fig. 4l). This highlights the unique disease-specific and skin-specific signature of CD2^+^MHC-II^+^CCR2^+^ myeloid precursors.

In human skin, CD2^+^MHC-II^+^CCR2^+^ myeloid precursors were significantly more prevalent in individuals with psoriasis than in healthy control individuals (Fig. 3g and Supplementary Fig. 2b–d). As a proof of concept, no significant increase in CD2^+^MHC-II^+^CCR2^+^ myeloid precursors was observed in AD, which has an etiology different from that of psoriatic disease (Supplementary Fig. 2b,d). These findings confirmed the co-occurrence of CD2^+^MHC-II^+^CCR2^+^ myeloid precursors in skin and synovial tissue, which increased under pathological conditions, consistent with our observations in mice.

Finally, to support the hypothesis of their migration along the skin–joint axis, we tested whether skin-disseminating recirculating precursors were detectable in the human peripheral blood mononuclear cell (PBMC) fraction. We integrated the scRNA-seq datasets of myeloid cells from synovial tissues with an existing dataset of PBMCs (GSE194315)^31^ from healthy donors (*N* = 20) and individuals with psoriatic disease (psoriasis: *N* = 24; PsA: *N* = 19). In addition to the gene expression data, GSE194315 partially included proteogenomic data that allowed differentiation of cells based on antibody-stained surface markers. Transferring the synovial annotations to the proteogenomic-based preannotation of PBMCs using scANVI revealed a high degree of co-occurrence between synovial CD2^+^MHC-II^+^CCR2^+^ myeloid precursors and CD14^+^ blood monocytes (Fig. 3h). The mapped synovial CD2^+^MHC-II^+^CCR2^+^ myeloid precursors formed a subcluster among CD14^+^ blood monocytes, revealing as a distinct population of antibody-identifiable CD2^+^MHC-II^+^CCR2^+^ myeloid precursors (Extended Data Fig. 4m,n). Comparing the abundance of myeloid populations between the different conditions showed that CD2^+^MHC-II^+^CCR2^+^ myeloid precursors were increased in the blood of individuals with PsA (Fig. 3i and Supplementary Fig. 2e,f). In conclusion, we have provided evidence that CD2^+^MHC-II^+^CCR2^+^ myeloid precursors increase not only in the circulation but also in the skin and synovial tissue of individuals with psoriatic disease, fully mirroring the observations made in the animal models.

### Joint fibroblasts affect activation of skin-derived mononuclear phagocytes

**Fig. 4:**
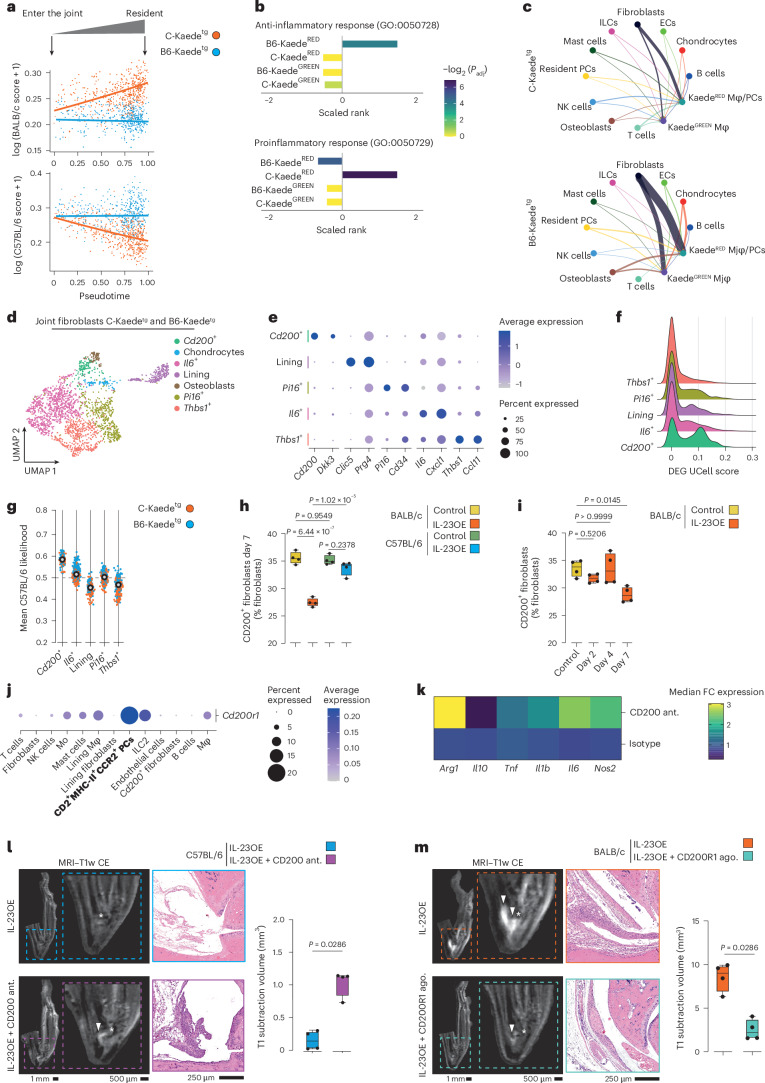
Joint fibroblasts prime migrating precursors to proinflammatory or anti-inflammatory phenotypes. **a**, TradeSeq-fitted smooth expression of UCell scores of genes dynamically associated with either BALB/c (top) or C57BL/6 (bottom) mice over pseudotime from Fig. 2e in the scRNA-seq dataset of ankle joints from Kaede^tg^ mice (BALB/c and C57BL/6 strains) on day 7 of the IL-23OE model; *P*_adj_, adjusted *P* value. **b**, UCell scores for GO-BP terms GO:0050728 (anti-inflammatory response) and GO:0050729 (proinflammatory response) on the phagocyte compartment from Fig. 2a stratified by stain and photoconversion status. *P* values were calculated with a two-sided Wilcoxon signed-rank test and adjusted for multiple comparisons using the BH method. **c**, Circle plot showing the communication probability between myeloid cells (Kaede^GREEN^ or Kaede^RED^) and other cell types in the joints associated with either BALB/c (top) or C57BL/6 (bottom) mice. Line thickness corresponds to communication probability determined using CellChat; ECs, endothelial cells; Mφ, macrophage. **d**, UMAP plot of Seurat-identified subclusters among fibroblasts in the scRNA-seq dataset of ankle joints from Kaede^tg^ mice (BALB/c and C57BL/6 strains) on day 7 of the IL-23OE model. **e**, Expression of the most relevant marker genes among each fibroblast cluster identified in **d**. **f**, Comparison of UCell scores of the C57BL/6-associated gene signature (fibroblast-only differentially expressed genes in C57BL/6 mice compared to BALB/c mice) between different subclusters of joint fibroblasts; DEG, differentially expressed gene. **g**, C57BL/6-associated relative likelihood of different subclusters of fibroblasts determined by MELD. **h**, Quantification of CD200^+^ fibroblasts in the joints of BALB/c and C57BL/6 mice on day 7. The graph shows the median, quartiles and minimum–maximum; *N* = 4 per time point and condition. *P* values were calculated by one-way ANOVA with a Tukey’s post hoc test. **i**, Quantification of CD200^+^ fibroblasts in the joints of BALB/c mice on days 2, 4 and 7. The graph shows the median, quartiles and minimum–maximum; *N* = 4 per time point and condition. *P* values were calculated by one-way ANOVA with a Tukey’s post hoc test. **j**, Expression of *Cd200r1* among the different cell clusters in the scRNA-seq dataset of ankle joints from Kaede^tg^ mice (BALB/c and C57BL/6 strains) on day 7 of the IL-23OE model. **k**, Heat map of median fold change gene expression in macrophage–fibroblast cocultures treated with anti-CD200 antagonist (ant.; OX-90) or isotype control; *N* = 7; FC, fold change. **l**, Representative images of MRI scans and micrographs of H&E-stained ankles of C57BL/6 animals at day 21 after IL-23OE with or without anti-CD200 antagonist (OX-90) treatment. MRI quantification of arthritis at day 21 is shown on the right. Arrowheads indicate inflammation, and stars indicate the talar bone. The graph shows the median, quartiles and minimum–maximum; *N* = 4 per condition. *P* values were calculated by two-sided Mann–Whitney *U*-test. **m**, Representative images of MRI scans and micrographs of H&E-stained ankles of BALB/c animals at day 21 after IL-23OE with or without anti-CD200R1 agonist (ago.; OX-110) treatment. MRI quantification of arthritis at day 21 is shown on the right. Arrowheads indicate inflammation, and stars indicate the talar bone. The graph shows the median, quartiles and minimum–maximum; *N* = 4 per condition. *P* values were calculated by two-sided Mann–Whitney *U*-test. Source data

**Extended Data Fig. 5:**
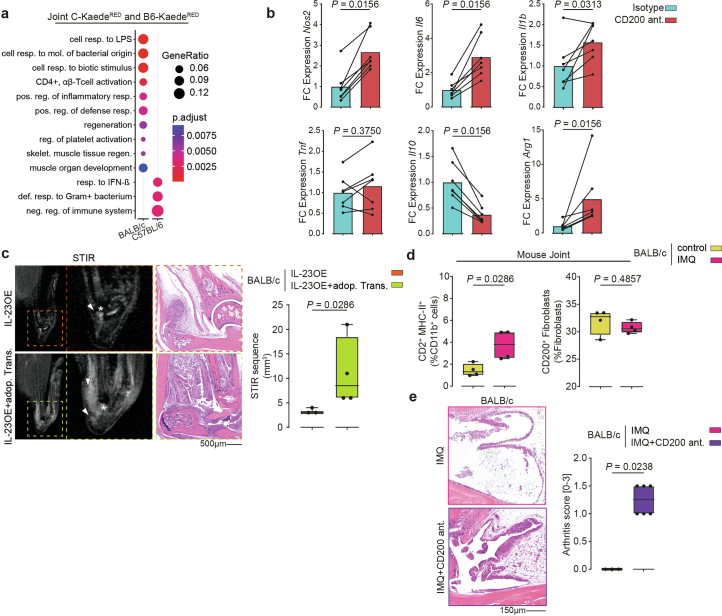
Pro-inflammatory responses in BALB/c ankle joints. (**a**) Comparison of the significantly enriched GO-BP terms in the genes dynamically associated with either BALB/c or C57BL/6 strain over pseudo-time as shown in Fig. 4a. Selection criteria: BH-adjusted *P* value < 0.05. (**b**) Mean fold change (FC) gene expression in macrophage-fibroblasts co-cultures treated with CD200 antagonist (OX-90) or isotype; *N* = 7 per condition; *P* values were calculated by two-sided Wilcoxon matched-pairs signed-rank test. (**c**) Representative images of MRI-scans and H&E-stained ankles of BALB/c mice at day 14 after IL-23OE with or without adoptive transfer of skin-derived myeloid cells at day 7 with IL-23OE. MRI-quantification of arthritis at day 21. Arrowheads indicate inflammation. Stars indicate the talar bone. Graph shows median, quartiles and min-max; *N* = 4 per condition; *P* values were calculated by Mann-Whitney *U*-test (**d**) Quantification of CD2^+^MHC-II^+^CCR2^+^ skin-derived myeloid precursors and of CD200^+^ fibroblasts in the joint of BALB/c on day 7 with or without imiquimod (IMQ). Graph shows median, quartiles and min-max.; *N* = 4 per condition; *P* values were calculated by Mann-Whitney *U*-test. (**e**) Representative micrographs of H&E-stained ankles of BALB/c mice on day 21 after IMQ with or without anti-CD200 antagonist (OX-90) treatment and corresponding quantification of arthritis score; *N* = 3 mice treated with IMQ and *N* = 6 mice with IMQ and CD200 antagonist; *P* values were calculated by Mann-Whitney *U*-test. Mann-Whitney *U*-tests in (**c**), (**d**) and (**e**) were all two-sided. Source data

As CD2^+^MHC-II^+^CCR2^+^ myeloid precursors are a rather dynamic population that differentiate into several subpopulations after their arrival in the joints, we wondered whether the local tissue microenvironment influences this process. Using trajectory-based differential expression analysis for sequencing data (tradeSeq)^32^, we compared the change in gene expression profiles of CD2^+^MHC-II^+^CCR2^+^ myeloid precursors between BALB/c and C57BL/6 mice over pseudotime. Minimal changes were observed in arthritis-resistant C57BL/6 mice, whereas BALB/c mice showed a much greater divergence from baseline (Fig. 4a). GO-BP analysis of differentially dynamic genes revealed several proinflammatory pathways in BALB/c mice compared to C57BL/6 mice, resistant to arthritis (Extended Data Fig. 5a). Correspondingly, scoring myeloid phagocyte clusters (Fig. 2a) with UCell for pro- and anti-inflammatory responses revealed a higher proinflammatory response in Kaede^RED^ cells from BALB/c mice, but a higher anti-inflammatory response in Kaede^RED^ cells from C57BL/6 mice (Fig. 4b). The Kaede^GREEN^ compartment, consisting mainly of lining layer and *Aqp1*^+^ macrophages, showed no significant anti- or proinflammatory activation in either background strain. These analyses first demonstrate that skin-derived Kaede^RED^ cells are the key regulators of inflammation and, second, that the joint microenvironment plays a key role in priming this compartment for pro- or anti-inflammatory activation.

Therefore, we used CellChat^33^ to explore the interactions between migrated precursors and their local microenvironment, which revealed robust communication probabilities between Kaede^RED^ phagocytes and the microenvironment in both strains. The overall communication strength of Kaede^RED^ migrated cells was higher than that of Kaede^GREEN^ resident cells (Fig. 4c). The strongest communication probabilities were found between Kaede^RED^ phagocytes and fibroblasts, and the interaction was predicted to be stronger in C57BL/6 mice than in BALB/c mice. To disentangle putative interaction partners, we subclustered the fibroblast compartment in the joints according to previous reports (Figs. 4d,e)^34–37^. We identified synovial lining and sublining fibroblasts, with the latter being subclustered into *Thbs1*^+^, *Il6*^+^, *Cd200*^+^ and *Pi16*^+^ fibroblasts, as well as chondrocytes and osteoblasts. Having observed a high interaction probability between fibroblasts and Kaede^RED^ phagocytes, we used UCell to score fibroblast-specific differentially expressed genes in C57BL/6 mice compared to BALB/c mice. *Cd200*^+^ fibroblasts displayed the highest score (Fig. 4f), arguing that this population was the critical determinant in the microenvironment. MELD^38^ analysis showed that *Cd200*^+^ fibroblasts were more likely to be found in C57BL/6 mice than in BALB/c mice (Fig. 4g). Finally, we used flow cytometry to quantify the abundance of CD200^+^ fibroblasts (CD45^−^CD31^−^PDPN^+^PDGFRα^+^CD200^+^THY1^+^CD49f^−^) in joints before and after IL-23OE on day 7 (ref. ^34^). Consistent with the scRNA-seq dataset, we observed a significant decrease in CD200^+^ fibroblasts after IL-23OE in BALB/c, but not in C57BL/6, mice (Fig. 4h). This decrease coincided with peak migration (Fig. 4i). Collectively these findings suggest that arthritis is controlled not only by the migration of CD2^+^MHC-II^+^CCR2^+^ myeloid precursors from the skin to the joint but also by CD200^+^ fibroblasts in the joint.

### The CD200–CD200R1 axis controls arthritis onset

CD200 is known to interact with four different receptors^39^, of which the gene encoding CD200R1 was the most abundantly expressed in our mouse scRNA-seq dataset. Consistent with our previous report^34^, we found *Cd200r1* transcripts in a few T cells and strong expression on type 2 innate lymphoid cells (ILC2s) and myeloid cells (Fig. 4j). In particular, the high cellular resolution of our dataset in myeloid cells allowed us to analyze the expression in this compartment in more detail, and we observed the highest abundance of *Cd200r1* transcripts in CD2^+^MHC-II^+^CCR2^+^ myeloid precursors. There was no significant difference in expression between the background strains. Functionally, when we cultured bone marrow monocytes with synovial fibroblasts and blocked the CD200–CD200R1 interaction with a monoclonal antibody to CD200 (OX-90), we observed the induction of a proinflammatory phenotype in bone marrow monocytes with higher expression of *Il1b*, *Il6* and *Tnf* (Fig. 4k and Extended Data Fig. 5b). These findings suggest that CD200^+^ fibroblasts form a protective barrier in the joint through the CD200–CD200R1 axis.

To further explore this hypothesis, we followed four different approaches. First, we examined whether increasing the number of migrating cells could amplify inflammation. To this end, we isolated skin-derived CD2^+^MHC-II^+^CCR2^+^ myeloid precursors for adoptive transfer on day 7 in IL-23OE in BALB/c mice. We also isolated a population of CD11c^−^CCR7^−^CLEC10A^+^CD11b^+^ macrophages corresponding to the Kaede^RED^ population in the joint (Supplementary Fig. 3). The sorted cells were reintroduced into the ankle joint of BALB/c mice on day 7 of IL-23OE to coincide with the decrease in CD200^+^ fibroblasts and to increase the pool of migrated cells in the joint. The level of inflammation was assessed histologically and by MRI on day 14. Adoptive transfer resulted in increased arthritis severity at this early time point (Extended Data Fig. 5c), highlighting the critical proinflammatory role of CD2^+^MHC-II^+^CCR2^+^ myeloid precursors in an arthritis-conductive environment. Second, we used IL-23OE in the arthritis-resistant C57BL/6 inbred strain and intervened every second day with anti-CD200 treatment (OX-90) to block the suppression of skin-derived precursors by resident CD200^+^ fibroblasts. After blocking CD200, initially arthritis-resistant animals developed joint inflammation on day 21 (Fig. 4l), supporting the critical gate-keeping role of CD200^+^ fibroblasts interacting with migrating CD2^+^MHC-II^+^CCR2^+^ myeloid precursors. Third, we induced skin psoriasis in BALB/c mice using topical imiquimod, which is a locally restricted model, to test our hypothesis in a model that is independent of systemic IL-23OE. Using this model, we observed an increase in CD2^+^MHC-II^+^CCR2^+^ myeloid precursors in joints on day 7, but no decrease in CD200^+^ fibroblasts (Extended Data Fig. 5d). Again, we intervened every second day with anti-CD200 treatment (OX-90) to block the suppression of skin-derived precursors by resident CD200^+^ fibroblasts. In line with our second approach, on day 21, only animals that received the blocking antibody developed joint inflammation (Extended Data Fig. 5e). Fourth, we used IL-23OE in the arthritis-susceptible BALB/c inbred strain and intervened every 5 days with an agonistic anti-CD200R1 treatment (OX-110) starting on day 7 to restore the suppression of skin-derived precursors by resident CD200^+^ fibroblasts. After treatment with agonistic anti-CD200R1, arthritis was significantly reduced on day 21 (Fig. 4m), supporting the critical anti-inflammatory signaling mediated by CD200.

### Myeloid precursors activate synovial T cells

**Fig. 5:**
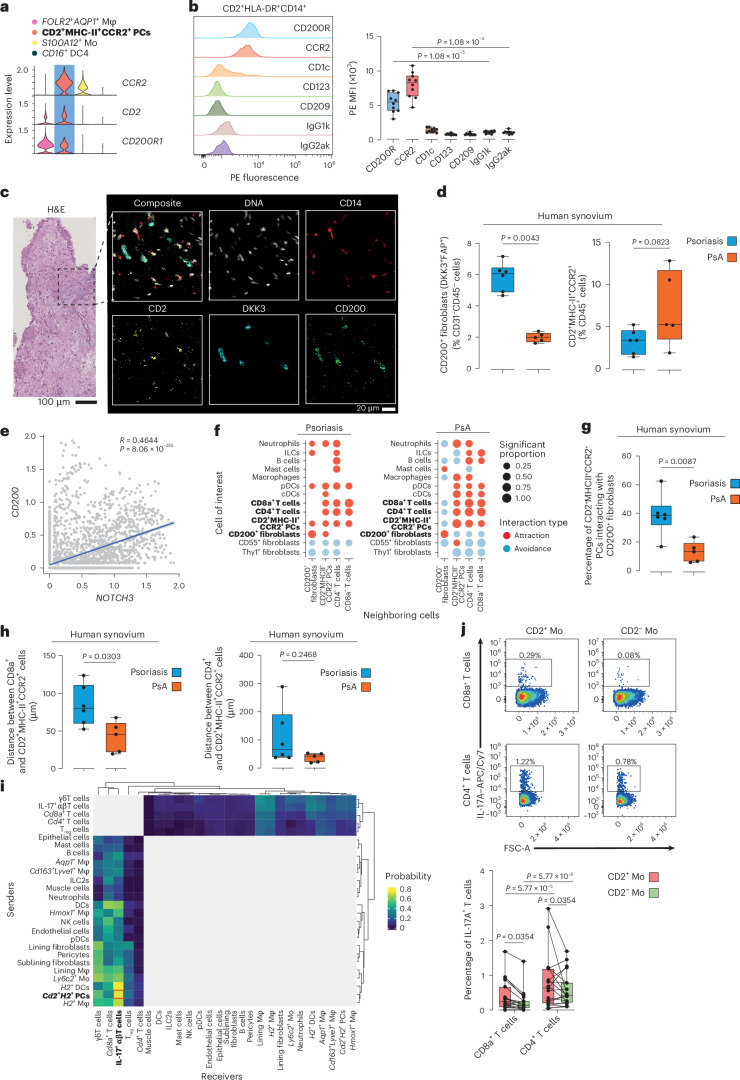
CD200–CD200R1 control the onset of joint inflammation. **a**, Violin plots of ALRA-imputed gene expression among the identified clusters of human synovial myeloid cells from Fig. 3a. **b**, Representative histogram and quantification of the median fluorescence intensity (MFI) of marker expression on CD2^+^HLA-DR^+^CD14^+^ circulating monocytes in PBMCs of individuals with PsA; *N* = 10. *P* values were calculated by two-sided Mann–Whitney *U*-test between target markers and isotype controls. **c**, Representative micrographs of H&E-stained sections of human synovial tissue and selected IMC-stained markers from psoriasis (*N* = 6) and early PsA (*N* = 5). **d**, IMC-based quantification of CD200^+^ fibroblasts and CD2^+^MHC-II^+^CCR2^+^ myeloid precursors in synovial tissue from individuals with psoriasis or early PsA. The graphs show median, quartiles and minimum–maximum; *N* = 6 and *N* = 5, respectively. *P* values were calculated by two-sided Mann–Whitney *U*-test. **e**, *CD200* expression correlated to *NOTCH3* expression in synovial fibroblasts (gray dots) from Extended Data Fig. 4a. The blue line shows the linear fit, and the gray region shows the confidence interval. Two-sided Pearson correlation and BH-adjusted *P* value are shown. **f**, Pairwise interaction test between CD2^+^MHC-II^+^CCR2^+^ myeloid precursors, CD200^+^ fibroblasts and T cells with the surrounding immune and mesenchymal cells in the IMC dataset of synovial tissue from individuals with psoriasis (*N* = 6) or PsA (*N* = 5); cDCs, conventional DCs. **g**, Proportion of CD2^+^MHC-II^+^CCR2^+^ precursors interacting with CD200^+^ fibroblasts in the IMC dataset in **c**. The graphs show the median, quartiles and minimum–maximum; *N* = 6 and *N* = 5, respectively. *P* values were calculated by two-sided Mann–Whitney *U*-test. **h**, Distance between CD8a^+^ and CD4^+^ T cells and CD2^+^MHC-II^+^CCR2^+^ myeloid precursors in the IMC dataset from synovial tissue of individuals with psoriasis (*N* = 6) or PsA (*N* = 5). The graphs show the median, quartiles and minimum–maximum. *P* values were calculated by two-sided Mann–Whitney *U*-test. **i**, Hierarchically clustered heat map showing the CellChat-calculated interaction probability between subclusters in the scRNA-seq dataset of Kaede^tg^ animals. Gray tiles are excluded interactions. **j**, Representative flow cytometry plots showing IL-17A expression in CD8^+^ and CD4^+^ T cells after coculture with CD2^+^MHC-II^+^CCR2^+^ or CD2^−^MHC-II^+^CCR2^+^ myeloid cells. The graphs show quantification of IL-17A expression, correspondingly, as mean, quartiles and minimum–maximum; *N* = 19. *P* values were calculated by two-way ANOVA with a Tukey’s post hoc test. Source data

**Extended Data Fig. 6:**
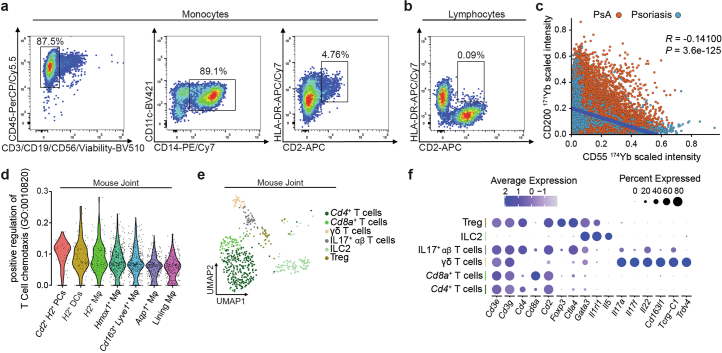
CD2^+^MHC-II^+^CCR2^+^ myeloid precursors trigger IL-17 expression in T cells. (**a**) Representative flow cytometry plots showing the gating strategy for the identification of CD2^+^HLA-DR^+^CD14^+^ circulating monocytes (pre-gated on FSC/SSC) in PBMCs of PsA patients. (**b**) Representative flow cytometry plot showing CD2^+^ HLA-DR^+^ gate on lymphocytes. (**c**) Scaled CD200 signal intensity correlated to scaled CD55 signal intensity in synovial of fibroblasts from psoriasis (yellow dots) or individuals with PsA (orange dots) from Fig. 4i. Blue line shows the linear fit. Two-sided Pearson correlation and *P* value are shown. (**d**) Comparison of the UCell score of the GO-BP “positive regulation of T cell chemotaxis; GO:0010820” among subclusters of myeloid cells in the joint of BALB/c mice in scRNA-seq dataset from Kaede^tg^ mice. (**e**) UMAP plot of the subsets of T cells and ILCs in the joint in the scRNA-seq dataset from Kaede^tg^ mice. (**f**) Expression of the most relevant marker genes among each subclusters identified in **e**.

To challenge our observations made in mice, we analyzed the expression of *CD200R1* on CD2^+^MHC-II^+^CCR2^+^ skin-derived joint-invading myeloid precursors in our human scRNA-seq database (Fig. 5a). Similarly, flow cytometric analysis of PBMCs from individuals with PsA showed an equally high expression of CD200R1 on CD2^+^MHC-II^+^CCR2^+^ skin-derived joint-invading myeloid precursors (Fig. 5b and Extended Data Fig. 6a,b).

We then used imaging mass cytometry (IMC) to detect CD2^+^MHC-II^+^CCR2^+^ myeloid precursors and CD200^+^ (DKK3^+^FAP^+^CD45^−^CD31^−^) fibroblasts in the synovial tissues of individuals with early PsA who had just developed arthritis and individuals with psoriasis without arthritis (Fig. 5c). Consistent with our previous observations in mice and humans, we observed an increase in CD2^+^MHC-II^+^CCR2^+^ myeloid precursors and a significant decrease in CD200^+^ fibroblasts in the synovial tissue during the onset of arthritis in humans (Fig. 5d).

Because close cell–cell contact is essential for effective CD200 signaling, we localized CD200^+^ fibroblasts in the joint. To this end, we analyzed the expression of *CD200* in relation to *NOTCH3* in fibroblasts, as *NOTCH3* has been described to form a decreasing gradient within the synovium from the perivascular space to the lining layer^35^. We found a significant correlation between *NOTCH3* and *CD200* in synovial tissue (Fig. 5e), suggesting that CD200^+^ fibroblasts reside within the perivascular space and distal of the lining layer. Similarly, when we plotted the CD200 signal against the CD55 signal in fibroblasts in the IMC dataset, we found a significant negative correlation (Extended Data Fig. 6c). In this dataset, we also examined the spatial cellular neighborhood of CD2^+^MHC-II^+^CCR2^+^ myeloid precursors and CD200^+^ fibroblasts and quantified their interaction strength. Although both cell populations showed a significant attraction in the noninflamed synovia of individuals with psoriasis, in the inflamed synovia of individuals with PsA, both cell populations showed a significant avoidance (Fig. 5f). Consequently, there was a significant reduction in the percentage of CD2^+^MHC-II^+^CCR2^+^ myeloid precursors interacting with CD200^+^ fibroblasts in individuals with PsA compared to in those with psoriasis (Fig. 5g). These analyses show that the priming of skin-derived recirculating CD200R1-expressing CD2^+^MHC-II^+^CCR2^+^ myeloid precursors by CD200^+^ fibroblasts occurs immediately after entry into the synovial tissue and before development into subsidiary macrophage subsets. The interaction between the two cell populations is reduced in PsA due to the reduced numbers of CD200^+^ fibroblasts.

CD4^+^ and CD8^+^ T cells play a prominant role in the pathogenesis of PsA^40,41^. To investigate the pathogenic potential of CD2^+^MHC-II^+^CCR2^+^ myeloid precursors, we conducted an interaction analysis with T cells. Both the CD4^+^ and CD8^+^ T cell populations demonstrated attraction to CD2^+^MHC-II^+^CCR2^+^ myeloid precursors in the noninflamed synovia of individuals with psoriasis and the inflamed synovia of individuals with PsA (Fig. 5f). Notably, we found a significant reduction in the distance between CD2^+^MHC-II^+^CCR2^+^ myeloid precursors and particularly CD8^+^ T cells in individuals with PsA, indicating enhanced interaction (Fig. 5h). Analysis of the scRNA-seq dataset from Kaede^tg^ animals revealed that CD2^+^MHC-II^+^CCR2^+^ myeloid precursors exhibited the highest UCell scores for ‘positive regulation of T cell chemotaxis’ (GO:0010820; Extended Data Fig. 6d) and had one of the highest interaction probabilities with IL-17^+^ T cells (Fig. 5i and Extended Data Fig. 6e,f). To further evaluate this interaction, we sorted CD4^+^ and CD8^+^ T cells from PBMCs of individuals with PsA and cocultured them with either CD2^+^MHC-II^+^CCR2^+^ or CD2^−^MHC-II^+^CCR2^+^ myeloid cells, which were correspondingly isolated from the same individuals. Only CD2^+^MHC-II^+^CCR2^+^ myeloid precursors positively influenced IL-17 expression in both T cell subsets, contrasting with CD2^−^MHC-II^+^CCR2^+^ myeloid cells (Fig. 5j). In conclusion, the greater spatial proximity and functional interaction between myeloid precursors and CD8⁺ T cells in the synovium of individuals with PsA likely induces IL-17 expression in local T cells. This finding corroborates the concept that skin-derived CD2^+^MHC-II^+^CCR2^+^ myeloid precursors play a key role in the pathogenesis of PsA.

## Discussion

Herein, we describe a two-step process that drives the progression from psoriatic skin to joint disease. Psoriasis induced proinflammatory CD2^+^MHC-II^+^CCR2^+^ myeloid precursors to migrate from the skin to joints, but their migration alone was insufficient to induce arthritis. Rather, the local microenvironment, particularly CD200^+^ fibroblasts, acted as gatekeepers of arthritis, engaging the checkpoint receptor CD200R1 on migrating CD2^+^MHC-II^+^CCR2^+^ myeloid precursors. Consequently, intervention with an antibody to CD200 polarized CD2^+^MHC-II^+^CCR2^+^ myeloid precursors toward a proinflammatory phenotype in vitro and allowed the in vivo spreading of psoriatic disease from the skin to the joints even in initially arthritis-resistant mice.

Our findings shed light on a previously unexplored aspect of psoriatic disease pathogenesis. Although tracking cell migration in humans is technically challenging due to a lack of labeling options, next-generation scRNA-seq has enabled tracking of adaptive lymphocytes through clonal expansion analysis. For example, shared T cell repertoires have been identified between synovial fluid and blood T cells in individuals with PsA and spondylarthritis^42^ or between skin and blood in healthy individuals^9^. Skin-resident T cells express high levels of cutaneous lymphocyte-associated antigen (CLA), allowing them to be tracked in the peripheral blood. However, it has been described that CLA^+^ T cells preferentially (re)migrate to the skin rather than the synovium in individuals with PsA^43^. As these methods do not capture the full spectrum of leukocytes, we used a translational approach using stable photoconversion-based labeling in mouse models of psoriasis and arthritis. We found that most skin-derived cells within the joint belonged to distinct myeloid populations, most of which are typically characterized as tissue resident and nonmigratory^17^. However, we identified a migrating myeloid precursor subset, CD2^+^MHC-II^+^CCR2^+^, which gave rise to the broader pool of skin-derived myeloid cells observed in the joint. Extrapolating to humans, we confirmed the conserved presence of these cells in the skin and synovia across species. Adopting the MAESTER method^30^ to track mitochondrial variants based on mRNA, we corroborated the skin–joint axis in humans. We demonstrated an increased abundance of precursors in individuals with psoriasis or PsA compared to healthy control individuals, suggesting that the migration of these precursors occurs independently of joint involvement, but also constituting a prerequisite to spread inflammation from the skin to the joints. The consistency of our results across species highlights the conserved nature of this pathomechanism and opens up new perspectives for developing novel diagnostic and therapeutic strategies.

Our study demonstrated that the development of arthritis depends on the differentiation of CD2^+^MHC-II^+^CCR2^+^ myeloid precursors into highly proinflammatory mononuclear phagocytes. Computational analyses, such as pseudotime, RNA velocity and interactome, revealed the dynamic behavior of these precursors after entering the synovial tissue. The articular microenvironment, particularly CD200^+^ fibroblasts, played a crucial role in modulating precursor activation and differentiation by activating the checkpoint receptor CD200R1, thereby suppressing inflammation. Conversely, blocking CD200 in different arthritis-resistant models resulted in the onset of arthritis, highlighting the inflammatory potential of these skin-derived myeloid precursors. They were also highly effective at inducing IL-17 expression in T cells. Interestingly, we found no evidence of cutaneous priming of CD2^+^MHC-II^+^CCR2^+^ myeloid precursors, emphasizing the influence of the synovial microenvironment on disease progression from the skin to the joint. We recently identified a CD200^+^ fibroblast subtype that contributes to the resolution of inflammation in arthritis^34^, and we have now demonstrated its involvement in suppressing the development of arthritis. This highlights the CD200–CD200R1 axis as a promising target for preventive medicine.

Although most cases of PsA are preceded by cutaneous psoriasis, a notable proportion of individuals develop PsA without any prior skin symptoms. Genetic studies reveal both shared and distinct genetic factors between skin-limited psoriasis and PsA^44,45^, suggesting partially divergent pathogenic pathways. Although this study focused on the predominant skin-to-joint axis, we acknowledge the need for future research to explore alternative mechanisms.

In conclusion, our study sheds light on the cellular and molecular mechanisms driving the spread of psoriatic skin inflammation to the joints in psoriatic disease. Similar to tumor metastasis, this process requires migrating cells and a supportive microenvironment. By identifying CD2^+^MHC-II^+^CCR2^+^ myeloid precursors, their interaction with synovial fibroblasts and the CD200–CD200R1 axis as pivotal factors in this process, we have also proposed new therapeutic targets that could improve patient outcomes and advance personalized medicine in the treatment of this debilitating disease.

## Methods

### Experimental approaches

Experiments were not performed in a blinded fashion, except where specifically stated. There were no exclusion criteria for human or animal experimentation. Mice were stratified by sex and then randomized to the different groups. Cells from human donors were also randomized.

### Human studies and characteristics

Human research was conducted in accordance with protocols approved by the institutional review boards of the Friedrich-Alexander-University (FAU) of Erlangen-Nürnberg, Fondazione Policlinico Gemelli IRCCS and Hospital Clinic de Barcelona after receiving informed consent. Ultrasound-guided, minimally invasive synovial tissue biopsies were collected from individuals with early PsA (*N* = 6) and those with psoriasis (*N* = 8) at the SYNGem Biopsy Unit of the Fondazione Policlinico Universitario A. Gemelli RCCS (ID: 4951-2022) and at the Uniklinikum Erlangen (ID: 18-334_4-Bio). Additional synovial tissue from healthy donors (*N* = 3) was obtained at the Hospital Clinic de Barcelona (ID: HCB/2 020/0100). Punch biopsies (3 mm) from lesional skin were collected from individuals with psoriasis (*N* = 3) in Erlangen. Blood samples were collected from individuals with PsA (*N* = 29) in Erlangen. Participant information is provided in Supplementary Tables 1 and 2. All participants included fulfilled the 2006 CASPAR classification criteria for PsA^46^. Additional human datasets were obtained from online databases listed in the Data availability section and referenced in the text and figure legends.

### Mice

BALB/c JRj and C57BL/6 NRj male and female mice were obtained from Janvier Laboratories. C-Kaede^tg^ (C.Cg-Tg(CAG-tdKaede)15Utr) and B6-Kaede^tg^ (B6.Cg-Tg(CAG-tdKaede)15Utr) mice were described in Tomura et al.^16^ and were kindly provided by Y. Miwa and M. Tomura from the RIKEN BioResource Research Center through the National BioResource Project of the MEXT/AMED, Japan. C-Kaede^tg^ and B6-Kaede^tg^ mice were backcrossed to BALB/c JRj and C57BL/6 NRj mice, respectively, and only heterozygous Kaede^tg^ male and female mice were used in the experiments. All animals were maintained under specific pathogen-free conditions with a 12-h light/12-h dark cycle, fed chow (sniff Spezialdiäten, V1534-000) and given water ad libitum. Room air temperature and cage climate were standardized at 20–24 °C, 45–65% relative humidity and 15–20 air changes per h. Mice were housed in groups of three to five animals per cage. A period of 1 week was observed between delivery and the start of the study for test animals that did not come from our own breeding program. All mice were 8–10 weeks old at the start of the study. The animal experiments were conducted in accordance with local regulations and were approved by the government of Lower Franconia (protocols 55.2-2532-2-1061, 55.2-2532-2-1886 and 55.2-2532-2-2157).

### IL-23-induced model of PsA

*Il23* minicircle treatment (3 µg per mouse) was administered on the first day of scoring. Scoring was continued until day 21, the end of the model. The development of skin symptoms (scaling) was monitored and reported as follows: grade 0 (no scaling), 1 (mild scaling), 2 (moderate scaling) and 3 (severe scaling). C-Kaede^tg^ and B6-Kaede^tg^ heterozygous mice were treated with 6 µg per mouse. The *Il23* vector, which encodes the two subunits IL-12b (IL-12p40) and IL-23a (IL-23p19) linked by a flexible region under the control of the albumin promoter for efficient expression in hepatocytes, followed by a secretion sequence for efficient release into the circulation, was kindly provided by S. Wirtz (Department of Medicine 1, FAU Erlangen-Nürnberg). The vector was produced in *Escherichia coli* DH5α grown in terrific broth medium without removal of vector backbone. Plasmids were purified using a PureLink HiPure Plasmid Maxiprep kit (Thermo Fisher), followed by a MiraCLEAN Endotoxin Removal kit (Mirus) to ensure efficient removal of endotoxin. Next, 3 µg of naked plasmid DNA in Ringer’s solution was administered into the lateral tail vein by hydrodynamic gene transfer at a volume equivalent to 10% of body weight^47,48^. Blocking anti-CD200 (OX-2; clone OX-90, BioXCell) was administered intraperitoneally every other day (100 µg per dose) to C57BL/6 animals, and treatment was started on the same day as *Il23* treatment. Agonistic anti-CD200R1 (OX-2R; clone OX-110, BioLegend) was administrated subcutaneously every 5 days to BALB/c animals, and treatment was started 7 days after *Il23* treatment.

### Imiquimod-induced psoriasis model

A 2 × 3 cm area on the dorsal skin of female BALB/c mice was shaved using an electric shaver, and any remaining hair was removed using depilatory cream (Veet). A total of 100 mg of Aldara cream (Viatris), containing 5% imiquimod, was applied daily for either 7 or 21 consecutive days. Mice treated for 21 days concomitantly received intraperitoneal injections of anti-CD200 (OX-2; clone OX-90, Bio X Cell) or a rat IgG2a isotype control (clone RTK2758, BioLegend) every other day (100 µg per dose).

### UV light exposure

Mice were anesthetized with O_2_/isoflurane (3% (vol/vol) isoflurane, 1.2 l min^−1^ O_2_) and placed on a heating pad (37 °C). The psoriatic skin of the lower back and proximal parts of the legs was shaved, whereas the rest of the body, including the distal parts of the legs and hind paws, was shielded with aluminum foil. The skin was exposed to a UV light source for 10 min according to a previous protocol^16^. Briefly, a BlueWave LED PrimeCure UVA QX4 (Dymax) with a 3-mm-diameter lens was used at 51% power, and a distance of 16 cm was maintained between the skin and the lenses. The exposure was repeated for two consecutive days (–48 and –24 h) before death.

### MRI

In vivo MRI of the hind paws was performed with a preclinical 7T MRI (BioSpec, Bruker BioSpin) using a volume resonator (RF RES 300 1H 075/040 QSN TR). The imaging protocol included pre- and postcontrast T1-weighted spin echo sequences and a T2 TurboRARE short tau inversion recovery sequence. During imaging, the mice received an intravenous bolus injection of a low-molecular-weight gadolinium chelating agent (0.15 mmol kg^−1^ gadobutrol, Bayer Vital) over a time period of 10 s via a tail vein catheter. Mice were placed on a heating pad (37 °C) and anesthetized with O_2_/isoflurane (3 % (vol/vol) isoflurane, 0.5 l min^−1^ O_2_), and eye ointment was applied. The volume of joint inflammation was evaluated on either short tau inversion recovery or T1-weighted images after subtraction of pregadolinium from postgadolinium was performed, and the volume of contrast-enhancing areas of the ankle joint was quantified by manual segmentation (3D Slicer^49^, version 5.6.1).

### Histological processing of mouse samples

Hind paw joints were fixed in PBS containing 4% (wt/vol) formaldehyde for 16–24 h. After removal of the skin, the bone tissue was decalcified in 0.5 M EDTA (pH 7.4) before embedding and sectioning. Tissues were dehydrated, infiltrated and embedded in paraffin, cut into 1-µm-thin sections and mounted on standard histological slides. Thin sections were deparaffinized by heating the slides at 65 °C for 30 min, washed in Histo Clear (National Diagnostics) and rehydrated through a series of 100% (vol/vol) ethanol, 95% (vol/vol) ethanol, 80% (vol/vol) ethanol, 60% (vol/vol) ethanol and water. Mayer’s hematoxylin solution (Merck) was applied for 10 min. Excess hematoxylin was washed off, and the stain was blued by rinsing the slides for 10 min in tap water. Eosin counterstain (0.3% (wt/vol) Eosin Y (Sigma) and 0.01% (vol/vol) acetic acid) was applied for 3 min, and excess stain was washed off with deionized water. The sections were again dehydrated in isopropanol and mounted with Roti Mount mounting medium (Carl Roth). Slides were digitized with a NanoZoomer S60 (Hamamatsu), and regions of interest were exported as .TIFF files. Epidermal thickness was measured using the NDPView2 (2.7.39) program (Hamamatsu). A periarthritis score was established to assess the inflammatory infiltrate in the joints: grade 0 (no infiltrate), 1 (mild infiltration), 2 (moderate infiltration) and 3 (severe infiltration).

### Microscopy image analysis

Immediately after death, the hind paws were dissected, and the skin was removed. Samples were immersed in –20 °C cooled acetone and stored at –20 °C overnight for fixation. For light sheet microscopy, ethyl cinnamate was used to clear the tissue, and imaging was performed using an UltraMicroscope II (LaVision). Kaede^GREEN^ and Kaede^RED^ were detected in the 488 and 594 channels. For fluorescence microscopy, the hind paws were decalcified in decalcification buffer for 2 days after fixation. Tissues were cryoprotected in 30% sucrose and 10% PVP in PBS, embedded in 30% gelatine and sectioned at 40 µm. Imaging was conducted using a Leica Thunder Imager 3D Assay with a ×63/1.4- to 0.6-NA oil immersion objective, with subsequent large volume computational clearing and maximum intensity projection. Imaging analysis was performed with IMARIS X64 (Oxford Instruments) software (v. 9.3.0) and Fiji (v. 1.52)^50^.

### Microcomputed tomography imaging and analysis

Structures of tibial bones and paws were measured with a SCANCO Medical μCT 40 or µCT45 scanner and analyzed with SCANCO Application Center evaluation software for segmentation, three-dimensional morphometric analysis and density and distance parameters (SCANCO Medical). Three-dimensional modeling of the bone was performed with optimized grayscale thresholds of the operating system Open VMS (SCANCO Medical) and XamFlow-Workflow^51^.

### Preparation of single-cell suspensions from mouse tissue

After death, the skin covering both legs was shaved and isolated, the subcutaneous fat was removed, and the tissue was minced with scalpels. Legs with intact ankles were dissected, and the nails were cut off. The tibia was removed by dislocation, and the tendons and joint capsules were cut to facilitate enzymatic dissociation. Skin and joint tissues were digested in RPMI 1640 containing 1.2 mg ml^−1^ collagenase D (Roche), 0.6 (skin) and 0.2 (joint) mg ml^−1^ Dispase II (Sigma-Aldrich) and 0.2 mg ml^−1^ DNase I (Roche). Samples were incubated three times at 37 °C for 20 min with constant shaking (2,000 rpm) on a thermal shaker (Eppendorf). After 20 min, the supernatant was collected and filtered (70 µm), and fresh digestion medium was added. The digestion was repeated for a total of three times. Red blood cell (RBC) lysis was performed using RBC lysis buffer (BioLegend) according to the manufacturer’s recommendations. Debris removal solution (Miltenyi Biotec) was used to remove impurities according to the manufacturer’s protocol. Small intestine samples were collected and, after removal of the mesentery, presoaked and rinsed in cold DMEM (Gibco) containing 20% (vol/vol) fetal bovine serum (FBS; Gibco). The tissue was then cut longitudinally and incubated with agitation in HBSS (without Ca^2+^; Gibco) containing 2 mM EDTA. The mucosal epithelium was isolated, and the subepithelial tissue was further washed with HBSS (without Ca^2+^; Gibco) and cut into pieces (5 mm^2^) for enzymatic digestion with HBSS (Gibco; with Ca^2+^ and 2% (vol/vol) FBS) containing 3.0 mg ml^−1^ collagenase D (Roche) and 0.2 mg ml^−1^ DNase I (Roche). Samples were incubated three times for 10 min at 37 °C with constant shaking (800 rpm) on a thermal shaker (Eppendorf). The resulting cell suspension was filtered (100 µm), washed with DMEM (Gibco) containing 10% (vol/vol) FBS (Gibco) and pooled with mucosal isolated cells. Cell counts were determined by trypan blue staining using an automated cell counter (Bio-Rad). For the isolation of bone marrow cells, the femur and tibia were collected, cleaned of muscle and tendons and cut at both ends. After centrifugation at 10,000*g* for 15 s, the resulting pellet was treated with RBC lysis buffer (BioLegend) and washed. The resulting cell suspensions from all tissues were filtered (40 µm), and cell counts were determined by trypan blue staining using an automated cell counter (Bio-Rad).

### Preparation of single-cell suspensions from human tissue

Fresh human skin and synovial tissue or fresh frozen human synovial tissues were thawed, thoroughly minced and digested in RPMI 1640 containing 2 mg ml^−1^ collagenase D (Roche), 0.2 mg ml^−1^ Dispase II (Sigma-Aldrich) and 0.2 mg ml^−1^ DNase I (Roche) for synovial tissue or 7.5 mg ml^−1^ collagenase D (Roche), 2.5 mg ml^−1^ Dispase II (Sigma-Aldrich) and 0.5 mg ml^−1^ DNase I (Roche) for skin. Samples were incubated three times at 37 °C for 20 min with constant shaking (2,000 rpm) on a thermal shaker (Eppendorf). After 20 min, the supernatant was collected and filtered (70 µm), and fresh digestion medium was added. Digestion was repeated for a total of three times, and RBC lysis was performed using RBC lysis buffer (BioLegend) according to the manufacturer’s recommendations. The resulting cell suspensions were filtered (30 µm) and counted in trypan blue using a Neubauer counting chamber (Brand).

### PBMC isolation

Human EDTA blood samples were collected from individuals with PsA at the outpatient clinic of the University Hospital Erlangen, as part of routine diagnostics. Written informed consent was obtained from all individuals. PBMCs were purified using Lymphosep (Biowest). Briefly, 6 ml of EDTA blood was mixed with 6 ml of DPBS (Gibco), underlaid with 4 ml of Lymphosep and centrifuged at 1,000*g* for 30 min without breaking. The plasma component was discarded, and the PBMC ring was collected and washed in 50 ml of PBS by centrifugation at 200*g* for 30 min. Erylysis (RBC Lysis buffer, BioLegend) and a second centrifugation at 100*g* for 30 min were performed to remove RBCs and thrombocytes, respectively.

### Flow cytometry and cell sorting

Cells were stained for 30 min on ice in PBS containing 2% (vol/vol) FBS and 5 mM EDTA and then fixed with 1% formaldehyde for 20 min. Single-dye staining and fluorescence minus one controls were used to compensate and set up the gating, respectively. Dead cells were excluded using FVS510 (1:200; BD Biosciences) or eFluor 780 Fixable Viability Dye (1:4,000; eBioscience). Detailed information about all antibodies is listed in Supplementary Table 3. Anti-CD45 (30-F11; 1:1,000 dilution), anti-CD3ε (145-2C11; 1:100 dilution), anti-CD45R/B220 (RA3-6B2; 1:200 dilution), anti-Ly6G (1A8; 1:100 dilution) and anti-CD11b (M1/70; 1:1,000 dilution) were used to identify/sort Kaede^RED^ skin-derived migrating cells in the hind paws. Antibodies used for identification of migrating myeloid precursors in the skin and in the hind paws included anti-CD45 (30-F11; 1:1,000 dilution), anti-Ly6G (1A8; 1:1,000 dilution), anti-CD11b (M1/70; 1:1,000 dilution), anti-CD2 (RM2-5; 1:1,000 dilution), anti-I-A/I-E (M5/114.15.2; 1:500 dilution), anti-CD3ε (145-2C11; 1:100 dilution), anti-CD45R/B220 (RA3-6B2; 1:200) and anti-CD11c (N418; 1:500 dilution). Antibodies used to analyze CD200 expression in the fibroblast compartment included anti-CD140a (APA5; 1:500 dilution), anti-CD45 (30-F11; 1:1,000 dilution), anti-CD90.2 (30-H12; 1:500 dilution), anti-podoplanin (8.1.1; 1:200 dilution), anti-CD31 (390; 1:500 dilution), anti-CD49f (GoH3; 1:200 dilution) and anti-CD200 (OX-90; 1:100 dilution). Antibodies used for adoptive cell transfer experiments included anti-CD45 (30-F11; 1:500 dilution), anti-Ly6G (1A8; 1:500 dilution), anti-CD11b (M1/70; 1:200 dilution), anti-CD2 (RM2-5; 1:500 dilution), anti-I-A/I-E (M5/114.15.2; 1:100 dilution), anti-CD3ε (145-2C11; 1:100 dilution), anti-CD45R/B220 (RA3-6B2; 1:200 dilution), anti-CD11c (N418; 1:500 dilution), anti-CCR7 (4B12; 1:100 dilution) and anti-CLEC10A (LOM-14; 1:100 dilution).

PBMCs were freshly analyzed by flow cytometry with anti-CD2 (RPA-2.10; 1:1,000 dilution), anti-CCR2/CD192 (K036C2; 1:500 dilution), anti-CD200R1 (OX-108; 1:100 dilution), anti-HLA-DR (L243; 1:1,000 dilution), anti-CD14 (M5E2; 1:100 dilution), anti-CD16 (3G8; 1:100 dilution), anti-CD45 (HI30; 1:100 dilution), anti-CD3 (UCHT1; 1:100 dilution), anti-CD19 (HIB19; 1:200 dilution), anti-CD56 (HCD56; 1:200 dilution), anti-CD11c (3,9; 1:100 dilution), anti-CD1C (L161; 1:1,000 dilution), anti-CD123 (6H6; 1:1,000 dilution) and anti-CD209 (9E9A8; 1:200 dilution) in the myeloid fraction in humans together with isotype IgG1, κ (MOPC-21; 1:100 dilution) and IgG2a, κ (MOPC-173; 1:200 dilution). PBMCs used for sorting and analysis of coculture experiments were stained for anti-CD3 (UCHT1; 1:200 dilution), anti-CD19 (HIB19; 1:200 dilution), anti-CD8a (SK1; 1:1,000 dilution), anti-CD4 (A161A1; 1:200 dilution), anti-CCR2 (K036C2; 1:500 dilution), anti-HLA-DR (L243; 1:200 dilution), anti-CD2 (RPA-2.10; 1:200 dilution), anti-CD1C (L161; 1:500 dilution), anti-CD123 (6H6; 1:500 dilution) and anti-IL-17A (BL168; 1:500 dilution). IL-17A was stained intracellularly using an IC fixation kit (Thermo Fisher) according to the manufacturer’s recommendations. All antibodies were purchased from BioLegend unless stated otherwise. Acquisition of cytometry data was performed with Beckman Coulter Gallios software v 1.2, while a Beckman Coulter MoFlo Astrios EQ (running with BD Astrios Summit v6.3.1; Beckman Coulter) was used for cell sorting. Sorted populations were reanalyzed to determine target cell purity after sorting (>98% purity). Flow cytometry data were analyzed with FlowJo (v. 10.10, BD Biosciences).

### Adoptive cell transfer

Donor BALB/c mice were killed 7 days after *Il23* treatment. Hind paws were isolated and digested, and resulting cell suspensions were stained with a cocktail of flow cytometry antibodies, as described above. Skin-derived myeloid cells were sorted from the hind paws of donors and adoptively transferred to hind paws of recipient BALB/c mice via intra-articular injection of the tarsal joints (50,000 cells per 20 µl per paw) 7 days after *Il23* treatment.

### Imaging cytometry

All samples were acquired using a 12-channel Amnis ImageStreamX Mark II (Cytek Biosciences) imaging flow cytometer equipped with three excitation lasers (405 nm (120 mW), 488 nm (200 mW) and 642 nm (150 mW)) and a MultiMag with three objectives lenses (×20, ×40 and ×60 magnification). Samples were acquired at ×60 magnification, and the excitation lasers (405 nm, 488 nm and 642 nm) were used at 80 mW, 5 mW and 150 mW, respectively.

Image analysis was performed using image-based algorithms in ImageStream Data Exploration and Analysis Software (IDEAS 6.2.189, Cytek Biosciences). Typical files contained imagery for 50,000 to 100,000 cells. Analysis was restricted to single cells in best focus. Single cells were identified by their intermediate size (area) and high aspect ratio (minor axis divided by the major axis) compared to debris (small area and a range of aspect ratios depending on the shape of the debris) and doublets (large area and small aspect ratio). Out-of-focus events were excluded by using the feature brightfield gradient RMS, a measurement of image contrast.

### Cell culture

Mouse fibroblast–macrophage cocultures were prepared as follows. Single-cell suspensions from digested hind paws of healthy C57BL/6 mice were seeded in complete DMEM/F12 containing 10% FBS (Gibco), 100 U ml^−1^ penicillin–streptomycin (Gibco), 0.5 μg ml^−1^ amphotericin B (Gibco) and 20 mM L-glutamine (Sigma) for fibroblast expansion. The medium was changed on day 3, and the cells were passaged on day 6 using trypsin-EDTA (0.25%; Gibco). In total, 1 × 10^5^ cells of the second passage were seeded per well in a six-well plate. In parallel, 20 × 10^6^ bone marrow cells obtained from the same mice were seeded in IMDM (Gibco) containing 10% FBS (Gibco), 100 U ml^−1^ penicillin–streptomycin (Gibco), 0.5 μg ml^−1^ amphotericin B (Gibco), 20 mM L-glutamine (Sigma) and 20 ng ml^−1^ recombinant mouse macrophage colony-stimulating factor (BioLegend) in a 100-mm Petri dish. The medium was changed on day 4, and on day 6 macrophages were detached using Accutase (Gibco). In total, 5 × 10^5^ cells were seeded with fibroblasts in complete IMDM enriched with 20 ng ml^−1^ recombinant mouse macrophage colony-stimulating factor. The following day, anti-CD200 (OX-2, clone OX-90, BioLegend; 10 µg ml^−1^) or isotype control (clone RTK2758, BioLegend) was added to the medium. After 24 h, the cells were collected, and RNA isolation was performed as described below.

Human monocyte–T cell cocultures were prepared as follows. Flow cytometry-based cell sorting was used to isolate CD2^+^ and CD2^−^ myeloid cells as well as autologous CD8^+^ or CD4^+^ T cells from PBMCs of individuals with PsA. Cells were cultured in CD3-coated (1 μg ml^−1^ anti-human CD3 (clone UCHT1) in DPBS (Gibco), 4 °C overnight) 96-well, flat-bottom plates at a ratio of 1:5 (myeloid cells:T cells) in RPMI 1640 supplemented with 10% heat-inactivated FBS, 2 mM L-glutamine, 100 U ml^−1^ penicillin, 100 µg ml^−1^ streptomycin, 1× nonessential amino acid solution and 50 µM β-mercaptoethanol. Human macrophage colony-stimulating factor (50 ng ml^−1^, BioLegend), recombinant human IL-2 (10 ng ml^−1^, BioLegend), recombinant human TGFβ (5 ng ml^−1^, BioLegend), recombinant human IL-6 (25 ng ml^−1^, BioLegend), recombinant human IL-1β (10 ng ml^−1^, BioLegend) and recombinant human IL-23 (25 ng ml^−1^, BioLegend) were added to the culture. Cells were maintained at 37 °C and 5% CO_2_ for 6 days, with medium and cytokine replenishment every 48 h. On day 6, cells were restimulated with cell activation cocktail (PMA/ionomycin) without brefeldin A (1:1,000 dilution; BioLegend) for 1 h, followed by monensin and brefeldin A (1:1,000 dilution; BioLegend) for 3 h. Then cells were collected for analysis by flow cytometry as described above.

### Gene expression analysis

RNA isolation was performed using a Nucleo Spin RNA isolation kit according to the manufacturer’s protocol (Macherey-Nagel). Immediately following extraction, total RNA concentration and ratio of absorbance at 260 and 280 nm (A_260_:A_28__0_) of each sample were determined using a NanoDrop 2000 (Thermo Scientific). In total, 8.2 μl of RNA was used to transcribe mRNA to cDNA, following the manufacturer’s protocol (Applied Biosystems). cDNA synthesis was performed in a total volume of 20 μl, containing 8.2 μl of RNA, 2 μl of 10× PCR buffer Ⅱ, 4.4 μl of MgCl_2_ solution (25 mM), 4 μl of DNTP mix, 0.5 μl of 10× RT random primers, 0.4 μl of RNase inhibitor and 0.5 μl of Multiscribe Reverse Transcriptase. The reaction mixture was incubated at 20 °C for 10 min, followed by 48 °C for 30 min and 95 °C for 5 min to inactivate the enzyme. Real-time PCR was performed in triplicate using SYBR Green master mix (Applied Biosystems) and a QuantStudio 6 Real-Time PCR System v. 1.3. Fold change expression of target genes was calculated by the change in cycling threshold (Δ*C*_t_), ΔΔ*C*_t_ and ${2}^{-\mathrm{\varDelta \varDelta }{C}_{{\rm{t}}}}$ comparative method for relative quantification after normalization. The endogenous control was *B2m*. A list of primers is provided in Supplementary Table 3.

### Droplet-based scRNA-seq libraries

Skin and joint cells from six C-Kaede^tg^ and nine B6-Kaede^tg^ mice with equal sex distribution were pooled and sorted for Ly6G^−^ viable Kaede^RED^ CD45^+^, Kaede^GREEN^ CD45^+^ and Kaede^GREEN^ CD45^−^ fractions on day 7 of IL-23OE. Purified cells were stained for 30 min on ice in PBS containing 1% (wt/vol) bovine serum albumin with hashtag antibodies (TotalSeq-B0301/B0302/B0303/B0304B0305 anti-mouse hashtag 1/2/3/4/5, respectively, and anti-APC-B0987 (customized), all from BioLegend) to distinguish all six fractions per inbred strain. Cells were washed, counted and concentrated to 1,000 cells per µl before pooling. In total, 24,000 cells (hyperloading) equally pooled from each strain were loaded into a single well of a Chromium Chip G (10x Genomics). Human synovial cells were loaded immediately after tissue dissociation with up to 25,000 cells in a single well of a Chromium Chip G (10x Genomics), as described above. The 3′ gene expression libraries were generated using a Chromium Next GEM Single Cell 3′ Kit 3.1 with a 3′ Feature Barcode kit and dual indexing (10x Genomics protocol CG000316 Rev C). Libraries were sequenced as paired end, 150 base pairs (bp) by Illumina sequencing to 65–80% saturation. Reads were mapped to the mm10 mouse genome (GENCODE vM23/Ensembl 98) or GRCh38 human genome (GENCODE v32/Ensembl98) using the 10x Genomics Cell Ranger pipeline (6.0.0 and 7.2.0, respectively) with default settings. Detailed descriptions of the analysis of the RNA-seq data are included in the Supplementary Information.

### Generation of MAESTER libraries

To trace the lineage of specific cell populations and investigate potential migration from the skin to joints in psoriatic disease, we applied the MAESTER protocol^30^. In brief, single-cell suspensions were generated from paired lesioned skin and synovial biopsies obtained from individuals with PsA or psoriasis at risk and loaded onto a 10x Genomics Chromium controller using Single Cell 3′ v3.1 chemistry. Following droplet encapsulation and barcoding, cDNA synthesis and amplification were performed according to the manufacturer’s protocol. Twenty-five percent of cDNA was processed for standard scRNA-seq according to the manufacturer’s recommendations as described above, while 10 ng of the remaining cDNA was processed using the MAESTER workflow. This included an initial amplification, followed by a targeted PCR to enrich mitochondrial DNA amplicons and a final PCR to append sequencing adapters and sample indices. Sequences of mitochondrial DNA-enriching primers and sequencing adapters are listed in the original publication^30^.

Libraries were sequenced on an Illumina NovaSeq X system using standard 10x Genomics settings for scRNA-seq and a custom configuration for MAESTER libraries (28 bp read 1, 8 bp i7, 8 bp i5, 256 bp read 2). Sequencing data were demultiplexed and aligned to both nuclear and mitochondrial genomes to enable integrated transcriptomic and lineage analysis at single-cell resolution. Reads were mapped to the GRCh38 human genome (GENCODE v32/Ensembl98) using the 10x Genomics Cell Ranger pipeline (9.0.1) with default settings.

### IMC

Synovial tissue slides were deparaffinized by heating the slides for 30 min at 65 °C and washing in Histo Clear (National Diagnostics) and rehydrated through a series of 100% (vol/vol) ethanol, 95% (vol/vol) ethanol, 80% (vol/vol) ethanol, 60% (vol/vol) ethanol and water. Heat-induced epitope retrieval was performed for 30 min at 95 °C in Tris-EDTA buffer (10 mM Tris and 1 mM EDTA, pH 9.2). After cooling for 15 min, blocking was performed in Tris buffered saline, pH 7.6, 0.05% (vol/vol) Tween 20 (TBS-T) supplemented with 3% (wt/vol) bovine serum albumin and 1% rabbit serum for 1 h at ambient temperature. A precomposed panel of metal-labeled antibodies (Supplementary Table 3) was applied to the sections. Antibodies were prelabeled with metals or conjugated according to the manufacturer’s instructions for the Maxpar X8 Multimetal Labeling kit (Standard Biotools). Indium chloride was purchased from Sigma-Aldrich (natural abundance, 96% of ^115^In), and ^113^In was purchased from Trace Science. Incubation with antibodies was performed over 16 h at 4 °C in the presence of 0.3 µM Ir-intercalator (Standard Biotools). Slides were washed twice in TBS-T for 10 min, briefly washed in deionized water, air dried and stored in a desiccated environment until analysis. Acquisition was performed at 200 Hz on a Hyperion Imaging System (Standard Biotools, CYTOF software v. 7.1). MCD files were processed using Steinbock (v. 0.16.0)^52^. imcRtools^52^ (v. 1.3.4) and R (v. 4.3.2) were used to load the data and construct a SpatialExperiment^53^. An inverse hyperbolic sine (arcsinh) transformation with a coefficient of 1 was applied to all the datasets. The data were normalized by computing a *z* score, which used the entire dataset as a reference. All data were exported as FCS files and further analyzed with FlowJo Software (v. 10.10, BD Biosciences).

### Spatial analysis of IMC data

Cells were annotated by gating on the known markers using FlowJo software. After annotation, cellular neighbors were identified by using the imcRtools R package^52^. Cellular neighbors were defined by a centroid extension of 40 µm. To quantify interacting cells, neighbors of the cell population of interest were first retrieved, and interacting cells were defined as those that had at least one neighbor of the target cell population. Cellular pairwise interactions were analyzed using the imcRtools R package. The statistical significance of each pairwise interaction was assessed by permutation testing^54^. This approach quantified the average number of neighboring cells for each subpopulation and compared the results to a null distribution generated by randomly reassigning cell-type labels over 1,000 permutations. Pairwise interactions with *P* values below 0.05 were considered statistically significant. Attraction was defined as a higher number of neighboring cells of a specific type than would be expected in a random distribution, whereas avoidance was defined as a lower number of such neighbors than would be expected in the sample. The results are visualized by dot plots generated by using the R package ggplot2. The dot size represents the median proportion of statistically significant interactions found across samples.

### Statistical analysis

Statistical analysis of nonsequencing data was performed using Prism 9. Unless otherwise stated, all data are reported as median, interquartile range and minimum–maximum. No statistical methods were used to prespecify sample sizes, but our sample sizes are similar to those reported in previous publications. Parametric and nonparametric analyses were used where appropriate. When data distribution was assumed to be normal, this assumption was not formally tested. Differences were considered significant when *P* < 0.05. Corrections for multiple testing were made as appropriate.

### Reporting summary

Further information on research design is available in the Nature Portfolio Reporting Summary linked to this article.

## Online content

Any methods, additional references, Nature Portfolio reporting summaries, source data, extended data, supplementary information, acknowledgements, peer review information; details of author contributions and competing interests; and statements of data and code availability are available at 10.1038/s41590-025-02351-z.

## Supplementary information

Supplementary Information Supplementary Methods, Figs. 1–4, Tables 1–3 and references.

Reporting Summary

## Source data

Source Data for Figs. 1–5 and Extended Data Figs. 1–5 Source data for Figs. 1–5 and Extended Data Figs. 1–5.

## Data Availability

Single-cell sequencing data supporting the results of this study have been deposited in the Gene Expression Omnibus (GEO) under accession code GSE228629 and in the European Nucleotide Archive (ENA) under the ArrayExpress accession code E-MTAB-14339. Individual-level mitochondrial variant sequencing data are available under restricted access because of patient privacy and ethical considerations. Qualified researchers can request access by contacting the corresponding author. Publicly available datasets analyzed as part of this study are available under accession codes ENA-ArrayExpress E-MTAB-11791 and E-MTAB-8322 (human synovia), ENA-ArrayExpress E-MTAB-8142 (human skin), GEO GSE194315 (human PBMCs) and GEO GSE122108 (ImmGen mouse mononuclear phagocytes). Further information and requests for resources and reagents should be directed to and will be fulfilled by the lead contact A.R. (andreas.ramming@uk-erlangen.de). Source data are provided with this paper.

## Code availability

All methods and algorithms used in this manuscript are from previously published studies and are cited in the Methods. A list of all software used can be found in Supplementary Table 3. Additional scripts to reproduce the analyses are available from the authors upon request.
