# Supplementary material for: Skin-derived myeloid precursors and joint-resident fibroblasts spread psoriatic disease from skin to joints
Source: Nat Immunol. 2026 Jan 2;27(1):35–47. doi: 10.1038/s41590-025-02351-z (PMC12764428; doi:10.1038/s41590-025-02351-z)
Supplement: Supplementary file 1 — Supplementary Methods, Figs. 1–4, Tables 1–3 and references. [file 41590_2025_2351_MOESM1_ESM.pdf]

nature immunology

Supplementary information

https://doi.org/10.1038/s41590-025-02351-z

Skin-derived myeloid precursors and joint-resident fibroblasts spread psoriatic disease from skin to joints

In the format provided by the authors and unedited

Supplementary Material

Extended Description of Methods

Quality control and annotation of single-cell RNA-seq libraries (mouse)

The two scRNAseq libraries from Kaede^{tg} animals after IL-23OE were first subjected to hashtag antibody oligonucleotide (HTO) demultiplexing using Seurat (4.1.1) [1] in R (4.2.1). The "HTOdemux" function with 0.95 positive quantile resulted in the separation of six libraries encoding for skin and Kaede^{RED} CD45^{+}, Kaede^{GREEN} CD45^{+} and Kaede^{GREEN} CD45^{-} fractions as well as the exclusion of doublets and negative single cells. Quality control was performed to exclude low quality events by individually examining the distribution of quality criteria including the number of unique molecular identifiers (UMIs) >= 1,000, number of genes detected >= 550, complexity (log10(genes/UMI) > 0.8 and the ratio of mitochondrial genes (mitoRatio) < 0.09. Genes expressed in fewer than 10 cells were excluded. From this stage, skin and joint datasets were analysed separately. Unwanted variations after log-normalisation in Seurat ("NormalizeData", "FindVariableFeatures", "ScaleData", "RunPCA") were cycle phase scores and mitoRatio, which were unevenly distributed in the Principal Component (PC) Analysis (PCA) space and regressed out before further analysis. For normalization, "SCTransform" (v2) [2] was applied individually to the raw UMI counts of each library, selecting the top 3,000 variable genes from each dataset. Technical variations resulting from library processing and sequencing were eliminated using Harmony (0.1.0) [3] integration on the merged SCT-transformed counts using the top 50 PCs. The top 50 Harmony components were used for clustering and visualisation. Seurat's default clustering ("FindNeighbors" and "FindClusters") with resolution = 1.5 and 2 resulted in 29 clusters in the skin and 32 clusters in the joint dataset. A single cluster in the joint was identified as debris due to its high mitoRatio and high background gene expression and was therefore removed before repeating harmony integration. In both tissues singleR (1.10.0) [4] using the global Immgen dataset [5] from celldex (1.6.0) [4] with

"tune.thresh" = 0.001 was used for reference based annotation. UMAP visualization was generated using "RunUMAP". Characteristic gene signatures of each cluster were identified using "PrepSCTFindMarkers" on SCT assays followed by "FindAllMarkers" with default parameters. To label the clusters identified by Seurat, both reference-based annotation and manual annotation based on the identified markers [6, 7, 8, 9] were used.

Post-processing of myeloid clusters (mouse)

Harmony integration was repeated for Kaede^{RED} myeloid cells as previously described. Using the Immgen mononuclear phagocytes dataset (GSE122108) [10, 11], a second reference-based annotation was performed. After generating the UMAP visualisation in Seurat, "FindSubCluster" was used to identify subsets of DCs, monocytes and macrophages among the *H2*$^+$ myeloid cells in the joint. To annotate *Cd2*$^+$ *H2*$^+$ myeloid precursors in the skin, their gene signature in the joint ("FindConservedMarker" with adjusted *P*-values < 0.01, average log FC > 0.5, average pct.2 < 0.2 while excluding ribosomal genes) were used in UCell (2.0.1) [12]. Cells with a UCell score > 0.995 quantile were identified as *Cd2*$^+$ *H2*$^+$ myeloid precursors. To differentiate between the inflammatory and anti-inflammatory potential of the Kaede^{GREEN} and Kaede^{RED} phagocytes, UCell scores for the GO-BP gene sets GO:0050728 - anti-inflammatory response and GO:0050729 - pro-inflammatory response were calculated. Gene sets were extracted from org.Mm.eg.db (3.15.0) [13] using the AnnotationDbi's (version 1.66.0) [14] "select" function. UCell scores were then compared separately between each cluster using the two-sided Wilcoxon rank-sum test and *P*-values were corrected for multiple testing using the Benjamini and Hochberg (BH) method. For visualisation logFC was scaled using the "scale" function in R without centring. Data were exported as *.h5ad for subsequent analyses in python using seuratDisk's (0.0.0.9015, https://github.com/mojaveazure/seurat-disk) "SaveH5Seurat" and "convert" functions. Kaede^{RED} enriched subclusters were selected by modelling the Kaede^{RED} ratios using a quasibinomial generalised linear model GLM ("glm" function in stats R) due to

overdispersion in the ratios (dispersion > 3,5; calculated by first fitting a binomial model and using the following formula: "sum(residuals(fit, type = "deviance")^2) / fit$df.residual"). To account for the sensitivity to small total cell numbers, which can artificially inflate ratios, log(total) cell population in each cluster was included as a fixed effect (final formula: "cluster + strain + log(total)"). Sublining fibroblasts were selected as the baseline for comparison due to their stable Kaede^{RED} ratio and high total cell number. Resulted *P*-values were corrected for multiple testing using BH method.

Post-processing of mesenchymal clusters (mouse)

Harmony integration over both strains was performed for fibroblasts after log-normalisation and regression of unwanted sources of variation such as mitochondrial gene distribution and cell cycle. The top 30 Harmony components were used for clustering and visualisation. The clusters identified by Seurat (top 30 Harmony components, resolution = 1) were manually annotated based on their marker gene expression. The subset of fibroblasts with the highest transcriptional differences between the strains was identified by first identifying the fibroblast-specific differentially expressed genes (DEGs) using "FindMarkers" in Seurat between strains on the entire joint dataset excluding fibroblasts, as well as on fibroblasts only, and then by subtracting the non-fibroblast significant (adjusted *P*-value < 0.01) genes from the significant fibroblasts-specific genes (adjusted *P*-value < 0.01) upregulated in C57BL/6. Finally, UCell score was calculated for these DEGs and compared among different fibroblast clusters by Seurat's "RidgePlot".

Differential abundance analysis

scCODA (0.1.9) [15] was used for differential abundance analysis to account for the compositionality issue in scRNAseq cluster frequencies [16]. In all cases scCODA "CompositionalAnalysis" was performed using default parameters in python (3.8.0). In human synovial myeloid scRNAseq datasets FOLR2^{high} LYVE1^{+} macrophage cluster was chosen as

the reference clusters as we expected no major change in abundance for these resident cell population [7]. Sex and age (Z-normalized) was accounted for, and the FDR threshold was increased gradually to 0.4 as first credible effect was observed. In human skin dataset reference was chosen automatically by the scCODA and sex was accounted for, and FDR threshold was set to 0.05. In human PBMC dataset reference was set automatically by scCODA and FDR threshold was set 0.05. Additionally edgeR [17] differential abundance test on cluster quantities was performed using diffcyt's (1.22.0) [18] "testDA_edgeR" with default parameters. Neighbourhood-based differential abundance was performed using MiloR (1.10.0) [19] by creating the neighbourhood graph using "buildGraph" ("k" = 20 and "d" = 30, with Harmony components or in case of scVI integration using scVI embeddings), "makeNhoods" with "prop" = 0.1, "countCells" with the biological replicate variable as the samples, and "testNhoods" with "fdr.weighting" set to "neighbour-distance". For visualisation using "plotDAbeeswarm", Spatial FDR α was set to $\alpha < 0.05$. For mouse fibroblasts, MELD (1.0.0 on Python 3.8.8) [20] was used for differential composition analysis. Harmony components were used to generate the graph using the "Graph" function of graphtools (1.5.2, https://github.com/KrishnaswamyLab/graphtools), which was the input to the "fit_transform" function of MELD to calculate the strain-associated densities. These densities were L1 normalised to calculate the strain-associated relative likelihoods for each individual cell, and visualised per fibroblast subcluster.

RNA velocity and CellRank

To generate the spliced and unspliced RNA counts, Velocyto workflow (0.17.17 on Python 3.10.2) [21] for droplet-based 10x Chromium libraries was used. RNA velocities were estimated using scVelo (0.2.4 on Python 3.9.10) [22] in the dynamic mode with default parameters with the following sequence of functions: "scv.pp.moments", "scv.tl.recover_dynamics", "scv.tl.velocity" and "scv.tl.velocity_graph". CellRank (2.0.2) [23] was used to create a transition

matrix by equally combining the velocity vector and the connectivity kernel. After initialising the generalised Perron cluster analysis (GPCCA) [24] estimator, the "fit" function was applied to the combined kernel with n_states between 5 and 13, using the reference-based clustering annotations to identify the optimal macrostates. The "predict_initial_states" function was used default parameters to determine the initial macrostates. To predict the terminal states, the "predict_terminal_states" function was used with the method set to "top_n" and n_states set to 1. The "recover_latent_time" function was used for pseudotime estimation. To visualise the coarse-grained transition matrix, “plot_coarse_T” was used with “order” = “incoming”.

Trajectory differential expression analysis

Trajectory differential expression analysis was performed on the Kaede^{RED} myeloid subset using TradeSeq (1.10.0) [25]. Using the latent time estimated by CellRank as the pseudotime, with the binarized strain considered as the “cellWeights” parameter, the “fitGam" function was applied to the SCT-corrected counts of the highly variable genes to produce negative binomial generalised additive model (NB-GAM) smooth expression values for each strain along the differentiation. To find the differentially expressed genes between strains along the pseudotime “diffEndTest” was implemented. Genes with *P*-value < 0.01 and according to the “cellWeights” parameters, a positive logFC, were assigned to BALB/c, while genes with a negative logFC were assigned to C57BL/6. To generate a smoothed expression of the overall gene signature for each strain, UCell scores were generated with these strain-specific genes and fitted to NB-GAM using TradeSeq’s “fitGam”. “PlotSmoothers” was used to plot the smoothed expression over pseudotime. The strain-specific genes were also used as input to gene ontology functional overrepresentation analysis (see below).

Gene Ontology Overrepresentation analysis

For all functional enrichment analysis the same setting was used for the overrepresentation analysis using the ClusterProfiler (4.4.4) [26] “enrichGO” function with background set to NULL,

using the org.Mm.eg.db (3.15.0) [13] dataset, ontology set to GO-BP and with "pAdjustMethod" = BH, "pvalueCutoff" = 0.01 and "qvalueCutoff" = 0.05. If two or more clusters are compared, then "compareCluster" was used with "fun" set to "enrichGO". The selection of genes of interest was performed using either Seurat's "FindConservedMarkers", when conserved markers were considered among strains, or "FindMarker". The selection criteria for significant genes were adjusted *P*-value < 0.01 and average |logFC| > 0.5.

Ligand-receptor interaction analysis

Ligand-receptor interaction analysis was performed using CellChat (1.5.0) [27] on the SCT normalised assay including all annotated cell types separately for each strain. To detect enriched signalling pathways, the "rankNet" function was used after merging the CellChat objects, followed by row normalisation. Signalling pathways with more than 50% relative contribution in each strain were selected for downstream visualisation. Interaction probabilities were calculated for all annotated subclusters, but for visualisation their probability was summarised by grouping major cell types. In case of T cell interactions with other cell types, the interaction analysis was performed on BALB/c Kaede^{tg} mice scRNAseq dataset and all pathways were included.

Quality control and processing of single-cell RNA-seq libraries (human)

A public dataset of human synovial scRNAseq dataset (E-MTAB-11791 [28]) and the in-house healthy human synovial libraries (E-MTAB-14339) were subjected to a quality control to exclude low quality events by individually examining the distribution of quality criteria for each dataset, including the number of UMIs >= 1,400 / 1,000 number of genes detected >= 700 / 800, and mitoRatio < 0.4 / 0.3 and complexity (log10(genes/UMI) > 0.80 / 0.83, respectively. Genes expressed in fewer than 10 cells were excluded. Doublets were detected and removed using scDblFinder (1.13.12) [29] with default parameters. To overcome batch effects, the scVI (1.1.1, Python 3.9.18) [30, 31] integration was used. Scanpy (1.9.8) [32] log-normalisation and

variable gene detection (seurat_v3) were performed on the top 3,000 variable genes. The scVI model was defined with categorical batch variables (library, dataset origin) and their continuous covariates (mitoRatio, cell cycle score) and trained with four layers and 30 latent layers. Clustering was performed using the Leiden algorithm in scanpy and clusters were manually annotated based on their marker gene expression. Myeloid cells were subset and subjected to a repeated scVI workflow with manual annotation to identify subclusters. Alra imputation [33] (as part of SeuratWrapper 0.3.4, https://github.com/satijalab/seurat-wrappers) was performed and the absence of T, B and DC markers in the *CD2*-expressing myeloid cluster was confirmed to exclude any *CD2*-expressing contaminating events originating from previously undetected DCs or lymphocyte doublets. Myeloid subclusters from PsA and healthy samples were subjected to scANVI reference mapping as described below.

A second public dataset of human synovial scRNAseq dataset (E-MTAB-8322 [7]) was analysed separately. Quality control was performed in Seurat by removing cells with number of UMIs < 800, number of detected genes < 300, complexity < 0.8, mitoRatio > 0.01 and < 0.2. Genes expressed in fewer than 10 cells were excluded. Doublets were detected and removed using scDblFinder as described above. Harmony integration was performed across libraries after log-normalisation and regression of cell cycle scores followed by Seurat clustering (resolution = 0.3). Alra imputation was performed for gene expression visualisation as described above. E-MTAB-8322 only captured *CD1C*$^{+}$ and *CCR7*$^{+}$ DCs due to technical restrictions in cell isolation. Myeloid cells were subset and subjected to scANVI reference mapping together with the myeloid subclusters from PsA and healthy synovia as described below, while excluding the undetected subsets of DCs.

Filtered and annotated data were retrieved from a public scRNAseq dataset of human skin from healthy individuals and patients with psoriasis and AD (E-MTAB-8142 [34]). Myeloid cells were subset and subjected to scANVI reference mapping together with the myeloid subclusters from

PsA and healthy synovia as described below. Differential abundance analysis on the human skin scRNAseq dataset was performed as described above.

Filtered and annotated data were retrieved from a public scRNAseq/HTOseq dataset of human PBMCs from healthy individuals and patients with psoriasis, PsA and PsX (GSE194315 [35]). Datasets without HTO immunophenotyping were excluded. Myeloid cells were separately subjected to scVI integration as described above (2,000 variables genes, batch variable (library), continuous covariates (mitoRatio, heat shock gene score). Clustering using the Leiden algorithm in scanpy identified the original annotated clusters together with two doublet clusters co-expressing typical myeloid cell markers and either those for T and NK cells or B cells, which were excluded. Myeloid cells were down-sampled to 25 %, subset and subjected to scANVI reference mapping together with the myeloid subclusters from PsA and healthy synovia as described below. Differential abundance analysis on the human PBMC scRNAseq dataset was performed as described above.

scRNAseq datasets from matched skin and joint biopsies of three patients were analysed using Seurat (5.2.1) [36]. Cells were filtered based on the following QC criteria: number of UMIs > 1800, number of genes > 1000, mitochondrial gene ratio between 0.008 and 0.22, and erythrocyte gene ratio (HBA and HBB) < 0.002. Genes expressed in fewer than 10 cells or those without annotation were removed. Harmony (1.2.3) [3] was used for integration. Clustering was done using Seurat "FindCluster" (**Figures S1a and c**). Within each major cluster, subclusters were identified using Seurat's "FindSubClusters" function. Subclusters of myeloid cells were annotated based on their top upregulated genes. Reference mapping of myeloid cells in skin and joint was performed using scANVI, following steps described in "Cross-tissue reference mapping" section below and based on our previously annotated synovial myeloid scRNAseq dataset (**Figures S1b and d**). Plasmacytoid dendritic cells (pDCs) were not included in the skin scANVI analysis due to their absence in the skin samples.

Cross-tissue and -species integration and reference mapping (scANVI)

Reference mapping was performed using scANVI according to the scvi-tools (1.1.1) [32] guidelines to map annotations between the pairs of reference/query datasets described above. The workflow included merging the query and reference datasets, Scanpy log-normalisation and variable feature selection (3,000 variable features using "seurat_v3"), scVI model construction (4 layers, 30 hidden layers, with sample and organism or tissue as categorical batch variables and predetermined continuous covariates), preparation of missing labels on the merged object by setting the query datasets cluster labels to "unknown", scANVI model setup and training with 100 max epochs and 100 samples per label, and finally the scANVI "predict" function. In order to create a co-occurrence heat map, a matrix was created by evaluating the ratio of mapped annotations in each of the originally annotated clusters of the query dataset using the following script: query_metadata.groupby([‘origninal_label’, ‘scANVI_label’]).size().reset_index(name='total').groupby(‘origninal_label’)['total'].transform(lambda x: x / x.sum()). A scaling was performed on the ratios using "sklearn.preprocessing" "scale" function for visualisation. In the case of cross-species mapping, genes in the mouse dataset were translated into human orthologs using the "translate" function in mousipy (0.1.5, https://github.com/stefanpeidli).

Single cell correlation analysis

To asses gene correlations in scRNAseq datasets of human PsA and healthy fibroblasts, we created meta cells using hdWGCNA (0.3.00) [37] "MetacellsByGroups" function on the Alra imputed expressions. Correlation was measured between expression levels in the meta cells with Pearson analysis.

Analysis of MAESTER libraries

Mitochondrial variant calling

Mitochondrial variant calling was performed following the steps in the MAESTER protocol [38] and scripts provided in the original publication [39]. The raw sequencing files were first assembled using the "Assemble_fastq.R" script. Then, 24 nucleotides were trimmed from the 5′ end using homerTools (v4.11.1) [40]. Reads were aligned to the GRCh38-2024-A reference genome using STAR (v2.7.3a) [41], and mitochondrial chromosome reads were extracted using samtools (v1.10) [42]. Barcode and UMI sequences were moved from the read ID into the BAM file using the "Tag_CB_UMI.sh" script. Variant calling was performed using maegatk (v0.2.0 https://github.com/caleblareau/maegatk) with the command: "maegatk bcall -i SAMPLE_chrM.10x.bam -z --snake-stdout -mr 3", as recommended by the protocol. The skin sample from patient 2 was randomly down sampled to 30 percent to improve processing speed. Mitochondrial DNA coverage was visualized using the script provided in the original MAESTER manuscript [39] "MT_coverage.R" (**Figures S4a**).

Identification of informative mitochondrial variants

Following the MAESTER protocol, we identified informative mitochondrial variants specific to each subcluster of cells in the skin and joint samples, separately for each patient. Clusters with fewer than 50 cells were excluded due to imbalance between samples and possible instability in variant identification. For each variant and subcluster, we calculated quality metrics using provided scripts "8.1_MGH252_voi.R", including mean quality (mean base quality score across all cells where a variant was observed on either strand), mean and 99th percentile allele frequency (AF) or heteroplasmy, and mean coverage.

Variants with quality scores below 30 were filtered out. Variants were considered informative if they had mean coverage >5, 99th percentile heteroplasmy >5% (i.e., at least 1% of the cells have a heteroplasmy level exceeding 5% for this variant) and if their 99th percentile heteroplasmy was 5 times higher than in all other clusters, except those of the same lineage

(allowing us to keep shared variants that might be present across related cell types). To confirm the cluster specificity of these variants, we calculated the mean variant allele frequency (VAF) over all the variants for each cluster and visualized the results using a heatmap (**Figures S4b**). High values on the diagonal indicated that the variants were specific to their assigned clusters.

Cross-tissue mitochondrial variant comparison

We intersected informative variants between each pair of subclusters across skin and joint samples. For each major cell type shared variants were defined as the union of common variants found across all associated subclusters. In the joint myeloid subclusters, variant identification was performed using the manual annotation in order to avoid potential bias introduced by label transfer as opposed to manual clustering. However, for the final visualization, we used the scANVI labels. For each scANVI-defined cluster, variants were aggregated based on their corresponding manual annotations (**Figures S1b**). Statistical significance was tested using a one-vs-rest two-sided Wilcoxon rank-sum test, with p-values adjusted using the BH correction.

Mitochondrial variants identification robustness analysis

Given the sensitivity of informative variant identification to threshold settings, as well as the overall differences in data quality between skin and joint samples, possibly due to ongoing inflammation in the skin, we repeated the common variant identification process across 1,000 iterations using varying thresholds. In each iteration, we changed the thresholds for mean coverage (from 1 to 10), the skin 99th percentile heteroplasmy (from 1 % to 10 %), and the joint 99th percentile heteroplasmy (from 1 % to 10 %). For each cluster or subcluster in each iteration, we recorded the median rate of shared variants across the three patients, as well as the rank of this rate compared to other clusters. The results were visualized using boxplots (for medians) and heatmaps (for ranks).

Graph-based analysis of mitochondrial variant sharing

Graph analysis within the myeloid subclusters in the joint was performed using igraph (2.1.4) [43], where each subcluster was represented as a node. Edges between nodes were weighted based

on the number of shared variants between each pair of subclusters (**Figures S1e)**. This was done both including and excluding skin-joint common variants. Graph strength for each node was calculated using the "strength" function from the igraph. Strength values were then compared using a one-vs-rest two-sided Wilcoxon rank-sum test, and *P*-values were adjusted using the BH method.

Supplementary Figures

Supplementary Data Figure 1

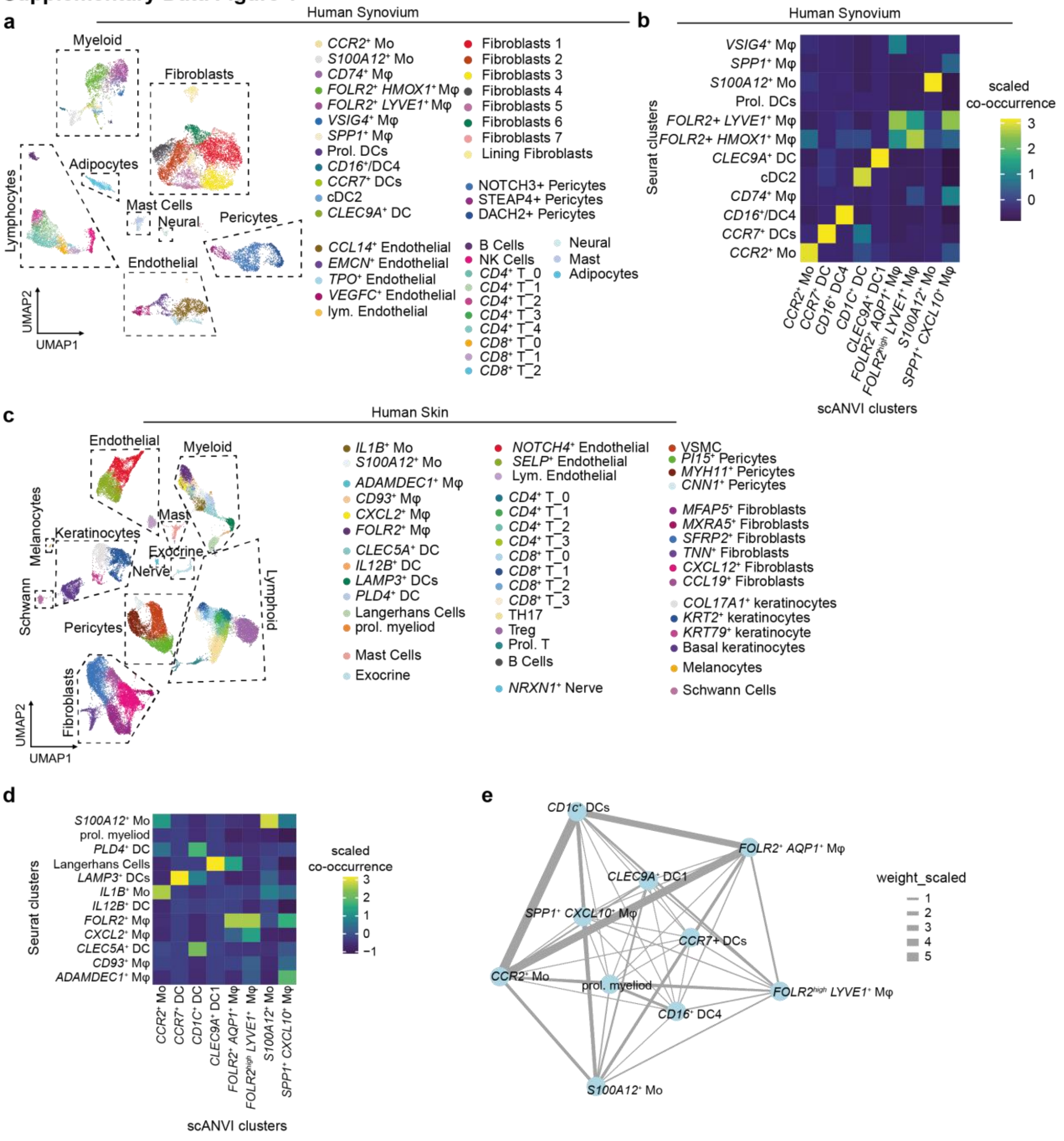

Supplementary Figure 1:

(**a**) UMAP plot of the identified clusters in the integrated dataset of synovia from psoriasis at risk and PsA patients used in the mitochondrial variant enrichment experiment. Psoriasis at risk $N = 2$, PsA $N = 1$. (**b**) Heatmap of co-occurrence of Seurat identified clusters and scANVI mapped labels in the scRNAseq datasets of synovia used in the mitochondrial enrichment

analysis. scANVI reference mapping was done based on the dataset from **Figure 3a**. (**c**) UMAP plot of the identified clusters in the integrated dataset of skin from psoriasis at risk and PsA patients used in the mitochondrial variant enrichment experiment. Psoriasis at risk $N = 2$, PsA $N = 1$. (**d**) Heatmap of co-occurrence of Seurat identified clusters and scANVI mapped labels in the scRNAseq datasets of skin used in the mitochondrial enrichment analysis. scANVI reference mapping was done based on the dataset from **Figure 3a**. (**e**) Visualization of the shared variant graph among the myeloid subclusters in the synovium. Nodes represent each myeloid subcluster. Edge thickness corresponds to the scaled mean number of shared variants between two nodes.

Supplementary Data Figure 2

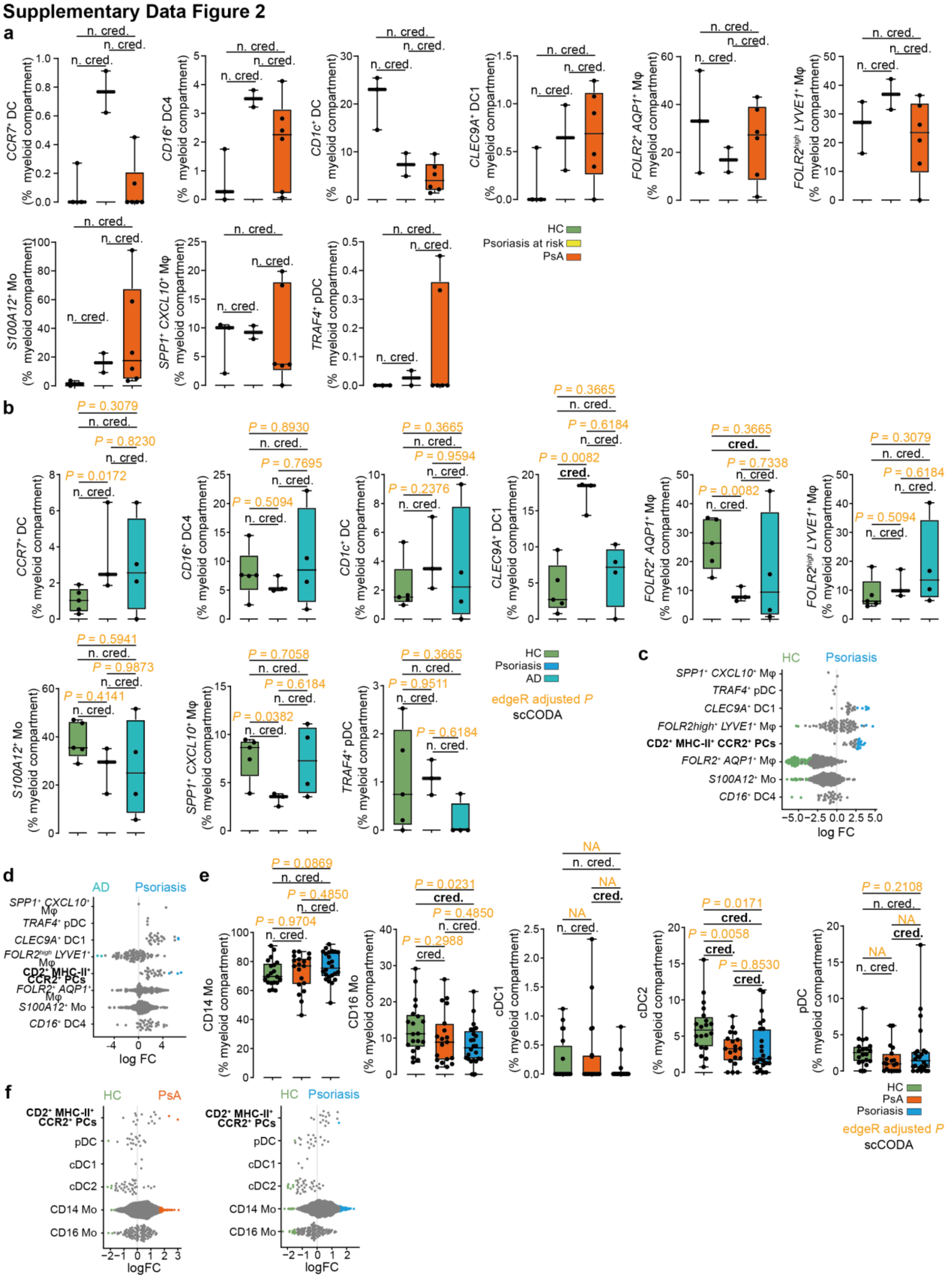

Supplementary Figure 2:

(**a**) Proportion of different myeloid cell populations in psoriasis at risk and PsA compared to healthy in the scRNAseq dataset of human synovial tissue. D2^{+} MHC-II^{+} CCR2^{+} myeloid

precursors are shown in **Figure 3 f**. Graph shows median, quartiles and min-max.; psoriasis at risk *N* = 2; PsA *N* = 5, healthy subjects (HC) *N* = 3; Statistically credible (cred.) and non-credible (n. cred.) changes in abundance were identified using scCODA. (**b**) Proportion of different myeloid cell populations in healthy (HC), psoriasis and AD in scRNAseq dataset of human skin (E-MTAB-8142). $CD2^+$ MHC-II^+ $CCR2^+$ myeloid precursors are shown in **Figure 3 g**. Graph shows median, quartiles and min-max.; healthy *N* = 5; psoriasis *N* = 3, AD *N* = 4; *P*-values were calculated by edgeR's differential abundance test and corrected for multiple testing using the BH-method (orange); statistically credible (cred.) and non-credible (n. cred.) changes in abundance were identified using scCODA (black). (**c**) Differential abundance of Milo neighbourhoods in psoriatic versus healthy human skin shown as a bee swarm plot. Significantly enriched (spatial false discovery rate (FDR) < 0.05, positive fold changes (FCs), blue) or depleted (FDR < 0.05, negative FCs, green) neighbourhoods among the scANVI mapped clusters from the human synovium are highlighted. (**d**) Differential abundance of Milo neighbourhoods in atopic dermatitis (AD) versus psoriasis in the skin scRNAseq shown as a bee swarm plot. Significantly enriched (spatial false discovery rate (FDR) < 0.05, positive FCs, blue) or depleted (FDR < 0.05, negative FCs, cyan) neighbourhoods among the scANVI mapped clusters from the human synovium are highlighted. (**e**) Proportion of subclusters of myeloid cells along in healthy compared to PsA and psoriasis in CITEseq dataset of human PBMC (GSE194315). scANVI identified $CD2^+$ MHC-II^+ $CCR2^+$ myeloid precursors are shown in **Figure 3i.** Graphs show median, quartiles and min–max; healthy subjects *N* = 29, PsA *N* = 20, psoriasis *N* = 33; *P*-values were calculated by edgeR differential abundance test and corrected for multiple testing using the BH-method (orange); statistically credible (cred.) and non-credible (n. cred.) changes in abundance were identified using scCODA (black). (**f**) Differential abundance of Milo neighbourhoods in PBMCs from healthy donors or patients with PsA (left) or psoriasis (right) shown as a bee swarm plot. Significantly enriched (FDR < 0.05, positive FCs, orange / blue) or depleted (FDR < 0.05, negative FCs, green) neighbourhoods

among the original annotation as well as the scANVI identified $CD2^+$ MHC-II^+ $CCR2^+$ myeloid precursors are highlighted.

Supplementary Data Figure 3

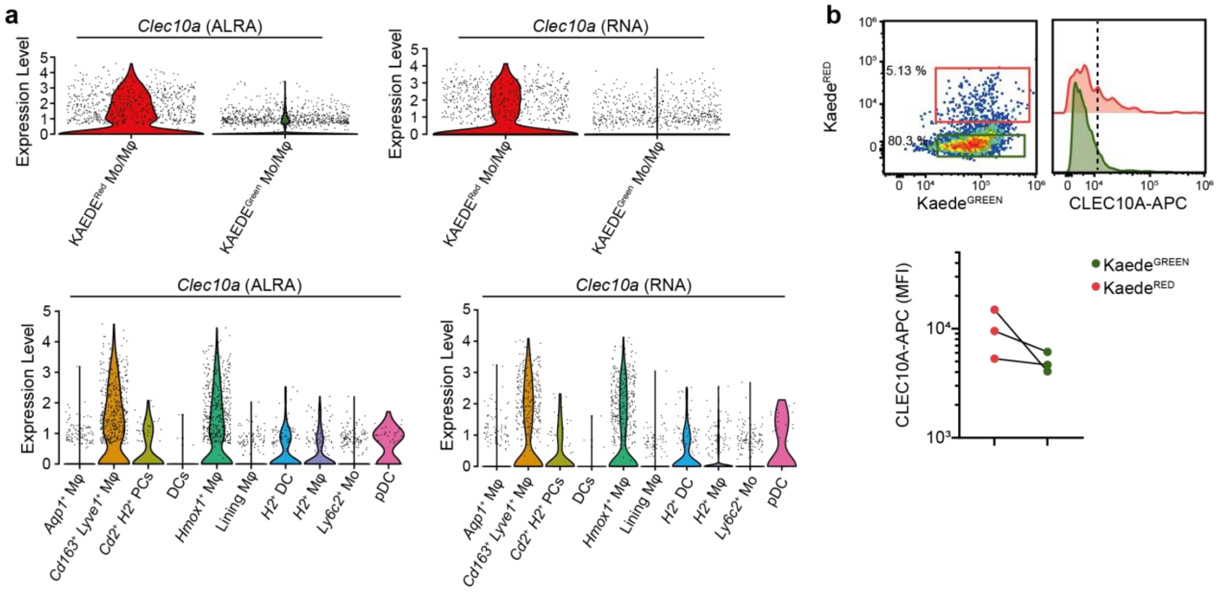

Supplementary Figure 3:

(**a**) ALRA-imputed (left) and RNA (right) expression of *Clec10a* in Kaede^{RED} and Kaede^{GREEN} myeloid cells (top) and among the subclusters of myeloid cells (bottom) the joint of the scRNAseq dataset from Kaede^{tg} mice on BALB/c and C57BL/6 background on day 7 of the IL-23OE. (**b**) Representative flow cytometry plots of CLEC10A expression on Kaede^{RED} versus Kaede^{GREEN} viable CD45$^+$ CD11b$^+$ Ly6G$^-$ CD11c$^-$ CCR7$^-$ CD3$^-$ B220$^-$ cells in the joint on day 7 of the IL-23OE in Kaede^{tg} animals. Quantification of geometric median fluorescence intensity in Kaede^{RED} versus Kaede^{GREEN}.

Supplementary Data Figure 4

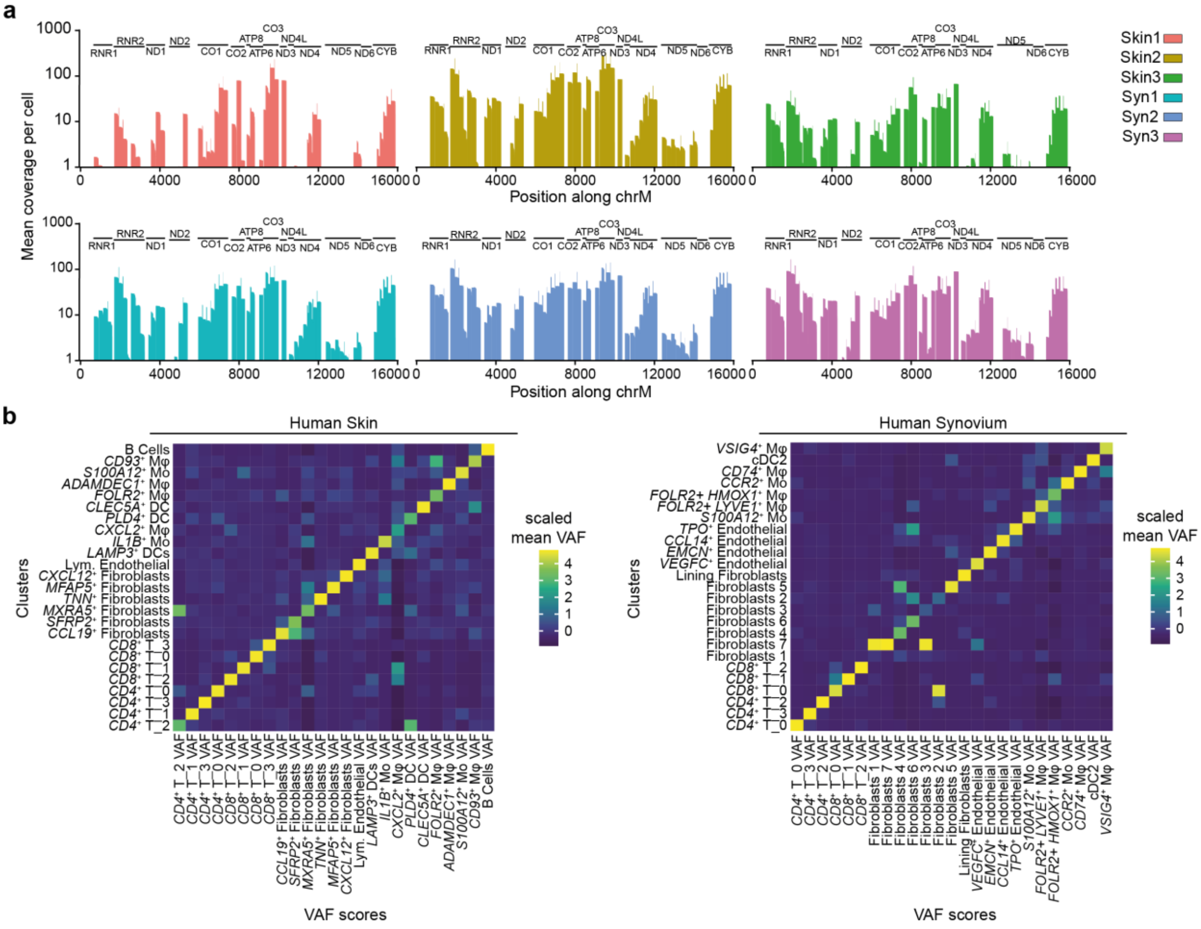

Supplementary Figure 4:

(**a**) Mean coverage over the mitochondrial genome in the MAESTER mitochondrial sequencing libraries for each sample. (**b**) Heatmap of mean variant allele frequencies (VAF) of the informative mitochondrial variants across subclusters for the skin (left) and the synovia (right) dataset.

Supplementary Tables

Supplementary Table S1: Synovial tissue patient characteristics

	**Psoriasis (*N* = 8)**	**PsA (*N* = 6)**	***P*-value**
**Female, n (%)**	4 (50)	2 (33)	0.6270
**Age, mean (SD)**	45 (12)	59.7 (8)	0.0216
**Psoriasis disease duration years, mean (SD)**	18.1 (13.7)	11 (9.6)	0.4908
**PsA disease duration years, mean (SD)**	N/A	1.2 (0.5)	N/A
**DAPSA, mean (SD)**	N/A	22.7 (6.3)	N/A
**Krenn Score, mean (SD)**	1.3 (0.7)	4.2 (1.6)	0.0087

N/A = not applicable; PsA = Psoriatic arthritis; SD = standard deviation. *P*-values were calculated by non-parametric t-test.

Supplementary Table S2: PBMCs patient characteristics

	**PsA (*N* = 29)**
**Female, n (%)**	15 (51.7)
**Age, mean (SD)**	53.2 (12)
**Disease duration years, mean (SD)**	6.4 (4.9)

PBMCs = peripheral blood mononuclear cells; PsA = Psoriatic arthritis; SD = standard deviation.

Supplementary Table S3: Resource table

REAGENT or RESOURCE	SOURCE	IDENTIFIER
**Antibodies**		
Brilliant Violet 510(TM) anti-human CD3	BioLegend	Cat# 300448, RRID:AB_2563468
Brilliant Violet 510(TM) anti-human CD19	BioLegend	Cat# 302242, RRID:AB_2561668
Brilliant Violet 510(TM) anti-human CD56 (NCAM)	BioLegend	Cat# 318340, RRID:AB_2561944
Brilliant Violet 421(TM) anti-human CD11c	BioLegend	Cat# 301627, RRID:AB_10898313
APC anti-human CD2	BioLegend	Cat# 300214, RRID:AB_10895925
APC/Cyanine7 anti-human HLA-DR	BioLegend	Cat# 307618, RRID:AB_493586
PE/Cyanine7 anti-human CD14	BioLegend	Cat# 301814, RRID:AB_389353
PerCP/Cyanine5.5 anti-human CD45	BioLegend	Cat# 304028, RRID:AB_893338
PE anti-human CD200R	BioLegend	Cat# 329306, RRID:AB_2074200
PE anti-human CD192 (CCR2)	BioLegend	Cat# 357206, RRID:AB_2562059
PE anti-human CD1c	BioLegend	Cat# 331506, RRID:AB_1088999
PE anti-human CD123	BioLegend	Cat# 306006, RRID:AB_314580
PE anti-human CD209 (DC-SIGN)	BioLegend	Cat# 330106, RRID:AB_1134052
PE Mouse IgG1, κ Isotype Ctrl (FC)	BioLegend	Cat# 400114, N/A
PE Mouse IgG2a, κ Isotype Ctrl (FC)	BioLegend	Cat# 400213, RRID:AB_2800438
FITC anti-mouse CD45	BioLegend	Cat# 103108, RRID:AB_312973
PE anti-mouse Ly-6G	BioLegend	Cat# 127608, RRID:AB_1186099
Brilliant Violet 421(TM) anti-mouse/human CD11b	BioLegend	Cat# 101236, RRID:AB_11203704
APC anti-mouse CD2	BioLegend	Cat# 100112, RRID:AB_2563090
Brilliant Violet 510(TM) anti-mouse I-A/I-E	BioLegend	Cat# 107636, RRID:AB_2734168
PE/Cyanine7 anti-mouse CD3ε	BioLegend	Cat# 100320, RRID:AB_312685
PE/Cyanine7 anti-mouse/human CD45R/B220	BioLegend	Cat# 103222, RRID:AB_313005

PerCP/Cyanine5.5 anti-mouse CD11c	BioLegend	Cat# 117328, RRID:AB_2129641
Brilliant Violet 421(TM) anti-mouse CD140a	BioLegend	Cat# 135923, RRID:AB_2814036
PE/Cyanine7 anti-mouse CD45	BioLegend	Cat# 103114, RRID:AB_312979
Brilliant Violet 510(TM) anti-mouse CD90.2 (Thy1.2)	BioLegend	Cat# 105335, RRID:AB_2566587
PerCP/Cyanine5.5 anti-mouse Podoplanin	BioLegend	Cat# 127422, RRID:AB_2814016
PE/Cyanine7 anti-mouse CD31	BioLegend	Cat# 102418, RRID:AB_830757
FITC anti-human/mouse CD49f	BioLegend	Cat# 313606, RRID:AB_345300
APC anti-mouse CD200 (OX2)	BioLegend	Cat# 123810, RRID:AB_10900447
APC anti-mouse CD45	BioLegend	Cat# 103112, RRID:AB_312977
APC/Cyanine7 anti-mouse/human CD11b	BioLegend	Cat# 101226, RRID:AB_830642
Brilliant Violet 421(TM) anti-mouse Ly-6G	BioLegend	Cat# 127628, RRID:AB_2562567
TotalSeq(TM)-B0301 anti-mouse Hashtag 1	BioLegend	Cat# 155831, RRID:AB_2814067
TotalSeq(TM)-B0302 anti-mouse Hashtag 2	BioLegend	Cat# 155833, RRID:AB_2814068
TotalSeq(TM)-B0987 anti-Allophycocyanin (APC)	BioLegend	Cat# 408009, RRID:AB_2894695
TotalSeq(TM)-B0303 anti-mouse Hashtag 3	BioLegend	Cat# 155835, RRID:AB_2814069
TotalSeq(TM)-B0304 anti-mouse Hashtag 4	BioLegend	Cat# 155837, RRID:AB_2814070
TotalSeq(TM)-B0305 anti-mouse Hashtag 5	BioLegend	Cat# 155839, RRID:AB_2814071
Purified anti-human Mast Cell Tryptase	BioLegend	Cat# 369402, RRID:AB_2566541
Mouse Anti-Actin, alpha-Smooth Muscle Monoclonal Antibody, Unconjugated, Clone 1A4	Sigma Aldrich	Cat# A5228, RRID:AB_262054
Pan-cadherin Polyclonal Antibody	Thermo Fisher	Cat# 71-7100, RRID:AB_2533992
Anti-beta 2 Microglobulin antibody [EPR21752-214] - BSA and Azide free	abcam	Cat# ab237032, N/A
CCR2 Polyclonal Antibody	Thermo Fisher	Cat# PA5-23037, RRID:AB_11153363
CD20 Monoclonal Antibody (L26), eBioscience	Thermo Fisher	Cat# 14-0202-82, RRID:AB_10734340
Anti-CD16 antibody [EPR16784] - C-terminal	abcam	Cat# ab198507, N/A
Anti-Human CD14 (EPR3653)-144Nd	Standard Biotools	Cat# 3144025D, RRID:AB_2924314

Anti-CD2 antibody [EPR6451] - BSA and Azide free	abcam	Cat# ab248400, N/A
CD8 Antibody (C8/144B)	Novus Biologicals	Cat# NBP2-34588, N/A
CD68 Monoclonal Antibody (KP1), eBioscience	Thermo Fisher	Cat# 14-0688-82, RRID:AB_11151139
Anti-CD161 antibody [EPR26340-6] (BSA and Azide free)	abcam	Cat# ab302565, N/A
Recombinant Anti-CD11b antibody [EPR1344] - BSA and Azide free	abcam	Cat# ab209970, RRID:AB_2915959
Anti-MERTK antibody [Y323] - BSA and Azide free	abcam	Cat# ab271851, N/A
Recombinant Anti-CD31 antibody [EPR3094] - BSA and Azide free	abcam	Cat# ab207090, RRID:AB_2889382
CD45 (Intracellular Domain) (D9M8I) XP Rabbit mAb (BSA and Azide Free)	Cell Signaling	Cat# 47937, RRID:AB_2922773
Mouse Anti-Human MRC1 Monoclonal Antibody, Unconjugated, Clone 5C11	Abnova	Cat# H00004360-M02, RRID:AB_565555
Recombinant Anti-CD11c antibody [EP1347Y] - BSA and Azide free	abcam	Cat# ab216655, RRID:AB_2864379
FOXP3 Monoclonal Antibody (PCH101), eBioscience	Thermo Fisher	Cat# 14-4776-82, RRID:AB_467554
Recombinant Anti-CD4 antibody [EPR6855] - BSA and Azide free	abcam	Cat# ab181724, RRID:AB_2864377
Anti-S100A12/CGRP antibody [EPR23677-111] - BSA and Azide free	abcam	Cat# ab273051, N/A
Goat Anti-Human Dkk-3 Polyclonal antibody, Unconjugated	R&D systems	Cat# AF1118, RRID:AB_354610
Anti-LYVE1 antibody [RM1067] - BSA and Azide free	abcam	Cat# ab314242, N/A
Anti-CD200R antibody [EPR21232]	abcam	Cat# ab232941, N/A
Purified anti-human CD15 (SSEA-1)	BioLegend	Cat# 323002, RRID:AB_756008
Purified (azide-free) anti-Podoplanin (Lymphatic Endothelial Marker)	BioLegend	Cat# 916606, RRID:AB_2565820
Anti-IL3RA/CD123 antibody [EPR23188-72] - BSA and Azide free	abcam	Cat# ab269871, N/A
CD163 Monoclonal Antibody (EDHu-1)	Thermo Fisher	Cat# MA1-82342, RRID:AB_2275716
Human CD90/Thy1 Affinity Purified Polyclonal Ab	R&D systems	Cat# AF2067, RRID:AB_11127215
Anti-CD127 antibody [EPR2955(2)] - BSA and Azide free	abcam	Cat# ab240225, RRID:AB_3094461
Purified anti-human HLA-DR	BioLegend	Cat# 327002, RRID:AB_893582
CD3ε (D7A6E™) XP Rabbit mAb (BSA and Azide Free)	Cell Signaling	Cat# 24581, RRID:AB_2922776
CD200 (E5I9V) XP Rabbit mAb (BSA and Azide Free)	Cell Signaling	Cat# #24510, N/A
IL23R Polyclonal Antibody	Thermo Fisher	Cat# PA5-102441, RRID:AB_2851846

Sheep Anti-Human Fap Affinity purified Polyclonal antibody, Unconjugated	R&D systems	Cat# AF3715, RRID:AB_2102369
Recombinant Anti-CD55 antibody [EPR6689]	abcam	Cat# ab133684, RRID:AB_2801387
CD1C Monoclonal Antibody (OTI2F4), TrueMAB™	Thermo Fisher	Cat# CF505411, N/A
Anti-Histone 3 (D1H2)-176Yb	Standard Biotools	Cat# 3176023D, RRID:AB_2811058
IMC Cell Segmentation Kit	Standard Biotools	Cat# TIS-00001, N/A
InVivoMAb anti-mouse CD200 (OX2)	BioXCell	Cat# BE0299, RRID:AB_2687821
Purified anti-mouse CD200 (OX2)	BioLegend	Cat# 123802, RRID:AB_1236498
Ultra-LEAF™ Purified Rat IgG2a, κ Isotype Ctrl	BioLegend	Cat# 400543, RRID:AB_11148951
Brilliant Violet 421™ anti-mouse CD45 Antibody	BioLegend	Cat# 103134, RRID:AB_2562559
FITC anti-mouse/human CD11b Antibody	BioLegend	Cat# 101206, RRID:AB_312789
Brilliant Violet 510™ anti-mouse I-A/I-E Antibody	BioLegend	Cat# 107636, RRID:AB_2734168
APC anti-mouse CD301 (MGL1/MGL2) Antibody	BioLegend	Cat# 145708, RRID:AB_2562942
PE anti-mouse CD2 Antibody	BioLegend	Cat# 100108, RRID:AB_2073690
APC/Cyanine7 anti-mouse CD11c	BioLegend	Cat# 117324, RRID:AB_830649
APC/Cyanine7 anti-mouse CD3 Antibody	BioLegend	Cat# 100222, RRID:AB_2242784
APC/Cyanine7 anti-mouse/human CD45R/B220 Antibody	BioLegend	Cat# 103224, RRID:AB_313007
PE/Cyanine7 anti-mouse CD197 (CCR7) Antibody	BioLegend	Cat# 120124, RRID:AB_2616688
PerCP/Cyanine5.5 anti-mouse Ly-6G Antibody	BioLegend	Cat# 127616, RRID:AB_1877271
Brilliant Violet 421™ anti-mouse Podoplanin Antibody	BioLegend	Cat# 127423, RRID:AB_2814017
BD OptiBuild™ BV480 Rat Anti-Mouse Ly-6G	BD	Cat# 746448, RRID:AB_2743751
Brilliant Violet 421™ anti-mouse CD140a Antibody	BioLegend	Cat# 135923, RRID:AB_2814036
Brilliant Violet 570™ anti-mouse CD90.2 (Thy1.2) Antibody	BioLegend	Cat# 105329, RRID:AB_10917055
BD OptiBuild™ BV605 Rat Anti-Mouse CD2	BD	Cat# 740338, RRID:AB_2740071
BD OptiBuild™ BV650 Rat Anti-Mouse CD31	BD	Cat# 740483, RRID:AB_2740207
Brilliant Violet 711™ anti-mouse I-A/I-E Antibody	BioLegend	Cat# 107643, RRID:AB_2565976

Brilliant Violet 785™ anti-mouse/human CD45R/B220 Antibody	BioLegend	Cat# 103246, RRID:AB_2563256
FITC anti-mouse CD45 Antibody	BioLegend	Cat# 103108, RRID:AB_312973
PE anti-mouse CD200 (OX2) Antibody	BioLegend	Cat# 123808, RRID:AB_2073942
PerCP/Cyanine5.5 anti-human/mouse CD49f Antibody	BioLegend	Cat# 313618, RRID:AB_2249260
PE/Cyanine7 anti-mouse CD11c Antibody	BioLegend	Cat# 117318, RRID:AB_493568
APC anti-mouse/human CD11b Antibody	BioLegend	Cat# 101212, RRID:AB_312795
APC/Fire™ 810 anti-mouse CD3 Antibody	BioLegend	Cat# 100268, N/A
APC anti-human HLA-DR Antibody	BioLegend	Cat# 307610, RRID:AB_314688
Brilliant Violet 510™ anti-human CD3 Antibody	BioLegend	Cat# 300448, RRID:AB_2563468
FITC anti-human CD8 Antibody	BioLegend	Cat# 344704, RRID:AB_1877178
PerCP/Cyanine5.5 anti-human CD4 Antibody	BioLegend	Cat# 357414, RRID:AB_2565666
Brilliant Violet 421™ anti-human CD192 (CCR2) Antibody	BioLegend	Cat# 357210, RRID:AB_2563463
PE anti-human CD2 Antibody	BioLegend	Cat# 300208, RRID:AB_314032
PE/Cyanine7 anti-human CD1c Antibody	BioLegend	Cat# 331516, RRID:AB_2275574
PE/Cyanine7 anti-human CD123	BioLegend	Cat# 983702, RRID:AB_2749873
Spark PLUS UV395™ anti-human CD19 Antibody	BioLegend	Cat# 302298, N/A
Brilliant Violet 510™ anti-human CD3 Antibody	BioLegend	Cat# 300448, RRID:AB_2563468
PerCP/Cyanine5.5 anti-human CD4 Antibody	BioLegend	Cat# 357414, RRID:AB_2565666
FITC anti-human CD8 Antibody	BioLegend	Cat# 344704, RRID:AB_1877178
PE/Cyanine7 anti-human IL-17A Antibody	BioLegend	Cat# 512315, RRID:AB_2295923
Ultra-LEAF™ Purified anti-mouse CD200R (OX2R) Antibody	BioLegend	Cat# 123918, N/A
**Chemicals, peptides, and recombinant proteins**		
Recombinant Mouse M-CSF (carrier-free)	BioLegend	Cat# 576404
Collagenase D	Merck/ Roche	Cat# 11088866001
DNAse I	Merck/ Roche	Cat# 10104159001
Dispase II	Sigma Aldrich	Cat# D4693-1G
Fixable Viability Stain 510 100ug	BD	Cat# 564406
Fixable Viability Dye eFluor 780	Thermo Fisher	Cat# 65-0865-14
RBC lysis buffer	BioLegend	Cat# 420302

Debris removal solution	Miltenyi Biotec	Cat# 130-109-398
Lymphosep	Biowest	Cat# L0560
Penicillin-Streptomycin	Gibco	Cat# 11548876
Amphotericin B	Gibco	Cat# 11510496
L-Glutamine 200 mM	Sigma Aldrich	Cat# G7513-100ML
Accutase Cell Dissociation Reag., 100 ml	Gibco	Cat# A1110501
FBS	Gibco	Cat# 10270-106
SYBR Select Mastermix	Applied Biosystems	Cat# 4472920
Ir-intercalator	Standard Biotools	Cat# 201192A
rh M-CSF	Biolegend	Cat# 574808
rh IL-2	Biolegend	Cat# 589104
rh TGF-ß1	PeproTech	Cat# 100-21c-10UG
rh IL-6	Biolegend	Cat# 570804
rh IL-1ß	Biolegend	Cat# 579404
rh IL-23	Biolegend	Cat# 574104
Cell Activation Cocktail	Biolegend	Cat# 423301
Brefeldin A Solution (1000x)	Biolegend	Cat# 420601
Monensin Solution (1000x)	Biolegend	Cat# 420701
Imiquimod (Aldara 5%)	Viatris	N/A
**Oligonucleotides**		
beta 2 microglobulin Forward primer 5'-GGTGCTTGTCTCACTGACCG-3' Reverse primer 5'-TTTGAGGGGTTTTCTGGATAGCAT-3'	This paper	N/A
Nos2 Forward primer 5'-GTTCTCAGCCCAACAATACAAGA-3' Reverse primer 5'-GTGGACGGGTCGATGTCAC-3'	This paper	N/A
Il1b Forward primer 5'-GCAACTGTTCCTGAACTCAACTG-3' Reverse primer 5'-TCTTTTGGGGTCCGTCAACTTC-3'	This paper	N/A
Il6 Forward primer 5'-TACCACTTCACAAGTCGGAGGC-3' Reverse primer 5'-CTGCAAGTGCATCATCGTTGTTC-3'	This paper	N/A
Arg1 Forward primer 5'-CTCCAAGCCAAAGTCCTTAGAG-3' Reverse primer 5'-AGGAGCTGTCATTAGGGACATC-3'	This paper	N/A
Il10 Forward primer 5'-CCCTGGGTGAGAAGCTGAAG-3' Reverse primer 5'-CACTGCCTTGCTCTTATTTTCACA-3'	This paper	N/A

TNFa Forward primer 5'-GCCCACGTCGTAGCAAACC-3' Reverse primer 5'-TAGCAAATCGGCTGACGGTG-3'	This paper	N/A
**Software and algorithms**		
3D Slicer v. 5.6.1	Fedorov, A. et al. [44]	https://download.slicer.org/
NDPView2 v .2.7.39	Hamamatsu	https://www.hamamatsu.com/jp/en/product/life-science-and-medical-systems/digital-slide-scanner/U12388-01.html
IMARIS X64 v. 9.3.0	Oxford Instruments	https://imaris.oxinst.com/imaris-viewer
Image J	Schindelin, J. et al. [45]	https://imagej.net/ij/
Leica Application Suite X (v. 3.7.6.25997)	Leica	https://www.leica-microsystems.com/products/microscope-software/p/leica-las-x-ls/downloads/
Open VMS	Scanco Medical	https://www.scanco.ch/clustering.html
IDEAS 6.2.189	Cytek Bioscience	https://cytekbio.com/pages/imagestream
MoFlo Astrios EQ 1 v 6.3.1	Beckman Coulter	https://www.beckman.de/flow-cytometry/cell-sorters/moflo-astrios-eq
Beckman Coulter Gallios software v 1.2	Beckman Coulter	https://www.beckman.de/flow-cytometry/software/kaluza-for-gallios
FlowJo v. 10.10	BD	https://www.bdbiosciences.com/en-us/products/software/flowjo-v10-software
Controller software v5.0	10X Genomics	https://www.10xgenomics.com/instruments/chromium-controller
QuantStudio 6 Real-Time PCR System v. 1.3	Thermo Fisher	https://www.thermofisher.com/de/de/home/global/forms/life-science/quantstudio-6-7-flex-software.html
CYTOF Software v7.1	Standard Biotools	https://www.standardbio.com/products/software/cytof-software-v7

Steinbock v. 0.16.0	Windhager, J. et al. [46]	https://github.com/BodenmillerGroup/steinbock/pkgs/container/steinbock
imcRtools v. 1.3.4	Windhager, J. et al. [46]	https://www.bioconductor.org/packages/release/bioc/html/imcRtools.html
homerTools v. 4.11.1	Heinz, S. et al.[40]	http://homer.ucsd.edu/homer/index.html
STAR v. 2.7.3a	Dobin, A. et al. [41]	https://github.com/alexdobin/STAR/releases/tag/2.7.3a
samtools v. 1.10	Danecek, P. et al. [42]	https://github.com/samtools/samtools/releases/tag/1.10
maegatk v. 0.2.0	Miller, TE. et al. [39]	https://github.com/caleblareau/maegatk
R v 4.3.2	Righelli, D. et al. [47]	https://cran.r-project.org/bin/windows/base/old/4.3.2/
R package Seurat 4.1.1	Hao, Y. et al. [1]	https://github.com/satijalab/seurat/releases/tag/v4.1.0
R package Seurat 5.2.1	Hao, Y. et al. [36]	https://github.com/satijalab/seurat/releases/tag/v5.2.1
R package Harmony 0.1.0	Korsunsky, I. et al. [3]	https://github.com/immunogenomics/harmony/releases/tag/0.1
R package singleR 1.10.0	Aran, D. et al. [4]	https://www.bioconductor.org/packages/release/bioc/html/SingleR.html
R package celldex 1.6.1	Aran et al. [4]	https://bioconductor.org/packages/release/data/experiment/html/celldex.html
R package UCell 2.0.1	Andreatta, M. and Carmona, SJ. [12]	https://github.com/carmonalab/UCell/releases/tag/v2.0
R package org.Mm.eg.db 3.15.0	Mark Carlson [13]	https://bioconductor.org/packages/release/data/annotation/html/org.Mm.eg.db.html
R package AnnotationDbi 1.66.0	Pagès, H. et al. [14]	https://bioconductor.org/packages/release/bioc/html/AnnotationDbi.html
R package SeuratDisk 0.0.0.9015	Paul Hoffman	https://github.com/mojaveazure/seurat-disk
R package diffcyt 1.22.0	Weber, LM. et	https://www.bioconduct

	al. [18]	or.org/packages/release/bioc/html/diffcyt.html
R package MiloR 1.10.0	Dann, E. et al. [19]	https://github.com/MarioniLab/miloR
R package hdWGCNA 0.3.00	Morabito, S. et al. [37]	https://github.com/smorabit/hdWGCNA/releases/tag/v0.3.00
R package SeuratWrapper 0.3.4	Rahul Satija Lab	https://github.com/satijalab/seurat-wrappers
R package scDblFinder 1.13.12	Germain, PL. et al. [29]	https://bioconductor.org/packages/release/bioc/html/scDblFinder.html
R package ClusterProfiler 4.4.4	Wu, T. et al. [26]	https://bioconductor.org/packages/release/bioc/html/clusterProfiler.html
R package TradeSeq 1.10.0	Van den Berge, K. et al. [25]	https://www.bioconductor.org/packages/release/bioc/html/tradeSeq.html
R package CellChat 1.5.0	Jin, S. et al. [27]	https://github.com/sqjin/CellChat/releases/tag/v1.5.0
R package igraph 2.1.4	Csárdi et al. [43]	https://github.com/igraph/rigraph/releases/tag/v2.1.4
Python MELD 1.0.0	Burkhardt, DB. et al [20]	https://github.com/KrishnaswamyLab/MELD/releases/tag/v1.0.0
Python graphtools 1.5.2	Smita Krishnaswamy Lab	https://github.com/KrishnaswamyLab/graphtools
Python Velocyto 0.17.17	La Manno, G. et al. [21]	https://github.com/velocyto-team/velocyto.py/releases/tag/0.17.17
Python scVelo 0.2.4	Bergen, V. et al. [22]	https://github.com/theislab/scvelo/releases/tag/v0.2.5
Python CellRank 2.0.2	Lange, M. et al. [23]	https://github.com/theislab/cellrank/releases/tag/v2.0.2
Python scvi-tools 1.1.1	Wolf, FA. et al. [32]	https://github.com/scverse/scvi-tools/releases/tag/1.1.1
Python Scanpy 1.9.8	Wolf, FA. et al. [32]	https://github.com/scverse/scvi-tools/releases/tag/1.1.1
Python mousipy 0.1.5	Stefan Peidli et al. [48]	https://github.com/stefanpeidli
Python scCODA 0.1.9	Buttner, M. et al. [15]	https://github.com/theislab/scCODA

References for Supplementary Material

1. Hao, Y. *et al.* Integrated analysis of multimodal single-cell data. *Cell* **184**, 3573-3587.e3529 (2021).

2. Hafemeister, C. & Satija, R. Normalization and variance stabilization of single-cell RNA-seq data using regularized negative binomial regression. *Genome Biol* **20**, 296 (2019).

3. Korsunsky, I. *et al.* Fast, sensitive and accurate integration of single-cell data with Harmony. *Nat Methods* **16**, 1289-1296 (2019).

4. Aran, D. *et al.* Reference-based analysis of lung single-cell sequencing reveals a transitional profibrotic macrophage. *Nat Immunol* **20**, 163-172 (2019).

5. Heng, T.S. & Painter, M.W. The Immunological Genome Project: networks of gene expression in immune cells. *Nat Immunol* **9**, 1091-1094 (2008).

6. Culemann, S. *et al.* Locally renewing resident synovial macrophages provide a protective barrier for the joint. *Nature* **572**, 670-675 (2019).

7. Alivernini, S. *et al.* Distinct synovial tissue macrophage subsets regulate inflammation and remission in rheumatoid arthritis. *Nat Med* **26**, 1295-1306 (2020).

8. Misharin, A.V. *et al.* Nonclassical Ly6C(-) monocytes drive the development of inflammatory arthritis in mice. *Cell Rep* **9**, 591-604 (2014).

9. Liu, Y. *et al.* Single-Cell Profiling Reveals Divergent, Globally Patterned Immune Responses in Murine Skin Inflammation. *iScience* **23**, 101582 (2020).

10. Open-source ImmGen: mononuclear phagocytes. *Nat Immunol* **17**, 741 (2016).

11. Gainullina, A. *et al.* Network analysis of large-scale ImmGen and Tabula Muris datasets highlights metabolic diversity of tissue mononuclear phagocytes. *Cell Rep* **42**, 112046 (2023).

12. Andreatta, M. & Carmona, S.J. UCell: Robust and scalable single-cell gene signature scoring. *Comput Struct Biotechnol J* **19**, 3796-3798 (2021).

13. Carlson, M. org.Mm.eg.db: Genome wide annotation for Mouse. *R package version*

3.8.2 (2019).

14. Pagès, H., Carlson, M., Falcon, S. & Li, N. AnnotationDbi: Manipulation of SQLite-based annotations in Bioconductor. *R package version 1.66.0* (2024).

15. Buttner, M., Ostner, J., Muller, C.L., Theis, F.J. & Schubert, B. scCODA is a Bayesian model for compositional single-cell data analysis. *Nat Commun* **12**, 6876 (2021).

16. Heumos, L. *et al.* Best practices for single-cell analysis across modalities. *Nat Rev Genet* **24**, 550-572 (2023).

17. Chen, Y., Lun, A.T. & Smyth, G.K. From reads to genes to pathways: differential expression analysis of RNA-Seq experiments using Rsubread and the edgeR quasi-likelihood pipeline. *F1000Res* **5**, 1438 (2016).

18. Weber, L.M., Nowicka, M., Soneson, C. & Robinson, M.D. diffcyt: Differential discovery in high-dimensional cytometry via high-resolution clustering. *Commun Biol* **2**, 183 (2019).

19. Dann, E., Henderson, N.C., Teichmann, S.A., Morgan, M.D. & Marioni, J.C. Differential abundance testing on single-cell data using k-nearest neighbor graphs. *Nat Biotechnol* **40**, 245-253 (2022).

20. Burkhardt, D.B. *et al.* Quantifying the effect of experimental perturbations at single-cell resolution. *Nat Biotechnol* **39**, 619-629 (2021).

21. La Manno, G. *et al.* RNA velocity of single cells. *Nature* **560**, 494-498 (2018).

22. Bergen, V., Lange, M., Peidli, S., Wolf, F.A. & Theis, F.J. Generalizing RNA velocity to transient cell states through dynamical modeling. *Nat Biotechnol* **38**, 1408-1414 (2020).

23. Lange, M. *et al.* CellRank for directed single-cell fate mapping. *Nat Methods* **19**, 159-170 (2022).

24. Reuter, B., Fackeldey, K. & Weber, M. Generalized Markov modeling of nonreversible molecular kinetics. *J Chem Phys* **150**, 174103 (2019).

25. Van den Berge, K. *et al.* Trajectory-based differential expression analysis for single-cell sequencing data. *Nat Commun* **11**, 1201 (2020).

26. Wu, T. *et al.* clusterProfiler 4.0: A universal enrichment tool for interpreting omics data. *Innovation (Camb)* **2**, 100141 (2021).

27. Jin, S. *et al.* Inference and analysis of cell-cell communication using CellChat. *Nat Commun* **12**, 1088 (2021).

28. Edalat, S.G. *et al.* Molecular maps of synovial cells in inflammatory arthritis using an optimized synovium dissociation protocol. *iScience*, 109707 (2024).

29. Germain, P.L., Lun, A., Garcia Meixide, C., Macnair, W. & Robinson, M.D. Doublet identification in single-cell sequencing data using scDblFinder. *F1000Res* **10**, 979 (2021).

30. Gayoso, A. *et al.* A Python library for probabilistic analysis of single-cell omics data. *Nat Biotechnol* **40**, 163-166 (2022).

31. Virshup, I. *et al.* The scverse project provides a computational ecosystem for single-cell omics data analysis. *Nat Biotechnol* **41**, 604-606 (2023).

32. Wolf, F.A., Angerer, P. & Theis, F.J. SCANPY: large-scale single-cell gene expression data analysis. *Genome Biol* **19**, 15 (2018).

33. Linderman, G.C. *et al.* Zero-preserving imputation of single-cell RNA-seq data. *Nat Commun* **13**, 192 (2022).

34. Reynolds, G. *et al.* Developmental cell programs are co-opted in inflammatory skin disease. *Science* **371** (2021).

35. Liu, J. *et al.* Combined Single Cell Transcriptome and Surface Epitope Profiling Identifies Potential Biomarkers of Psoriatic Arthritis and Facilitates Diagnosis via Machine Learning. *Front Immunol* **13**, 835760 (2022).

36. Hao, Y. *et al.* Dictionary learning for integrative, multimodal and scalable single-cell analysis. *Nat Biotechnol* **42**, 293-304 (2024).

37. Morabito, S., Reese, F., Rahimzadeh, N., Miyoshi, E. & Swarup, V. hdWGCNA identifies co-expression networks in high-dimensional transcriptomics data. *Cell Rep Methods* **3**, 100498 (2023).

38. Good, J.D., Safina, K.R., Miller, T.E. & van Galen, P. Protocol for mitochondrial variant enrichment from single-cell RNA sequencing using MAESTER. *STAR Protoc* **6**, 103564 (2025).

39. Miller, T.E. *et al.* Mitochondrial variant enrichment from high-throughput single-cell RNA sequencing resolves clonal populations. *Nat Biotechnol* **40**, 1030-1034 (2022).

40. Heinz, S. *et al.* Simple combinations of lineage-determining transcription factors prime cis-regulatory elements required for macrophage and B cell identities. *Mol Cell* **38**, 576-589 (2010).

41. Dobin, A. *et al.* STAR: ultrafast universal RNA-seq aligner. *Bioinformatics* **29**, 15-21 (2013).

42. Danecek, P. *et al.* Twelve years of SAMtools and BCFtools. *Gigascience* **10** (2021).

43. Csárdi, G. & Nepusz, T. The igraph software package for complex network research. 2006; 2006.

44. Fedorov, A. *et al.* 3D Slicer as an image computing platform for the Quantitative Imaging Network. *Magn Reson Imaging* **30**, 1323-1341 (2012).

45. Schindelin, J. *et al.* Fiji: an open-source platform for biological-image analysis. *Nat Methods* **9**, 676-682 (2012).

46. Windhager, J. *et al.* An end-to-end workflow for multiplexed image processing and analysis. *Nat Protoc* **18**, 3565-3613 (2023).

47. Righelli, D. *et al.* SpatialExperiment: infrastructure for spatially-resolved transcriptomics data in R using Bioconductor. *Bioinformatics* **38**, 3128-3131 (2022).

48. Peidli, S., ianfd & pakiessling. stefanpeidli/mousipy: v0.1.7. v0.1.7 ed: Zenodo; 2025.
