# Supplementary material for: Skin-derived myeloid precursors and joint-resident fibroblasts spread psoriatic disease from skin to joints
Source: Nat Immunol. 2026 Jan 2;27(1):35–47. doi: 10.1038/s41590-025-02351-z (PMC12764428; doi:10.1038/s41590-025-02351-z)
Supplement: Supplementary file 2 — Reporting Summary [file 41590_2025_2351_MOESM2_ESM.pdf]

nature portfolio

Corresponding author(s): Andreas Ramming

Last updated by author(s): 2025/07/18

Reporting Summary

Nature Portfolio wishes to improve the reproducibility of the work that we publish. This form provides structure for consistency and transparency in reporting. For further information on Nature Portfolio policies, see our Editorial Policies and the Editorial Policy Checklist.

Statistics

For all statistical analyses, confirm that the following items are present in the figure legend, table legend, main text, or Methods section.

n/a	Confirmed	
☐	☒	The exact sample size (*n*) for each experimental group/condition, given as a discrete number and unit of measurement
☐	☒	A statement on whether measurements were taken from distinct samples or whether the same sample was measured repeatedly
☐	☒	The statistical test(s) used AND whether they are one- or two-sided<br>*Only common tests should be described solely by name; describe more complex techniques in the Methods section.*
☒	☐	A description of all covariates tested
☐	☒	A description of any assumptions or corrections, such as tests of normality and adjustment for multiple comparisons
☐	☒	A full description of the statistical parameters including central tendency (e.g. means) or other basic estimates (e.g. regression coefficient) AND variation (e.g. standard deviation) or associated estimates of uncertainty (e.g. confidence intervals)
☐	☒	For null hypothesis testing, the test statistic (e.g. *F*, *t*, *r*) with confidence intervals, effect sizes, degrees of freedom and *P* value noted<br>*Give P values as exact values whenever suitable.*
☒	☐	For Bayesian analysis, information on the choice of priors and Markov chain Monte Carlo settings
☒	☐	For hierarchical and complex designs, identification of the appropriate level for tests and full reporting of outcomes
☐	☒	Estimates of effect sizes (e.g. Cohen's *d*, Pearson's *r*), indicating how they were calculated

Our web collection on statistics for biologists contains articles on many of the points above.

Software and code

Policy information about availability of computer code

Data collection

MRI volumetric data were analyzed with 3D Slicer v. 5.6.1; epidermal thickness was measured with NDPView2 v .2.7.39; light sheet microscopy data were processed with IMARIS X64 (Oxford Instruments) software (v. 9.3.0), Leica Application Suite X (v. 3.7.6.25997) and Fiji (v. 1.52); ImageStream Data Exploration and Analysis Software IDEAS 6.2.189 Cytek was used for Imaging cytometry; for bone volume and osteoproliferation Open VMS (Scanco Medical) was used; for cell sorting on BD Astrios Summit v 6.3.1 was used; for acquisition of cytometry data, Beckman Coulter Gallios software v 1.2 was used; for flow cytometry data analysis FlowJo v. 10.10, BD Biosciences was used; for generation of 10x libraries on Chromium Controller software v5.0 was used; for generation of RT-PCR data QuantStudio 6 Real-Time PCR System v. 1.3 was used; for the acquisition of imaging mass cytometry data Standard Biotools CYTOF Software v7.1 was used. MCD files were processed using Steinbock v. 0.16.0, imcRtools v. 1.3.4 and R v 4.3.2.

Single cell RNA sequenicng data analysis was done in Python 3.8, 3.9 or 3.10 and R 4.0 or 4.2, depending on the package requirements. The following packages/pipelines were used: Seurat 4.1.1 or 5.2.1, Harmony 0.1.0 or 1.2.3, singleR 1.10.0, celldex 1.6.1, UCell 2.0.1, org.Mm.eg.db 3.15.0, AnnotationDbi 1.66.0, SeuratDisk 0.0.0.9015, diffcyt 1.22.0, MiloR 1.10.0, MELD 1.0.0, graphtools 1.5.2, Velocyto 0.17.17, scVelo 0.2.4, CellRank 2.0.2, TradeSeq 1.10.0, ClusterProfiler 4.4.4, CellChat 1.5.0, scDblFinder 1.13.12, scvi-tools 1.1.1, Scanpy 1.9.8, SeuratWrapper 0.3.4, mousipy 0.1.5, hdWGCNA 0.3.00, scCODA 0.1.9, homerTools 4.11.1, samtools 1.10, STAR 2.7.3a, maegatk 0.2.0, igraph 2.1.4

Data analysis

no custom software was developed; the use of any software is mentioned in the material and methods section including version numbers.

For manuscripts utilizing custom algorithms or software that are central to the research but not yet described in published literature, software must be made available to editors and reviewers. We strongly encourage code deposition in a community repository (e.g. GitHub). See the Nature Portfolio guidelines for submitting code & software for further information.

Data

Policy information about availability of data

All manuscripts must include a data availability statement. This statement should provide the following information, where applicable:

- Accession codes, unique identifiers, or web links for publicly available datasets
- A description of any restrictions on data availability
- For clinical datasets or third party data, please ensure that the statement adheres to our policy

single cell RNA sequence data that support the findings of this study will be deposited in Gene Expression Omnibus (GEO) when the manuscript is accepted for publication. Individual-level mitoseq data are available under restricted access because of patient privacy and ethical considerations. Qualified researchers may request access by contacting the corresponding author. Requests will be reviewed to ensure compliance with institutional and regulatory policies. Responses to data access requests can be expected within 4 weeks.

Human research participants

Policy information about studies involving human research participants and Sex and Gender in Research.

Reporting on sex and gender	Gender was not considered in this study. Sex was not considered as an inclusion criterion.
Population characteristics	Human research was conducted in accordance with approved protocols by the institutional review boards of the Friedrich-Alexander-University (FAU) of Erlangen-Nürnberg, the Fondazione Policlinico Gemelli IRCCS and the IRB of Hospital Clinic de Barcelona. Ultrasound guided-minimally invasive synovial tissue biopsies were collected from early PsA (N = 5) and patients with psoriasis (N = 6) at the SYNGem Biopsy Unit of the Fondazione Policlinico Universitario A. Gemelli RCCS. Additional synovial tissue from healthy donors (HC; N = 3) was obtained in Barcelona. Blood samples were collected from individuals with PsA (N = 10) in Erlangen. Patient information is provided in Suppl. Tab. 1. All patients included fulfilled the 2006 CASPAR classification criteria for PsA 42. Additional human datasets were obtained from online databases listed in the data availability section and referenced in the text and figure legends.
Recruitment	Human samples were obtained from research volunteers of the University Hospital Erlangen and IRB of Hospital Clínic de Barcelona. Written informed consent was obtained from all subjects. There was no self-selection bias involved.
Ethics oversight	Human subjects research was performed according to the Institutional Review Boards at the University Hospital Erlangen, Fondazione Policlinico Gemelli IRCCS and of the Hospital Clinic de Barcelona (Barcelona Research Ethics Committee) via approved protocols.

Note that full information on the approval of the study protocol must also be provided in the manuscript.

Field-specific reporting

Please select the one below that is the best fit for your research. If you are not sure, read the appropriate sections before making your selection.

☒ Life sciences ☐ Behavioural & social sciences ☐ Ecological, evolutionary & environmental sciences

For a reference copy of the document with all sections, see nature.com/documents/nr-reporting-summary-flat.pdf

Life sciences study design

All studies must disclose on these points even when the disclosure is negative.

Sample size	The sample size was determined from preliminary experiments.
Data exclusions	No samples were excluded.
Replication	The key findings in the study were reliably reproduced in independent cohorts. Key findings were reproduced by the use of different experimental models or public online data sets. Replicates for each type of experiment are indicated.
Randomization	Animals were randomized by sex. All animals were 8-10 weeks old at the start of the experiment.
Blinding	The investigators were blinded to the evaluation of MRI imaging and histological analysis and evaluation. The clinical evaluation of the mice during arthritis was not blinded to ensure full animal welfare.

Reporting for specific materials, systems and methods

We require information from authors about some types of materials, experimental systems and methods used in many studies. Here, indicate whether each material, system or method listed is relevant to your study. If you are not sure if a list item applies to your research, read the appropriate section before selecting a response.

Materials & experimental systems

n/a	Involved in the study	
☐	☒	Antibodies
☒	☐	Eukaryotic cell lines
☒	☐	Palaeontology and archaeology
☐	☒	Animals and other organisms
☐	☒	Clinical data
☒	☐	Dual use research of concern

Methods

n/a	Involved in the study	
☒	☐	ChIP-seq
☐	☒	Flow cytometry
☒	☐	MRI-based neuroimaging

Antibodies

Antibodies used

Application / Antigen / Label / Supplier / Catalogue / Clone / Dilution
FC-Mo hu / CD3 / BV 510 / Biolegend / 300448 / UCHT1 / 100x
FC-Mo hu / CD19 / BV 510 / Biolegend / 302242 / HIB19 / 200x
FC-Mo hu / CD56 / BV 510 / Biolegend / 318340 / HCD56 / 200x
FC-Mo hu / CD11c / BV 421 / Biolegend / 301627 / 3,9 / 100x
FC-Mo hu / CD2 / APC / Biolegend / 300214 / RPA-2.10 / 1000x
FC-Mo hu / HLA-DR / APC/Cy7 / Biolegend / 307618 / L243 / 1000x
FC-Mo hu / CD14 / PE/Cy7 / Biolegend / 301814 / M5E2 / 100x
FC-Mo hu / CD45 / PerCP/Cy5.5 / Biolegend / 304028 / HI30 / 100x
FC-Mo hu / CD200R / PE / Biolegend / 329306 / OX-108 / 100x
FC-Mo hu / CD192 / PE / Biolegend / 357206 / K036C2 / 500x
FC-Mo hu / CD1C / PE / Biolegend / 331506 / L161 / 1000x
FC-Mo hu / CD123 / PE / Biolegend / 306006 / 6H6 / 1000x
FC-Mo hu / CD209 / PE / Biolegend / 330106 / 9E9A8 / 200x
FC-Mo hu / IgG1, κ Isotype Ctrl / PE / Biolegend / 400114 / MOPC-21 / 100x
FC-Mo hu / IgG2a, κ Isotype Ctrl / PE / Biolegend / 400214 / MOPC-173 / 200x
FC-Mo ms / CD45 / FITC / Biolegend / 103108 / 30-F11 / 1000x
FC-Mo ms / Ly6G / PE / Biolegend / 127608 / 1A8 / 1000x
FC-Mo ms / CD11b / BV 421 / Biolegend / 101236 / M1/70 / 1000x
FC-Mo ms / CD2 / APC / Biolegend / 100112 / RM2-5 / 1000x
FC-Mo ms / I-A/I-E / BV510 / Biolegend / 107636 / M5/114.15.2 / 500x
FC-Mo ms / CD3ε / PE/Cy7 / Biolegend / 100320 / 145-2C11 / 100x
FC-Mo ms / CD45R/B220 / PE/Cy7 / Biolegend / 103222 / RA3-6B2 / 200x
FC-Mo ms / CD11c / PerCP/Cy5.5 / Biolegend / 117328 / N418 / 500x
FC-Fib ms / CD140a / BV 421 / Biolegend / 135923 / APA5 / 500x
FC-Fib ms / CD45 / PE/Cy7 / Biolegend / 103114 / 30-F11 / 1000x
FC-Fib ms / CD90.2 / BV 510 / Biolegend / 105335 / 30-H12 / 500x
FC-Fib ms / Podoplanin / PerCP/Cy5.5 / Biolegend / 127422 / 8.1.1 / 200x
FC-Fib ms / CD31 / PE/Cy7 / Biolegend / 102418 / 390 / 500x
FC-Fib ms / CD49f / FITC / Biolegend / 313606 / GoH3 / 200x
FC-Fib ms / CD200 / APC / Biolegend / 123810 / OX-90 / 100x
FC-KAEDE / CD45 / APC / Biolegend / 103112 / 30-F11 / 1000x
ImageStream KAEDE / CD3ε / PE/Cy7 / Biolegend / 100320 / 145-2C11 / 100x
ImageStream KAEDE / CD45R/B220 / PE/Cy7 / Biolegend / 103222 / RA3-6B2 / 200x
ImageStream KAEDE / CD11b / APC/Cy7 / Biolegend / 101226 / M1/70 / 1000x
ImageStream KAEDE / CD45 / APC / Biolegend / 103112 / 30-F11 / 1000x
FCS-Mo Ly-6G BV 421 Biolegemd 127628 1A8 100x
FCS-Mo / CD45 / APC / Biolegend / 103112 / 30-F11 / 1000x
scRNAseq / Hashtag 1 / ACCCACCAGTAAGAC / Biolegend / 155831 / M1/42; 30-F11; / 100x
scRNAseq / Hashtag 2 / GGTCGAGAGCATTCA / Biolegend / 155833 / M1/42; 30-F11; / 200x
scRNAseq / Allophycocyanin / TTAACCGTCTCCCTT / Biolegend / 408009 / APC003 / 200x
scRNAseq / Hashtag 3 / CTTGCCGCATGTCAT / Biolegend / 155835 / M1/42; 30-F11; / 200x
scRNAseq / Hashtag 4 / AAAGCATTCTTCACG / Biolegend / 155837 / M1/42; 30-F11; / 200x
scRNAseq / Hashtag 5 / CTTTGTCTTTGTGAG / Biolegend / 155839 / M1/42; 30-F11; / 200x
IMC / Mast Cell Tryptase / 89Y / Biolegend / 369402 / AA1 / 500x
IMC / ACTA2 / 113In / Sigma Aldrich / A5228-100UL / 1A4 / 500x
IMC / Pan-Cadherin / 115In / Thermo / 71-7100 / Polyclonal rabbit / 200x
IMC / B2M / 115ln / abcam / ab237032 / EPR21752-214 / 50x
IMC / CCR2 / 141Pr / Thermo / PA5-23037 / Polyclonal rabbit / 100x
IMC / CD20 / 142Nd / Thermo / 14-0202-82 / L26 / 100x
IMC / CD16 / 143Nd / abcam / ab198507 / EPR16784 / 100x
IMC / CD14 / 144Nd / Standard Biotools / 3144025D / EPR3653 / 400x
IMC / CD2 / 145Nd / abcam / ab248400 / EPR6451 / 100x
IMC / Cd8a / 146Nd / Novus Biologicals / NBP2-34588 / C8/144B / 100x
IMC / CD68 / 147Sm / Thermo / 14-0688-82 / KP1 / 200x
IMC / CD161 / 148Nd / abcam / ab302565 / EPR26340-6 / 100x

IMC / CD11b / 149Sm / abcam / ab209970 / EPR1344 / 100x
IMC / MERTK / 150Nd / abcam / ab271851 / Y323 / 100x
IMC / CD31 / 151Eu / abcam / ab207090 / EPR3094 / 200x
IMC / CD45 / 152Sm / Cell Signaling / 47937 / D9M8I / 100x
IMC / CD206 / 153Eu / Abnova / H00004360-M02 / 5C11 / 200x
IMC / CD11c / 154Sm / abcam / ab216655 / Polyclonal rabbit / 50x
IMC / FOXP3 / 155Gd / Thermo / 14-4776-82 / PCH101 / 100x
IMC / CD4 / 156Gd / abcam / ab181724 / EPR6855 / 100x
IMC / S100A12 / 158Gd / abcam / ab273051 / EPR23677-111 / 100x
IMC / hDkk3 / 159Tb / R&D systems / AF1118 / Polyclonal goat / 100x
IMC / LYVE1 / 160Gd / abcam / ab314242 / RM1067 / 500x
IMC / CD200R / 162Dy / abcam / ab232941 / EPR21232 / 100x
IMC / CD15 / 163Dy / Biolegend / 323002 / W6D3 / 100x
IMC / Podoplanin / 164Dy / Biolegend / 916606 / D2-40 / 200x
IMC / CD123 / 165Ho / abcam / ab269871 / EPR23188-72 / 100x
IMC / CD163 / 166Er / Thermo / MA1-82342 / EDHu-1 / 200x
IMC / CD90 / 167Er / R&D systems / AF2067 / Polyclonal sheep / 200x
IMC / CD127 / 168Er / Standard Biotools / 3168026D / EPR2955(2) / 200x
IMC / HLA-DR / 169Tm / Biolegend / 327002 / LN3 / 100x
IMC / CD3ε / 170Er / Cell Signaling / 24581SF / D7A6E / 100x
IMC / CD200 / 171Yb / Cell Signaling / 24510SF / E5I9V / 100x
IMC / IL23R / 172Yb / Thermo / PA5-102441 / Polyclonal rabbit / 100x
IMC / FAP / 173Yb / R&D systems / AF3715 / Polyclonal sheep / 50x
IMC / CD55 / 174Yb / abcam / ab133684 / EPR6689 / 100x
IMC / CD1c / 175Lu / OriGene / CF505411 / OTI2F4 / 100x
IMC / Histone H3 / 176Yb / Standard Biotools / 3176023D / D1H2 / 800x
IMC / ICSK1 / 195Pt / Standard Biotools / TIS-00001 / not specified / 50x
IMC / ICSK2 / 196Pt / Standard Biotools / TIS-00001 / not specified / 50x
IMC / ICSK3 / 198Pt / Standard Biotools / TIS-00001 / not specified / 50x
IN VIVO / CD200 / / BioXCell / BE0299 / OX-90 /
IN VITRO / CD200 / / Biolegend / 123802 / OX-90 /
IN VITRO / Rat IgG2a, κ Isotype Ctrl / / Biolegend / 400573 / RTK2758 /
FC-sorting ms / CD45 / BV 421 / Biolegend / 103134 / 30-F11 / 500x
FC-sorting ms / CD11b / FITC / Biolegend / 101206 / M1/70 / 200x
FC-sorting ms / I-A/I-E / BV 510 / Biolegend / 107636 / M5/114.15.2 / 100x
FC-sorting ms / CD301 / APC / Biolegend / 145708 / LOM-14 / 100x
FC-sorting ms / CD2 / PE / Biolegend / 100108 / RM2-5 / 500x
FC-sorting ms / CD11c / APC/Cy7 / Biolegend / 117324 / N418 / 200x
FC-sorting ms / CD3ε / APC/Cy7 / Biolegend / 100222 / 17A2 / 100x
FC-sorting ms / CD45R/B220 / APC/Cy7 / Biolegend / 103224 / RA3-6B2 / 200x
FC-sorting ms / CD197 / PE/Cy7 / Biolegend / 120124 / 4B12 / 100x
FC-sorting ms / Ly6G / PerCP/Cy5.5 / Biolegend / 127616 / 1A8 / 500x
Cytek-Mo&Fib ms / Podoplanin / BV 421 / Biolegend / 127423 / 8.1.1 / 250x
Cytek-Mo&Fib ms / Ly6G / BV 480 / BD Bioscience / 746448 / 1A8 / 500x
Cytek-Mo&Fib ms / CD140a / BV 510 / Biolegend / 135923 / APA5 / 500x
Cytek-Mo&Fib ms / CD90.2 / BV 570 / Biolegend / 105329 / 30-H12 / 1000x
Cytek-Mo&Fib ms / CD2 / BV 605 / BD Bioscience / 740338 / RM2-5 / 1000x
Cytek-Mo&Fib ms / CD31 / BV 650 / BD Bioscience / 740483 / 390 / 200x
Cytek-Mo&Fib ms / I-A/I-E / BV 711 / Biolegend / 107643 / M5/114.15.2 / 1000x
Cytek-Mo&Fib ms / CD45R/B220 / BV 785 / Biolegend / 103246 / RA3-6B2 / 400x
Cytek-Mo&Fib ms / CD45 / FITC / Biolegend / 103108 / 30-F11 / 1000x
Cytek-Mo&Fib ms / CD200 / PE / Biolegend / 123808 / OX-90 / 200x
Cytek-Mo&Fib ms / CD49f / PerCP/Cy5.5 / Biolegend / 313618 / GoH3 / 200x
Cytek-Mo&Fib ms / CD11c / PE/Cy7 / Biolegend / 117318 / N418 / 200x
Cytek-Mo&Fib ms / CD11b / APC / Biolegend / 101212 / M1/70 / 1000x
Cytek-Mo&Fib ms / CD3ε / APC/Fire810 / Biolegend / 100268 / 17A2 / 200x
FC-sorting hu / HLA-DR / APC / Biolegend / 307610 / L243 / 200x
FC-sorting hu / CD3ε / BV 510 / Biolegend / 300448 / UCHT1 / 200x
FC-sorting hu / CD8a / FITC / Biolegend / 344704 / SK1 / 1000x
FC-sorting hu / CD4 / PerCP/Cy5.5 / Biolegend / 357414 / A161A1 / 200x
FC-sorting hu / CD192 / BV 421 / Biolegend / 357210 / K036C2 / 500x
FC-sorting hu / CD2 / PE / Biolegend / 300208 / RPA-2.10 / 200x
FC-sorting hu / CD1c / PE/Cy7 / Biolegend / 331516 / L161 / 500x
FC-sorting hu / CD123 / PE/Cy7 / Biolegend / 983702 / 6H6 / 500x
FC-sorting hu / CD19 / Spark PLUS UV395 / Biolegend / 302298 / HIB19 / 200x
FC-Coculture hu / CD3ε / BV 510 / Biolegend / 300448 / UCHT1 / 200x
FC-Coculture hu / CD4 / PerCP/Cy5.5 / Biolegend / 357414 / A161A1 / 200x
FC-Coculture hu / CD8a / FITC / Biolegend / 344704 / SK1 / 1000x
FC-Coculture hu / IL-17A / PE/Cy7 / Biolegend / 512315 / BL168 / 500x
Invivo-CD200R1 ms / CD200R / / Biolegend / 123918 / OX-110 /

Validation

All antibodies are commercially available. Validation was provided by the supplier:
Application / Antigen / Supplier / Catalogue / Reference
FC-Mo hu / CD3 / Biolegend / 300448 / https://www.biolegend.com/de-de/products/brilliant-violet-510-anti-human-cd3-antibody-9792
FC-Mo hu / CD19 / Biolegend / 302242 / https://www.biolegend.com/de-de/products/brilliant-violet-510-anti-human-cd19-antibody-8004
FC-Mo hu / CD56 / Biolegend / 318340 / https://www.biolegend.com/de-de/products/brilliant-violet-510-anti-human-cd56-ncam-antibody-8011
FC-Mo hu / CD11c / Biolegend / 301627 / https://www.biolegend.com/de-de/products/brilliant-violet-421-anti-human-cd11c-antibody-7309
FC-Mo hu / CD2 / Biolegend / 300214 / https://www.biolegend.com/de-de/products/apc-anti-human-cd2-antibody-7219
FC-Mo hu / HLA-DR / Biolegend / 307618 / https://www.biolegend.com/de-de/products/apc-cyanine7-anti-human-hla-dr-antibody-2863
FC-Mo hu / CD14 / Biolegend / 301814 / https://www.biolegend.com/de-de/products/pe-cyanine7-anti-human-cd14-antibody-2729
FC-Mo hu / CD45 / Biolegend / 304028 / https://www.biolegend.com/de-de/products/percp-cyanine5-5-anti-human-cd45-antibody-4240
FC-Mo hu / CD200R / Biolegend / 329306 / https://www.biolegend.com/de-de/products/pe-anti-human-cd200-receptor-antibody-4612
FC-Mo hu / CD192 / Biolegend / 357206 / https://www.biolegend.com/de-de/products/pe-anti-human-cd192-ccr2-antibody-8529
FC-Mo hu / CD1C / Biolegend / 331506 / https://www.biolegend.com/de-de/products/pe-anti-human-cd1c-antibody-4847
FC-Mo hu / CD123 / Biolegend / 306006 / https://www.biolegend.com/de-de/products/pe-anti-human-cd123-antibody-577
FC-Mo hu / CD209 / Biolegend / 330106 / https://www.biolegend.com/de-de/products/pe-anti-human-cd209-dc-sign-antibody-4885
FC-Mo hu / IgG1, κ Isotype Ctrl / Biolegend / 400114 / https://www.biolegend.com/de-de/products/pe-mouse-igg1-kappa-isotype-ctrl-fc-3035
FC-Mo hu / IgG2a, κ Isotype Ctrl / Biolegend / 400214 / https://www.biolegend.com/de-de/products/pe-mouse-igg2a-kappa-isotype-ctrl-fc-3043
FC-Mo ms / CD45 / Biolegend / 103108 / https://www.biolegend.com/de-de/products/fitc-anti-mouse-cd45-antibody-99
FC-Mo ms / Ly6G / Biolegend / 127608 / https://www.biolegend.com/de-de/products/pe-anti-mouse-ly-6g-antibody-4777
FC-Mo ms / CD11b / Biolegend / 101236 / https://www.biolegend.com/de-de/products/brilliant-violet-421-anti-mouse-human-cd11b-antibody-7163
FC-Mo ms / CD2 / Biolegend / 100112 / https://www.biolegend.com/de-de/products/apc-anti-mouse-cd2-antibody-9287
FC-Mo ms / I-A/I-E / Biolegend / 107636 / https://www.biolegend.com/de-de/products/brilliant-violet-510-anti-mouse-i-a-i-e-antibody-7997
FC-Mo ms / CD3ε / Biolegend / 100320 / https://www.biolegend.com/de-de/products/pe-cyanine7-anti-mouse-cd3epsilon-antibody-1899
FC-Mo ms / CD45R/B220 / Biolegend / 103222 / https://www.biolegend.com/de-de/products/pe-cyanine7-anti-mouse-human-cd45r-b220-antibody-1930
FC-Mo ms / CD11c / Biolegend / 117328 / https://www.biolegend.com/de-de/products/percp-cyanine5-5-anti-mouse-cd11c-antibody-4258
FC-Fib ms / CD140a / Biolegend / 135923 / https://www.biolegend.com/de-de/products/brilliant-violet-421-anti-mouse-cd140a-antibody-17921
FC-Fib ms / CD45 / Biolegend / 103114 / https://www.biolegend.com/de-de/products/pe-cyanine7-anti-mouse-cd45-antibody-1903
FC-Fib ms / CD90.2 / Biolegend / 105335 / https://www.biolegend.com/de-de/products/brilliant-violet-510-anti-mouse-cd90-2-thy1-2-antibody-12531
FC-Fib ms / Podoplanin / Biolegend / 127422 / https://www.biolegend.com/de-de/products/percp-cyanine5-5-anti-mouse-podoplanin-antibody-18189
FC-Fib ms / CD31 / Biolegend / 102418 / https://www.biolegend.com/de-de/products/pe-cyanine7-anti-mouse-cd31-antibody-3942
FC-Fib ms / CD49f / Biolegend / 313606 / https://www.biolegend.com/de-de/products/fitc-anti-human-mouse-cd49f-antibody-2606
FC-Fib ms / CD200 / Biolegend / 123810 / https://www.biolegend.com/de-de/products/apc-anti-mouse-cd200-ox2-antibody-7338
FC-KAEDE / CD45 / Biolegend / 103112 / https://www.biolegend.com/de-de/products/apc-anti-mouse-cd45-antibody-97
ImageStream KAEDE / CD3ε / Biolegend / 100320 / https://www.biolegend.com/de-de/products/pe-cyanine7-anti-mouse-cd3epsilon-antibody-1899
ImageStream KAEDE / CD45R/B220 / Biolegend / 103222 / https://www.biolegend.com/de-de/products/pe-cyanine7-anti-mouse-human-cd45r-b220-antibody-1930
ImageStream KAEDE / CD11b / Biolegend / 101226 / https://www.biolegend.com/de-de/products/apc-cyanine7-anti-mouse-human-cd11b-antibody-3930
ImageStream KAEDE / CD45 / Biolegend / 103112 / https://www.biolegend.com/de-de/products/apc-anti-mouse-cd45-antibody-97
FCS-Mo / Ly-6G / Biolegemd / 127628 / https://www.biolegend.com/de-de/products/brilliant-violet-421-anti-mouse-ly-6g-antibody-7161
FCS-Mo / CD45 / Biolegend / 103112 / https://www.biolegend.com/de-de/products/apc-anti-mouse-cd45-antibody-97
scRNAseq / Hashtag 1 / Biolegend / 155831 / https://www.biolegend.com/de-de/products/totalseq-b0301-anti-mouse-hashtag-1-antibody-17771
scRNAseq / Hashtag 2 / Biolegend / 155833 / https://www.biolegend.com/de-de/products/totalseq-b0302-anti-mouse-hashtag-2-antibody-17772
scRNAseq / Allophycocyanin / Biolegend / 408009 / https://www.biolegend.com/de-de/products/totalseq-b0987-anti-allophycocyanin-apc-antibody-21174
scRNAseq / Hashtag 3 / Biolegend / 155835 / https://www.biolegend.com/de-de/products/totalseq-b0303-anti-mouse-hashtag-3-antibody-17773
scRNAseq / Hashtag 4 / Biolegend / 155837 / https://www.biolegend.com/de-de/products/totalseq-b0304-anti-mouse-hashtag-4-antibody-17774
scRNAseq / Hashtag 5 / Biolegend / 155839 / https://www.biolegend.com/de-de/products/totalseq-b0305-anti-mouse-hashtag-5-antibody-17775
IMC / Mast Cell Tryptase / Biolegend / 369402 / https://www.biolegend.com/de-de/products/purified-anti-human-mast-cell-

tryptase-antibody-12681
IMC / ACTA2 / Sigma Aldrich / A5228-100UL / https://www.sigmaaldrich.com/DE/de/product/sigma/a5228
IMC / Pan-Cadherin / Thermo / 71-7100 / https://www.thermofisher.com/antibody/product/Pan-cadherin-Antibody-Polyclonal/71-7100
IMC / B2M / abcam / ab237032 / https://www.abcam.com/en-de/products/primary-antibodies/beta-2-microglobulin-antibody-epr21752-214-bsa-and-azide-free-ab237032#tab=datasheet
IMC / CCR2 / Thermo / PA5-23037 / https://www.thermofisher.com/antibody/product/CCR2-Antibody-Polyclonal/PA5-23037
IMC / CD20 / Thermo / 14-0202-82 / https://www.thermofisher.com/antibody/product/CD20-Antibody-clone-L26-Monoclonal/14-0202-82
IMC / CD16 / abcam / ab198507 / https://www.abcam.com/en-de/products/primary-antibodies/cd16-antibody-epr16784-c-terminal-ab198507#tab=datasheet
IMC / CD14 / Standard Biotools / 3144025D / https://store.standardbio.com/product_detail/guest-catalog/3144025d
IMC / CD2 / abcam / ab248400 / https://www.abcam.com/en-de/products/primary-antibodies/cd2-antibody-epr6451-bsa-and-azide-free-ab248400#
IMC / Cd8a / Novus Biologicals / NBP2-34588 / https://www.novusbio.com/products/cd8-antibody-c8-144b_nbp2-34588
IMC / CD68 / Thermo / 14-0688-82 / https://www.thermofisher.com/antibody/product/CD68-Antibody-clone-KP1-Monoclonal/14-0688-82
IMC / CD161 / abcam / ab302565 / https://www.abcam.com/en-de/products/primary-antibodies/cd161-antibody-epr26340-6-bsa-and-azide-free-ab302565#
IMC / CD11b / abcam / ab209970 / https://www.abcam.com/en-de/products/primary-antibodies/cd11b-antibody-epr1344-bsa-and-azide-free-ab209970
IMC / MERTK / abcam / ab271851 / https://www.abcam.com/en-de/products/primary-antibodies/mertk-antibody-y323-bsa-and-azide-free-ab271851
IMC / CD31 / abcam / ab207090 / https://www.abcam.com/en-de/products/primary-antibodies/cd31-antibody-epr3094-bsa-and-azide-free-ab207090
IMC / CD45 / Cell Signaling / 47937 / https://www.cellsignal.com/products/primary-antibodies/cd45-intracellular-domain-d9m8i-xp-rabbit-mab-bsa-and-azide-free/47937
IMC / CD206 / Abnova / H00004360-M02 / https://www.abnova.com/en-global/product/detail/H00004360-M02
IMC / CD11c / abcam / ab216655 / https://www.abcam.com/en-de/products/primary-antibodies/cd11c-antibody-ep1347y-bsa-and-azide-free-ab216655
IMC / FOXP3 / Thermo / 14-4776-82 / https://www.thermofisher.com/antibody/product/FOXP3-Antibody-clone-PCH101-Monoclonal/14-4776-82
IMC / CD4 / abcam / ab181724 / https://www.abcam.com/en-de/products/primary-antibodies/cd4-antibody-epr6855-bsa-and-azide-free-ab181724#
IMC / S100A12 / abcam / ab273051 / https://www.abcam.com/en-de/products/primary-antibodies/s100a12-cgrp-antibody-epr23677-111-bsa-and-azide-free-ab273051#tab=datasheet
IMC / hDkk3 / R&D systems / AF1118 / https://www.rndsystems.com/products/human-dkk-3-antibody_af1118
IMC / LYVE1 / abcam / ab314242 / https://www.abcam.com/en-de/products/primary-antibodies/lyve1-antibody-rm1067-bsa-and-azide-free-ab314242#
IMC / CD200R / abcam / ab232941 / https://www.abcam.com/en-de/products/primary-antibodies/cd200r-antibody-epr21232-ab232941
IMC / CD15 / Biolegend / 323002 / https://www.biolegend.com/de-de/products/purified-anti-human-cd15-ssea-1-antibody-3699
IMC / Podoplanin / Biolegend / 916606 / https://www.biolegend.com/de-de/products/purified-anti-podoplanin-lymphatic-endothelial-marker-antibody-11959
IMC / CD123 / abcam / ab269871 / https://www.abcam.com/en-de/products/primary-antibodies/il3ra-cd123-antibody-epr23188-72-bsa-and-azide-free-ab269871#
IMC / CD163 / Thermo / MA1-82342 / https://www.thermofisher.com/antibody/product/CD163-Antibody-clone-EDHu-1-Monoclonal/MA1-82342
IMC / CD90 / R&D systems / AF2067 / https://www.rndsystems.com/products/human-porcine-canine-cd90-thy1-antibody_af2067
IMC / CD127 / Standard Biotools / 3168026D / https://store.standardbio.com/product_detail/guest-catalog/3168026d
IMC / HLA-DR / Biolegend / 327002 / https://www.biolegend.com/de-de/products/purified-anti-human-hla-dr-antibody-4163
IMC / CD3ε / Cell Signaling / 24581SF / https://www.cellsignal.com/products/primary-antibodies/cd3e-d7a6e-xp-rabbit-mab-bsa-and-azide-free/24581
IMC / CD200 / Cell Signaling / 24510SF / https://www.cellsignal.com/products/primary-antibodies/cd200-e5i9v-xp-rabbit-mab-bsa-and-azide-free/24510
IMC / IL23R / Thermo / PA5-102441 / https://www.thermofisher.com/antibody/product/IL23R-Antibody-Polyclonal/PA5-102441
IMC / FAP / R&D systems / AF3715 / https://www.rndsystems.com/products/human-fibroblast-activation-protein-alpha-fap-antibody_af3715
IMC / CD55 / abcam / ab133684 / https://www.abcam.com/en-de/products/primary-antibodies/cd55-antibody-epr6689-ab133684#
IMC / CD1c / OriGene / CF505411 / https://www.origene.com/catalog/antibodies/primary-antibodies/cf505411/cd1c-mouse-monoclonal-antibody-clone-id-oti2f4
IMC / Histone H3 / Standard Biotools / 3176023D / https://store.standardbio.com/Cytometry/ConsumablesandReagentsCytometry/MaxparAntibodies/Anti-Histone%203%20-D1H2-176Yb%E2%80%9425%20%C2%B5g?cclcl=en_US
IMC / ICSK1 / Standard Biotools / TIS-00001 / https://www.standardbio.com/LegacySite_Assets/Documents/Resources/IMC%20Cell%20Segmentation%20Info%20Sheet%20Rev03%20FINAL.pdf
IMC / ICSK2 / Standard Biotools / TIS-00001 / https://www.standardbio.com/LegacySite_Assets/Documents/Resources/IMC%20Cell%20Segmentation%20Info%20Sheet%20Rev03%20FINAL.pdf
IMC / ICSK3 / Standard Biotools / TIS-00001 / https://www.standardbio.com/LegacySite_Assets/Documents/Resources/IMC%20Cell%20Segmentation%20Info%20Sheet%20Rev03%20FINAL.pdf
IN VIVO / CD200 / BioXCell / BE0299 / https://bioxcell.com/invivomab-anti-mouse-cd200-ox2-be0299
IN VITRO / CD200 / Biolegend / 123802 / https://www.biolegend.com/de-de/products/purified-anti-mouse-cd200-ox2-antibody-4422
IN VITRO / Rat IgG2a, κ Isotype Ctrl / Biolegend / 400573 / https://www.biolegend.com/de-de/products/ultra-leaf-purified-rat-igg2a-kappa-isotype-ctrl-7726
FC-sorting ms / CD45 / Biolegend / 103134 / https://www.biolegend.com/de-de/products/brilliant-violet-421-anti-mouse-cd45-antibody-7253
FC-sorting ms / CD11b / Biolegend / 101206 / https://www.biolegend.com/de-de/products/fitc-anti-mouse-human-cd11b-

antibody-347
FC-sorting ms / I-A/I-E / Biolegend / 107636 / https://www.biolegend.com/de-de/products/brilliant-violet-510-anti-mouse-i-a-i-e-antibody-7997
FC-sorting ms / CD301 / Biolegend / 145708 / https://www.biolegend.com/de-de/products/apc-anti-mouse-cd301-mgl1-mgl2-antibody-9127
FC-sorting ms / CD2 / Biolegend / 100108 / https://www.biolegend.com/de-de/products/pe-anti-mouse-cd2-antibody-473
FC-sorting ms / CD11c / Biolegend / 117324 / https://www.biolegend.com/de-de/products/apc-cyanine7-anti-mouse-cd11c-antibody-3931
FC-sorting ms / CD3ε / Biolegend / 100222 / https://www.biolegend.com/de-de/products/apc-cyanine7-anti-mouse-cd3-antibody-6068
FC-sorting ms / CD45R/B220 / Biolegend / 103224 / https://www.biolegend.com/de-de/products/apc-cyanine7-anti-mouse-human-cd45r-b220-antibody-1938
FC-sorting ms / CD197 / Biolegend / 120124 / https://www.biolegend.com/de-de/products/pe-cyanine7-anti-mouse-cd197-ccr7-antibody-13133
FC-sorting ms / Ly6G / Biolegend / 127616 / https://www.biolegend.com/de-de/products/percp-cyanine5-5-anti-mouse-ly-6g-antibody-6116
Cytek-Mo&Fib ms / Podoplanin / Biolegend / 127423 / https://www.biolegend.com/de-de/products/brilliant-violet-421-anti-mouse-podoplanin-antibody-18260
Cytek-Mo&Fib ms / Ly6G / BD Bioscience / 746448 / https://www.bdbiosciences.com/en-de/products/reagents/flow-cytometry-reagents/research-reagents/single-color-antibodies-ruo/bv480-rat-anti-mouse-ly-6g.746448?tab=product_details
Cytek-Mo&Fib ms / CD140a / Biolegend / 135923 / https://www.biolegend.com/de-de/products/brilliant-violet-421-anti-mouse-cd140a-antibody-17921
Cytek-Mo&Fib ms / CD90.2 / Biolegend / 105329 / https://www.biolegend.com/de-de/products/brilliant-violet-570-anti-mouse-cd90-2-thy1-2-antibody-7514
Cytek-Mo&Fib ms / CD2 / BD Bioscience / 740338 / https://www.bdbiosciences.com/en-de/products/reagents/flow-cytometry-reagents/research-reagents/single-color-antibodies-ruo/bv605-rat-anti-mouse-cd2.740338?tab=product_details
Cytek-Mo&Fib ms / CD31 / BD Bioscience / 740483 / https://www.bdbiosciences.com/en-de/products/reagents/flow-cytometry-reagents/research-reagents/single-color-antibodies-ruo/bv650-rat-anti-mouse-cd31.740483?tab=product_details
Cytek-Mo&Fib ms / I-A/I-E / Biolegend / 107643 / https://www.biolegend.com/de-de/products/brilliant-violet-711-anti-mouse-i-a-i-e-antibody-12086
Cytek-Mo&Fib ms / CD45R/B220 / Biolegend / 103246 / https://www.biolegend.com/de-de/products/brilliant-violet-785-anti-mouse-human-cd45r-b220-antibody-7960
Cytek-Mo&Fib ms / CD45 / Biolegend / 103108 / https://www.biolegend.com/de-de/products/fitc-anti-mouse-cd45-antibody-99
Cytek-Mo&Fib ms / CD200 / Biolegend / 123808 / https://www.biolegend.com/de-de/products/pe-anti-mouse-cd200-ox2-antibody-4424
Cytek-Mo&Fib ms / CD49f / Biolegend / 313618 / https://www.biolegend.com/de-de/products/percp-cyanine5-5-anti-human-mouse-cd49f-antibody-5618
Cytek-Mo&Fib ms / CD11c / Biolegend / 117318 / https://www.biolegend.com/de-de/products/pe-cyanine7-anti-mouse-cd11c-antibody-3086
Cytek-Mo&Fib ms / CD11b / Biolegend / 101212 / https://www.biolegend.com/de-de/products/apc-anti-mouse-human-cd11b-antibody-345
Cytek-Mo&Fib ms / CD3ε / Biolegend / 100268 / https://www.biolegend.com/de-de/products/apcfire-810-anti-mouse-cd3-antibody-19553
FC-sorting hu / HLA-DR / Biolegend / 307610 / https://www.biolegend.com/de-de/products/apc-anti-human-hla-dr-antibody-787
FC-sorting hu / CD3ε / Biolegend / 300448 / https://www.biolegend.com/de-de/products/brilliant-violet-510-anti-human-cd3-antibody-9792
FC-sorting hu / CD8a / Biolegend / 344704 / https://www.biolegend.com/de-de/products/fitc-anti-human-cd8-antibody-6149
FC-sorting hu / CD4 / Biolegend / 357414 / https://www.biolegend.com/de-de/products/percp-cyanine5-5-anti-human-cd4-antibody-11856
FC-sorting hu / CD192 / Biolegend / 357210 / https://www.biolegend.com/de-de/products/brilliant-violet-421-anti-human-cd192-ccr2-antibody-8686
FC-sorting hu / CD2 / Biolegend / 300208 / https://www.biolegend.com/de-de/products/pe-anti-human-cd2-antibody-820
FC-sorting hu / CD1c / Biolegend / 331516 / https://www.biolegend.com/de-de/products/pe-cyanine7-anti-human-cd1c-antibody-6245
FC-sorting hu / CD123 / Biolegend / 983702 / https://www.biolegend.com/de-de/products/pe-cyanine7-anti-human-cd123-antibody-15879
FC-sorting hu / CD19 / Biolegend / 302298 / https://www.biolegend.com/de-de/products/spark-plus-uv-395-anti-human-cd19-antibody-24632
FC-Coculture hu / CD3ε / Biolegend / 300448 / https://www.biolegend.com/de-de/products/brilliant-violet-510-anti-human-cd3-antibody-9792
FC-Coculture hu / CD4 / Biolegend / 357414 / https://www.biolegend.com/de-de/products/percp-cyanine5-5-anti-human-cd4-antibody-11856
FC-Coculture hu / CD8a / Biolegend / 344704 / https://www.biolegend.com/de-de/products/fitc-anti-human-cd8-antibody-6149
FC-Coculture hu / IL-17A / Biolegend / 512315 / https://www.biolegend.com/de-de/products/pe-cyanine7-anti-human-il-17a-antibody-5954
Invivo-CD200R1 ms / CD200R / Biolegend / 123918 / https://www.biolegend.com/de-de/products/ultra-leaf-purified-anti-mouse-cd200r-ox2r-antibody-20255

Animals and other research organisms

Policy information about studies involving animals; ARRIVE guidelines recommended for reporting animal research, and Sex and Gender in Research

Laboratory animals	The study involved the following laboratory animals:<br>BALB/c JRj mice (purchased from Janvier labs),<br>C57BL/6 NRj mice (purchased from Janvier labs),<br>C-Kaede-tg (C.Cg-Tg(CAG-tdKaede)15Utr) (purchase from RIKEN laboratory and bred in house),<br>B6-Kaede-tg (B6.Cg-Tg(CAG-tdKaede)15Utr) purchase from RIKEN laboratory and bred in house).<br>All mice used in experimental studies were male or females aged 8-10 weeks old weighing between 18-22g.
Wild animals	The study did not include wild animals.
Reporting on sex	Animals were ranodmized by sex.
Field-collected samples	The study did not include field-collected samples.
Ethics oversight	Mice were maintained under specific pathogen-free conditions and all experiments were performed in accordance to the local regulations and approved by the Regierung von Unterfranken (protocol 55.2-2532-2-1061).

Note that full information on the approval of the study protocol must also be provided in the manuscript.

Clinical data

Policy information about clinical studies

All manuscripts should comply with the ICMJE guidelines for publication of clinical research and a completed CONSORT checklist must be included with all submissions.

Clinical trial registration	no trial
Study protocol	*Note where the full trial protocol can be accessed OR if not available, explain why.*
Data collection	*Describe the settings and locales of data collection, noting the time periods of recruitment and data collection.*
Outcomes	*Describe how you pre-defined primary and secondary outcome measures and how you assessed these measures.*

Flow Cytometry

Plots

Confirm that:

☒ The axis labels state the marker and fluorochrome used (e.g. CD4-FITC).

☒ The axis scales are clearly visible. Include numbers along axes only for bottom left plot of group (a 'group' is an analysis of identical markers).

☒ All plots are contour plots with outliers or pseudocolor plots.

☒ A numerical value for number of cells or percentage (with statistics) is provided.

Methodology

Sample preparation	Single cell samples were created from skin, joint and gut samples by enzymatic dissociation (Collagenase/Dispase/DNAse, outlined in methods). Red cell lysis and debris removal was performed prior to staining.
Instrument	All instruments are from Beckman Coulter: MoFlo Astrios for sorting, Gallios for ex vivo analysis
Software	Gallios Software v.1.2
Cell population abundance	For post sort populations purity was determined by re-analysis for the target population immediately post sorting based on cell surface markers. Purity was >99% for each target population.
Gating strategy	Gating strategies for cell populations is shown in the manuscript data for each cell population. Gates were set to Floresence-1 (FMO controls) or isotype where indicated.

☒ Tick this box to confirm that a figure exemplifying the gating strategy is provided in the Supplementary Information.
